# Antibody CDR amino acids underlying the functionality of antibody repertoires in recognizing diverse protein antigens

**DOI:** 10.1038/s41598-022-16841-9

**Published:** 2022-07-22

**Authors:** Hung-Pin Peng, Hung-Ju Hsu, Chung-Ming Yu, Fei-Hung Hung, Chao-Ping Tung, Yu-Chuan Huang, Chi-Yung Chen, Pei-Hsun Tsai, An-Suei Yang

**Affiliations:** 1 Biomedical Translation Research Center, Academia Sinica, Taipei, Taiwan 155; 2 Genomics Research Center, Academia Sinica, 128 Academia Rd., Sec.2, Nankang Dist., Taipei, Taiwan 115

**Keywords:** Bioinformatics, Applied immunology, Protein design

## Abstract

Antibodies recognize protein antigens with exquisite specificity in a complex aqueous environment, where interfacial waters are an integral part of the antibody–protein complex interfaces. In this work, we elucidate, with computational analyses, the principles governing the antibodies’ specificity and affinity towards their cognate protein antigens in the presence of explicit interfacial waters. Experimentally, in four model antibody–protein complexes, we compared the contributions of the interaction types in antibody–protein antigen complex interfaces with the antibody variants selected from phage-displayed synthetic antibody libraries. Evidently, the specific interactions involving a subset of aromatic CDR (complementarity determining region) residues largely form the predominant determinant underlying the specificity of the antibody–protein complexes in nature. The interfacial direct/water-mediated hydrogen bonds accompanying the CDR aromatic interactions are optimized locally but contribute little in determining the epitope location. The results provide insights into the phenomenon that natural antibodies with limited sequence and structural variations in an antibody repertoire can recognize seemingly unlimited protein antigens. Our work suggests guidelines in designing functional artificial antibody repertoires with practical applications in developing novel antibody-based therapeutics and diagnostics for treating and preventing human diseases.

## Introduction

The antibodies’ biological functions rely on their spectacular capabilities of recognizing cognate antigens with exquisite specificity in a complex aqueous environment filled with a large variety of biomolecules of diverse chemical properties. It has been well-established that the CDRs (complementarity determining regions) of antibodies recognize cognate protein antigens with standard-size antibody–protein interaction interfaces^1^. The aromatic residues (Tyr, Phe, Trp and, to a lesser extent, His) are overly populated among polar residues in the CDRs^2–6^, and the interface propensities for the CDR polar residues are slightly higher or similar to those on solvent accessible protein surfaces^2,7,8^. The antibody–protein interfaces are complementary in geometrical shape^1,8–10^ and electrostatic/chemical composition^8,10–13^, with water molecules stabilizing the interfaces through water-mediated hydrogen bonds^10,12,14–19^. The energetic contributions to the spontaneous formation of antibody–protein complexes have been attributed to a combination of diverse energetic origins, including hydrophobic interactions^11,20,21^, direct hydrogen bonding^12,22,23^, interactions involving aromatic sidechains^24–28^, electrostatic interactions^29–32^, and water mediated hydrogen bonding^17,33–35^. All these interactions need to be considered in the context of solvation by water: firstly, water mediated hydrogen bonding directly or indirectly stabilizing the polar interfacial groups in the combination complexes^17,33,36,37^; secondly, complementary real charges and electric dipoles, including direct hydrogen bonding, with enhanced electrostatic interactions^31,38,39^ due to increasingly diminishing dielectric screening by the increasingly immobilized water molecules near the complex interfaces^17^; thirdly, direct van der Waals contacts of aromatic sidechains on a variety of protein functional groups with corresponding contact energies in aqueous solvation environment^26,27,40^; fourthly, hydrophobic effect by releasing non-structured water molecules around nonpolar carbon atom surfaces into the bulk solvent during forming van der Waals interfacial contacts in the combination complexes^11,20,21^. The former three kinds of driving force are mostly enthalpy-driven, while the latter kind of driving force is largely entropy-driven, frequently leading to a process with thermodynamic manifestation of negative change of Gibb’s free energy ΔG, enthalpy ΔH, entropy ΔS and heat capacity ΔCp for the antibody–protein combination^16,41^. All these driving forces are relevant to the antibody–protein interactions only in the context of water as solvent. As such, water molecules solvating the antibodies and the protein antigens in their native aqueous environment have been recognized as an integral part of the antibody–protein recognition process^42–44^. Although a large body of evidence suggests that only a fraction of the interfacial interactions contribute substantially to the antibody–protein interaction energy^12,24,45,46^, it has not been clear in terms of general principles as to how these energetic contributions underlie the antibodies’ specificity and affinity towards their cognate protein antigens observed in nature. In particular, the coding of the amino acids on the CDRs determining the epitope location on protein antigens and the roles of the water-mediated and direct hydrogen bonding in the formation of the antibody–protein complex interfaces are not completely understood.

This work is aimed to elucidate the shared indispensable elements in the antibody–protein recognitions, so as to understand the essential driving forces underlying the biological functions of antibodies in recognizing their cognate protein antigens. We carried out computational analyses to investigate the principles governing the antibodies’ specificity and affinity towards their cognate protein antigens in the presence of explicit interfacial waters, and then experimentally compared, in four model antibody–protein complexes, the contributions of the interaction types in antibody–protein antigen complex interfaces with the antibody variants selected from phage-displayed synthetic antibody libraries. The results address the phenomenon that natural antibodies with limited sequence and structural variations in an antibody repertoire are capable of recognize seemingly unlimited protein antigens. The insights provide guidelines in designing functional artificial antibody repertoires with practical applications in developing novel antibody-based therapeutics and diagnostics for treating and preventing human diseases.

## Results

### Protein surface water placement prediction algorithms can correctly predict more than two thirds of experimental interfacial waters in known protein–protein complexes

Interfacial water molecules are an integral part of intermolecular interactions in aqueous environment^36,44^, and hence need to be taken into consideration in analyzing the antibody–protein interactions in nature. Since the water structural data in PDB (protein data bank) are incomplete^47^, we predicted interfacial water structures in antibody–protein interaction interfaces of known complex structures with ISMBLab-H2O (computational algorithm developed in our lab ^48^; see “Methods” and Supplemental Methods) and three publicly available solvation water prediction algorithms (Dowser++, Fold-X, and 3D-RISM). Dowser++ water placement is based on rapid docking of water molecules on a rigid protein surface^49^; Fold-X predicts water molecule placement with water structural data on protein surfaces from PDB^50^; 3D-RISM determines water placements by converting theoretical RISM water distribution map into explicit water positions on protein surfaces^51^. Supplementary Fig. S1 shows the exemplary ISMBLab-H2O predictions of water molecule placements around the 20 natural amino acid types, and Supplementary Fig. S2 (A–E) show the exemplary predictions of water molecule placements around an antibody–protein complex with the water molecule placement prediction algorithms.

We benchmarked the surface water placement prediction performances by comparing the prediction results with the protein surface water structures from a data set of 188 protein structures^52^ (Table 1A), and also with the interfacial water structures from a data set of 179 protein-protein interaction complexes^19^ (Table 1B). The overall prediction performances are difficult to be fully assessed for lacking true negative (TN) dataset^47,53^, and hence the F-score (harmonic mean of the precision and recall; see Table 1) is used for evaluating the prediction algorithms. The prediction performance of ISMBLab-H2O is the most balanced in terms of prediction precision and recall, judged by the F-score (F1 = 0.44) in Table 1A. In the protein interfacial water placement prediction results shown in Table 1B, the ISMBLab-H2O’s performance (F1 = 0.38) is the second next to that of Dowser++ (F1 = 0.42) based on the F-score benchmark. However, the META algorithm (see “Methods”) by pooling together the non-redundant water molecule placement predictions from the 4 algorithms is the most informative in terms of examining the potential roles played by the interfacial water molecules by considering all the possible interfacial water positions based on diverse theoretical perspectives. As shown in Table 1B, the META predictions cover two thirds of the known interfacial water positions in the experimental dataset, as judged by the recall measurement (Rec = 0.68, Table 1B), which is much higher than the results of random predictions with the RANDOM prediction algorithm (see “Methods”) (Rec = 0.29, Table 1B). In the following work, we applied the META interfacial water molecule placement predictions for the analyses of the effects of interfacial water molecules in antibody–protein interaction interfaces.

**Table 1:** Water molecule placement prediction performances benchmarked with water structures derived from X-ray crystallography.

	TP	FN	FP	F1	Pre	Rec
**(A)**						
ISMBLab-H2O	29,440	36,016	38,902	0.44	0.431	0.45
Fold-X	11,791	53,665	16,149	0.252	0.422	0.18
3D-RISM	44,227	21,229	160,165	0.328	0.216	0.676
Dowser++	9641	55,815	6993	0.25	0.58	0.16
**(B)**						
ISMBLab-H2O	2026	2736	3959	0.38	0.34	0.43
Fold-X	1756	3006	3671	0.35	0.32	0.37
3D-RISM	2957	1805	9087	0.35	0.25	0.62
Dowser++	2192	2570	3442	0.42	0.39	0.46
RANDOM(I)	1378	3384	5005	0.25	0.22	0.29
RANDOM (II)	1402	3360	5086	0.25	0.22	0.29
RANDOM (III)	1359	3403	5001	0.24	0.21	0.29
META	3251	1511	8644	0.39	0.27	0.68

The ISMBLab-H2O benchmark results are compared side-by-side with those predicted with three other water molecule placement prediction algorithms: Dowser++^49^, Fold-X^50^, and 3D-RISM^51^. The prediction performances are assessed in Table (A) by comparing the prediction results with the protein surface water structures from a data set of 188 protein structures^52^, and in Table (B) by comparing the prediction results with the water structures in the interfacial spaces (see “Methods”) of 179 protein–protein interaction complexes^19^. The ISMBLab-H2O, META and RANDOM prediction methods are described in “Methods”. TP: true positive, as defined by the experimentally determined water oxygens, each of which is close to at least one predicted water oxygen within the distance threshold of vdW(H_2_O) = 1.4 Å. FN: false negative, as defined by the experimentally determined water oxygens that have no nearby (defined by the same threshold above in TP) predicted water oxygens. FP: false positive, as defined by the predicted water oxygens that have no nearby (defined by the same threshold above in TP) experimentally determined water oxygens. F1: F-score, as defined by F = 2 × Pre × Rec/(Pre + Rec), where Pre (Precision) is defined as Pre = TP/(TP + FP) and Rec (Recall) is defined as Rec = TP/(TP + FN).

### Natural antibodies form stereospecific complex structures with diverse protein antigens through interfacial aromatic and direct/water-mediated polar interactions driven by the corresponding amino acids encoded in two respective groups of prominent CDR residue positions

**Figure 1:**
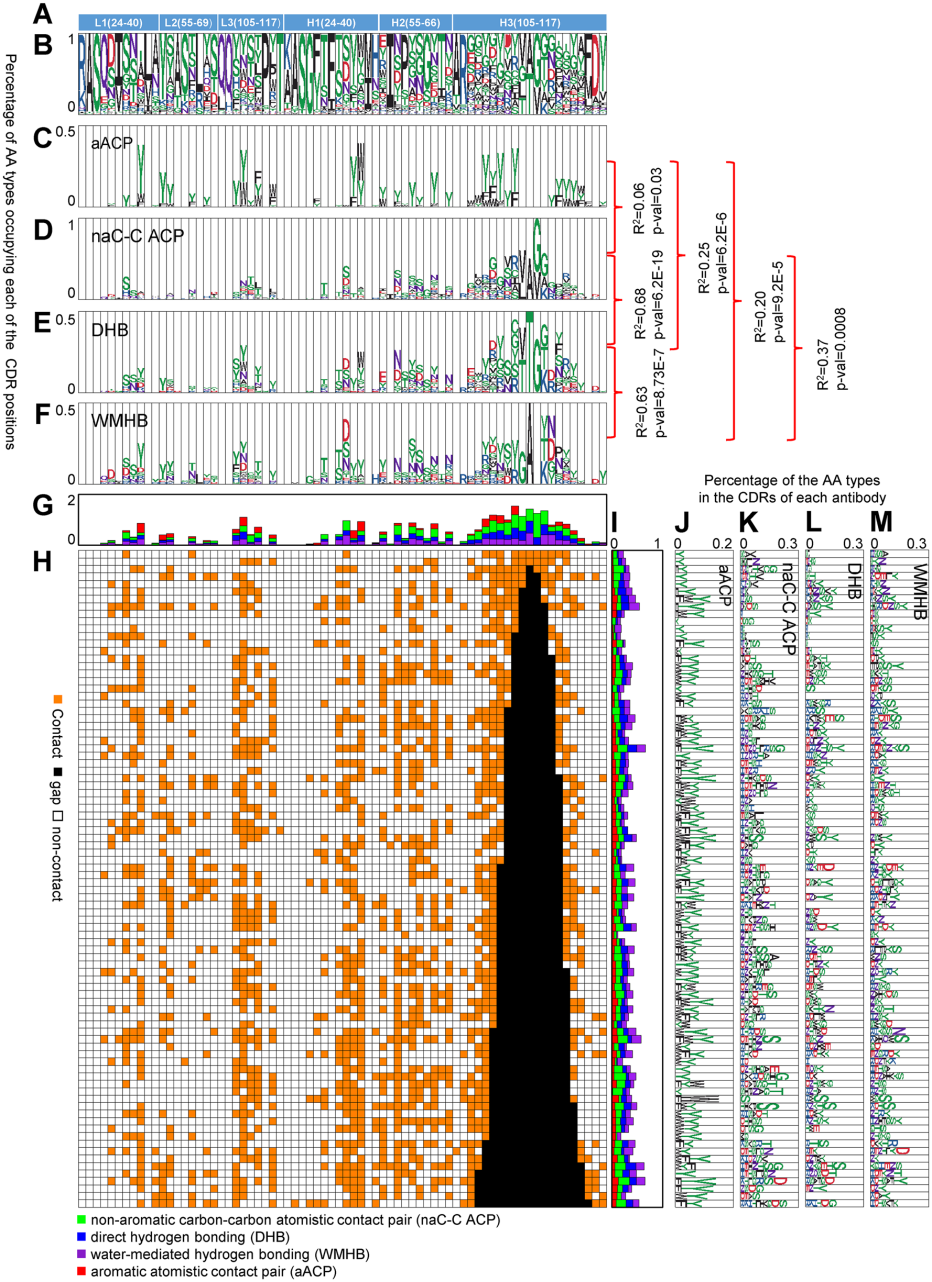
Amino acid types in the CDR residue positions of the antibody–protein antigen complexes of S88 dataset. (**A**) The x-axis indicates the CDR positions marked by IMGT numbering. (**B**–**F**) The y-axis shows the antibody (Ab) amino acid (AA) type percentage (out of the complex structures in S88 dataset) distributions at each CDR position for the Ab AA types in panel (**B**); for the Ab AA types with the Ab residues’ aromatic carbons interacting with the protein antigens (Ag) with atomistic contact pairs (aACPs) in panel (**C**); for the Ab AA types with the non-aromatic Ab residues’ carbons interacting with the Ag carbons with atomistic contact pairs (naC-C ACPs) in panel (**D**); for the Ab AA types involving in Ab-Ag direct hydrogen bonds (DHBs) in panel (**E**); for the Ab AA types involving in Ab-Ag water-mediated hydrogen bonding (WMHB) in panel (**F**). (**G**) The y-axis shows the cumulative stacking of magnitudes in panels (**C**–**F**). (**H**) The x-axis of the contact map shows the CDR positions (IMGT numbering); the y-axis shows the complex structures in the S88 dataset with PDB codes and CDR amino acid sequences listed in Supplementary Table S1. The orange-colored elements of the contact map indicate at least one of the interactions shown in panels (**C**–**F**). (**I**) The x-axis shows the cumulative stacking of magnitudes in panels (**J**–**M**). (**J**–**M**) The x-axes of the histograms show the Ab AA type percentage (out of the CDR positions in each complex structure) distributions of aACPs, naC-C ACPs, Ab-Ag DHBs, and Ab-Ag WMHBs, respectively for each Ab-Ag complex structure (y-axis). R^2^ is the square of the Pearson’s correlation coefficient calculated by comparing the two magnitude arrays of y-axis values of the two histograms and the P-values were calculated with Student’s test. ACP, DHB and WMHB are described in “Methods”.

One key question on the antibody function is: How do natural antibodies with relatively limited variations of CDR canonical structures (CSs) encoded with relatively limited amino acid types in comparison with the vast sequence and structural diversities of proteins recognize almost unlimited protein antigens? To address this question, we first analyzed 88 non-redundant antibody–protein antigen complexes from PDB (S88 dataset, see “Methods” and Supplementary Table S1). The antibody structures in the S88 dataset are limited in canonical structures for CDRH1–H2–L1–L2–L3 to the combination of CS type 1–2–2–1–1, which is the most prominent structural class of antibodies in nature^54–56^, and all the epitopes on the antigen proteins are conformational epitopes (see “Methods”). Conformation of the CDRH3 varies and the amino acid sequence length ranges from 5 to 21 residues. By controlling the antibody structural variations in CDRH1–H2–L1–L2-L3 and following the IMGT definition of the equivalent positions in CDRH3 (Fig. 1A), we statistically analyze the interfacial interactions specific for each CDR positions. Since the interfacial water molecules are an integral part of the protein–protein interaction interfaces^36,44^, the analyses were carried out in the presence of interfacial waters predicted with the META algorithm described above.

Amino acid residues from a subset of the CDR residue positions are frequently involved in interfacial contacts in the antibody–protein complex structures. The CDR residue positions and the distributions of the amino acid types in these CDR residue positions are shown in Fig. 1A and B respectively. The interfacial contacts in each of the complex structures in S88 are shown in Fig. 1H and a few of the structural examples of the interfacial contacts are shown in Supplementary Fig. S2F–H. Since the antibody–protein recognitions are energetically attributed mostly to (1) aromatic interaction, (2) hydrophobic interaction, and polar interaction with (3) direct or (4) water-mediated hydrogen bonding (see Introduction), we respectively analyzed the amino acid type distributions (Fig. 1C–F) in each of the CDR residue positions involving in these four types of interfacial interactions. The results indicate that the CDR residues forming interfacial contacts are distributed heavily on the CDR residue positions that, expectedly, are the most exposed to the surface of the CDRs (Fig. 1G). Also, expectedly, tyrosine is the most prominent CDR amino acid type involving in aromatic interactions^2^ (Fig. 1C), and amino acid types with polar sidechains are used in the exposed CDR residue positions to form direct or water-mediated hydrogen bonds bridging the antibody–protein interfaces (Fig. 1E and F respectively). Moreover, Fig. 1I–M indicates that, although the four types of interfacial interactions are all ubiquitous in the antibody–protein complexes, the constituent fractions of the interaction types are highly diverse among the antibody–protein interfaces, suggesting that the predominant interaction type driving the complex formation is not immediately identifiable from the interaction type distributions among the complex interfaces.

Unexpectedly as shown in Fig. 1D, CDR residues forming non-aromatic carbon–carbon atomistic contact pairs bridging the interfacial contacts are amino acid types with polar sidechains, rather than amino acid types with hydrophobic sidechain. The histogram in Fig. 1D is highly correlated with that of the CDR residues with interfacial direct hydrogen bonds (Fig. 1E) with R^2^ = 0.68 (P-value = 6.2 × 10^–19^), indicating that the non-polar contacts in the antibody–protein complex interfaces are mostly driven by the interfacial direct hydrogen bonding, rather than by tightly packed hydrophobic sidechains as in protein interiors or permanent protein–protein interaction interfaces. Moreover, the CDR position distribution shown in Fig. 1D is insignificantly correlated with that of the aromatic residues involving interfacial aromatic interactions (Fig. 1C) with R^2^ = 0.06 (P-value = 0.033), indicating that the CDR residues involving the aromatic interfacial contacts are distributed differently in terms of CDR residue positions from the CDR residues involving direct hydrogen bonds bridging the antibody–protein interfaces. These results indicate that, in the antibody–protein complex interfaces, aromatic interactions involving CDR aromatic residues, in particular tyrosine, are much more prevalent in comparison with the conventional hydrophobic interactions involving tightly packed hydrophobic sidechains.

The interfacial water-mediated hydrogen bonding pairs (total 771 pairs in S88 dataset) are more prevalent than interfacial direct hydrogen bonding pairs (total 605 pairs in S88 dataset), emphasizing the importance of water-medicated hydrogen bonding bridging the antibody–protein interfaces. The histograms in Fig. 1E and F are highly correlated with R^2^ = 0.63 (P-value = 8.7 × 10^–7^), indicating that the same set of CDR residue positions are used for direct and water-mediated hydrogen bonds bridging the antibody–protein interfaces. This correlation is not due to overlapping of CDR residues involving direct and water-mediated hydrogen bonding simultaneously—only a total of 73 pairs of polar interactions are simultaneously connected by direct and water-mediated hydrogen bonding. Given that an average antibody complex structure in S88 has 17 CDR residue positions contacting its cognate antigen (Fig. 1I), each of the antigen-contacting CDR residues has on average 0.9 direct (0.4/CDR position) or water-mediated (0.5/CDR position) hydrogen bond bridging the interface. The results indicate that the interfacial polar interactions contain extensive direct/water-mediated hydrogen bonding in the presence of interfacial aromatic interactions involving mostly tyrosine sidechains on CDRs; with further comparative studies (see the following section), we can evaluate the level of optimization of these interfacial polar interactions in S88 dataset.

Evidently, the antibody–protein recognitions are essentially dominated by two types of interactions: firstly, the interfacial contacts involving CDR aromatic amino acid types as shown in Fig. 1C and polar amino acid types in Fig. 1D–F. These two groups of amino acid type are encoded in two sub-groups of CDR residue positions, which simultaneously contribute to the interfacial contacts. Moreover, tyrosine is the most prevalent aromatic and polar amino acid type compatible with both groups of interactions, highlighting the importance of the tyrosine sidechains in the CDRs for antibody–protein recognitions. Still, two related questions need to be further addressed: firstly, whether the interfacial polar interactions are optimized in terms of hydrogen bonding in the antibody–protein complexes of S88; secondly, which one of the two interaction types is the predominant determinant deciding the epitope location on the cognate antigen.

### Interfacial polar interactions involving direct/water-mediated hydrogen bonding and pairwise amino acid type contact preferences, rather than geometrical complementarity of the CDR structures with the cognate protein antigens, contribute to determine stereospecific antibody–protein complexes

In this section, we want to investigate if optimization of polar interactions in terms of hydrogen bonding is more important than optimization of geometrical complementarity in determining the complex structure stereospecificity in the antibody–protein complex interfaces. This question is important because geometrical complementarity has been considered as one of the main driving forces determining the specificity of protein–protein interactions^1,8–10^. To this end, we compared the interfacial polar interactions in S88 dataset with those in a set of randomly generated complex structures, dubbed S880 dataset. We assembled S880 dataset using each of the S88 antibody-antigen pairs to generate 10 randomly docked complex structures with the docking algorithm PatchDock^57^ by optimizing the geometrical complementarity of the CDRs of the antibody binding to non-native epitopes of the cognate protein antigen (see “Methods”).

The S880 dataset is used as a null hypothesis versus the positive control S88 dataset, so as to elucidate the level of optimization of the interfacial polar interactions in natural antibody–protein complexes in comparison with the null hypothesis. Interfacial interactions can be measured in various ways. In this work, following the framework of the statistical analyses shown in Fig. 1, we quantify the level of interfacial interactions by counting non-redundant atomistic contact pairs (ACP) and direct/water-mediated hydrogen bonds (HBs), namely aACP, naC-C ACP, DHB, and WMHB as shown throughout this work and described in “Methods”. The effect of interfacial waters as part of the interfacial complementarity is explicitly measured with WMHB throughout the work.

Artificially generated antibody–protein complexes in the S880 dataset have more extensive interfacial contacts but less optimized polar interactions comparing with the native complex structures in the S88 dataset. Antibody CDRs of the antibody–protein complexes in S880 have about 2.7-fold of interacting interfacial contacts per complex structure in comparison with those in S88 (Supplementary Fig. S3A), indicating that the natural antibodies use, on average, only a small fraction of the surface-exposed atoms on CDR residues for protein antigen recognitions. The distributions of the predicted interfacial waters normalized by the interfacial contacts are almost identical between the interfaces in S88 and S880 (Supplementary Fig. S3H), indicating that both native and artificially generated interfaces are equally solvated by the predicted interfacial waters. However, the normalized direct hydrogen bonds and water-mediated hydrogen bonds in the complex interfaces in S880 are reduced by 2.9- and 1.6-fold, as shown in Supplementary Fig. S3K and L respectively, and the normalized unfavorable polar contacts involving non-aromatic carbon-polar atomistic contacts and hydrogen bond donor-donor or acceptor-acceptor atomistic contacts are increased by 1.2- and 1.4-fold (Supplementary Fig. S3M and N respectively) in S880 in comparison with those in S88. These results indicate that the interfacial polar interactions in the native complexes are more optimized in comparison with those in the artificially generated interfaces. The averaged predicted interfacial waters in the bound form of the complexes are about 40 ~ 47% of those in the non-bound form of the complex structures in both S88 and S880 (Supplementary Fig. S3H), indicating that forming antibody–protein interfaces are accompanied with removal of more than half of the solvation waters around the interface atoms in the complex structures in S88 and S880. The desolvation in forming the antibody–protein interfaces is better compensated by the optimal interfacial polar interactions of the native antibody–protein interfaces in S88 in comparison with those in S880. Furthermore, the normalized contacts involving CDR aromatic amino acid sidechains are reduced to 73% (Supplementary Fig. S3I) and the non-aromatic carbon–carbon contacts are increased by 1.2-fold (Supplementary Fig. S3J) in S880 in comparison with those in S88, supporting that the artificially generated complex structures are less optimized in contacts involving CDR aromatic sidechains and are more extensive in random contacts involving non-aromatic carbon atoms.

Native interfacial polar interactions in S88 retain substantial chemical complementarity between the CDR polar sidechains and the interacting sidechains on the cognate protein antigen. The interfacial chemical complementarity can be quantitatively measured with the pairwise amino acid type contact preferences, P_n_(x,y), which is the log-odd ratio of pairwise amino acid type contact preference for amino acid type x in antibodies to interact with amino acid type y in the corresponding protein antigens for contact group n: n = 1 for carbon–carbon atomistic contacts; n = 2 for direct hydrogen bonding contacts; n = 3 for water-mediated hydrogen bonding contacts, as defined in Eq. (2) in Supplemental Methods. The log-odd ratios are calculated with the distributions of contact pairs in S88 versus those in S880; that is, the interfacial contact pair distributions in S880 are used as null hypotheses. Supplementary Fig. S4 shows the results of P_n_(x,y), indicating that the chemical complementarity does contribute to the stereospecificity of the native antibody–protein complex structures to an extent. Many of the large positive P_n_(x,y) values can be understood by amino acid contact pair preferences in the interfaces of protein–protein interactions^18,19,58^. Specifically, positively charged residues (RKH) interact favorably with negative changed residues (DE) through electrostatic interactions (red boxes in Supplementary Figure S4); histidine residues (H) interact favorably with aromatic residues (FWY) through aromatic ring interactions (blue boxes in Supplementary Fig. S4); hydrophobic residues (ALIVMF) interact favorably with hydrophobic residues (green box in Supplementary Fig. S4); aromatic residues (FWY) interact favorably with positively charged residues (RK), aromatic residues (FWY) and mainchains of proline and glycine residues (PG) through aromatic ring-cation, aromatic ring-ring, and aromatic ring-peptide bond interactions respectively (orange and yellow boxes in Supplementary Fig. S4). Moreover, the Pearson’s correlation coefficient between P_2_(x,y) and P_3_(x,y) in Supplementary Fig. S4 is 0.51, indicating that interfacial direct hydrogen bonded sidechain pairs and water-mediated hydrogen bonded sidechain pairs share common chemical complementarity to an extent, such as electrostatic interactions involving real charges and electric dipoles. This correlation, albeit marginally significant, is striking, given that the two interacting groups share only around 10% of overlap. By contrast, P_1_(x,y) and P_2_(x,y) in Supplementary Fig. S4 are not as correlated (Pearson’s correlation coefficient = 0.23), highlighting the importance of the interacting sidechain chemical complementarity even in non-direct polar interactions such as water-mediated hydrogen bonding.

In summary, the results indicate that the interfacial polar interactions in native antibody–protein complexes are optimized to form direct or water-mediated hydrogen bonding, and these polar interactions retain substantial chemical complementarity among the interacting sidechains, such as electrostatic complementarity. In addition, the native antibody–protein complex interfaces are usually limited to only a small portion (about one third on average) of the potential optimal contacts of the CDRs forming maximal complementarity in geometrical shape as in the artificially generated antibody–protein complexes in S880, likely due to the energetic balance in optimizing interfacial polar/aromatic interactions while avoiding desolvation accompanied with the formation of the antibody–protein complexes. These results suggest that the optimal interfacial polar/aromatic interactions, rather than maximization of the extent of van der Waals contacts in the interfaces, contribute as determinants for the stereospecificity of the natural antibody–protein complex structures, for which the interfaces are less compact in comparison with those in homodimers and permanent protein complexes^8,9^. This conclusion is supported in that PatchDock has been reasonably successful in modelling interfaces in homodimers and permanent protein complexes^57^ and yet is not likely to predict the epitope locations with the same set of geometrical complementarity criteria proven useful in predicting the interfaces in homodimers and permanent protein complexes. Although the conclusion is derived from the comparison of S88 dataset with S880 dataset as the null hypothesis, which was constructed with rigid body docking as a first approximation, the modeling uncertainty is only to argue for more extensive geometrical complementarity in the artificial complex structures, not less.

### Some CDR aromatic residues are key components for the predominant determinant in determining antibody–protein interaction specificity and affinity for antibodies from nature and from artificial antibody libraries unlimited by germline antibody sequences and natural antibody maturation

The previous two sections establish that aromatic, in particular tyrosine, and polar/charged sidechains are the two groups of main paratope constituents for protein antigen recognition. But the key question remains unanswered: which one of the two CDR amino acid type groups is the predominant determinant for the epitope location on a protein antigen recognized by its cognate antibody? To address this question, we investigated the CDR amino acid type preference profiles of the CDR residues contacting the cognate protein antigen in two known antibody–protein complex structures (G6-VEGF and F10-HA, see below). The CDR amino acid type preference profiles were attained by first experimentally enumerating the amino acid types of the CDR residues in only one CDR with the NNK degenerate codon to construct phage-displayed synthetic antibody libraries and followed by determining the CDR sequences of the antibody variants in the antibody libraries binding to the cognate antigen. The experimental process was carried for each of the CDRs under consideration. Since only one CDR was altered with the other 5 CDRs unaltered in each of the variants, the binding mode of the antibody variants to the target protein antigen was expected to be unaltered as well, as had been proven with competitive ELISA (Enzyme-Linked Immuno-Sorbent Assay) in the presence of the parent antibody^48^. To quantify the statistical significance of the CDR sequence preference profiles, we statistically compared the CDR sequence profiles with null hypothesis datasets, where the amino acid type distribution probabilities for the degenerate codon NNK (p_i_ of Eq. (3) in Supplemental Methods) were applied to generate 1000 random CDR variants with computationally modeled complex structures based on the corresponding parent complex structure with default Fold-X^59^ modelling algorithm and parameters. These computational structures form a dataset for each of the two known complex structures of G6-VEGF and F10-HA as the null hypothesis datasets. To validate the quality of the computational modeling of the complex structures with the CDR variants, we assessed the modeled complex structures in terms of the impacts of modeling uncertainties to the results of the statistical analyses shown in Supplementary Fig. S5. The technical details of the construction of the datasets of computationally modelled complex structures and the assessments of the impact of the uncertainty of the computational structures on the quantitative analyses based on the modelled structures are described in “Methods” and Supplementary Fig. S5. The results indicate that the computational modeling uncertainties have insignificant influence on the quantitative conclusions of the statistical analyses.

**Figure 2:**
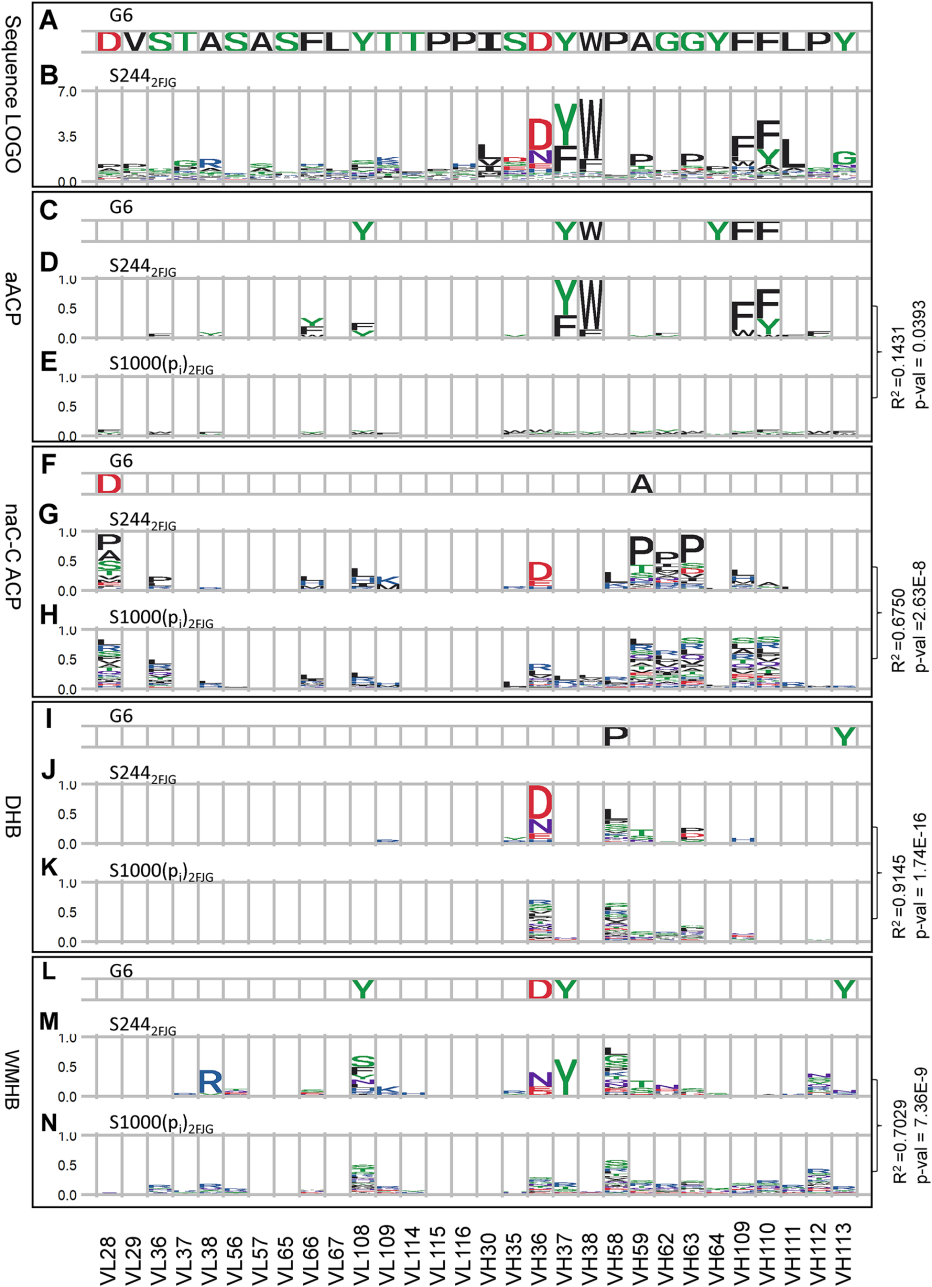
CDR sequence preferences for the interface residues in G6-VEGF complex structure (PDB code:2FJG). Complex structures of the 244 CDR sequence variants shown in Supplementary Table 1A–E in Yu et al.^48^ were modelled with default Fold-X structural modeling algorithm^59^ based on the G6(Ab)–VEGF(Ag) complex structure in PDB code:2FJG^60^ (see “Methods”). These computational structures form the dataset S244_2FJG_ as the positive control group. (**A**) The amino acid sequence of the G6 CDR residues are shown with the IMGT numbering in the x-axis. (**B**) The CDR sequences in S244_2FJG_ were used to calculate the antibody sequence LOGO (d_ji_ of Eq. (3) in Supplemental Methods) (y-axis) for the CDR positions (x-axis). The amino acid type distribution probabilities for the degenerate codon NNK (p_i_ of Eq. (3) in Supplemental Methods) were applied to generate 1000 CDR sequence variants for the computational complex structures built with Fold-X (see “Methods”). These computational structures form the dataset S1000(p_i_)_2FJG_ as the null hypothesis control group. (**C**) This panel shows the aromatic residue (FWY) positions and amino acid types in G6 forming aACPs with VEGF in the experimental complex structure. (**D**) The y-axis shows the percentage of the antibody aromatic residue (FWY) at each of the CDR positions (x-axis) involving aACPs with VEGF in S244_2FJG_ dataset; (E) The y-axis shows the percentage of the antibody aromatic residue (FWY) at each of the CDR positions (x-axis) involving aACPs with the VEGF in S1000(p_i_)_2FJG_ dataset. (**F**–**H**) These panels follow the same description as in the panels in (**C**–**E**) for the percentages of amino acid types involving naC-C ACPs calculated with S244_2FJG_ dataset (panel **G**) and S1000(p_i_)_2FJG_ dataset (panel **H**). Similarly, panels in (**I**–**K**) and panels in (**L**–**N**) show the percentages of amino acid types involving DHBs and WMHBs calculated with S244_2FJG_ dataset (panels **J** and **M**) and S1000(p_i_)_2FJG_ dataset (panels **K** and **N**) respectively.

The indispensable interactions in the antibody G6 to VEGF (vascular endothelial growth factor) complex structure^60^ involve mainly a subset of aromatic residues on the CDRs of G6^48^. In the G6-VEGF complex structure^60^, 6 aromatic residues in CDRs (Fig. 2A) contact with VEGF (Fig. 2C). Sequence LOGO (Fig. 2B) of the VEGF-binding CDR variants indicates that 4 CDR aromatic residues (VH37, VH38, VH109 and VH110 in Fig. 2D) are highly conserved for the native paratope-epitope combination defined by the G6-VEGF complex structure^60^. The preference profiles of these 4 CDR aromatic residues (Fig. 2D) are substantially different from the profiles generated by the null hypothesis dataset (R^2^ = 0.14, P-value = 0.039) (Fig. 2E), indicating that the preferences for the aromatic residues in these CDR positions (Fig. 2D) are essential for the native G6-VEGF complex formation and are not likely to occur by chance as shown in Fig. 2E. In contrast, Fig. 2G is highly similar to the profile generated by the null hypothesis dataset for VEGF binding (R^2^ = 0.68, P-value = 2.6 × 10^–8^) (Fig. 2H), suggesting that the complex structure determines the contact profiles in Fig. 2G and H. The result indicates that the non-aromatic carbon–carbon contacts impose little constrain on the amino acid type requirements for the formation of the native G6-VEGF complex structure, implying that these contacts in the native G6-VEGF complex structure are not a major determinant for the stereospecificity of the complex structure. Similarly, the comparable profile pairs derived from the positive and null hypothesis datasets for the distributions of the CDR amino acids involving direct hydrogen bonding (Fig. 2J and K; R^2^ = 0.91, P-value = 1.7 × 10^–16^) and water-mediated hydrogen bonding (Fig. 2M,N; R^2^ = 0.70, P-value = 7.4 × 10^–9^) indicate that these two types of polar interactions involving in the native G6-VEGF complex structure are also a necessary condition for forming the native complex structure, suggesting that the direct/water-mediated hydrogen bonds are not likely to predominantly underlie the specificity of the native G6-VEGF complex structure. Understandably, the direct/water-mediated hydrogen bonding can easily form from the ubiquitous hydrogen bond donors/acceptors in polar sidechains, mainchain peptide groups and solvation water molecules, as demonstrated by the random complex structures in the null hypothesis dataset (Fig. 2K,N). Together, the results in Fig. 2 suggest that the stereospecific G6-VEGF complex structure is mostly determined by the 4 highly indispensable aromatic residues contacting VEGF. Other interactions associated with the formation of the complex structure could further stabilize the native complex structure, but, in contrast to the indispensable aromatic interactions, the other interactions per se are not likely to predominantly underlie the specificity of the native complex structure.

**Figure 3:**
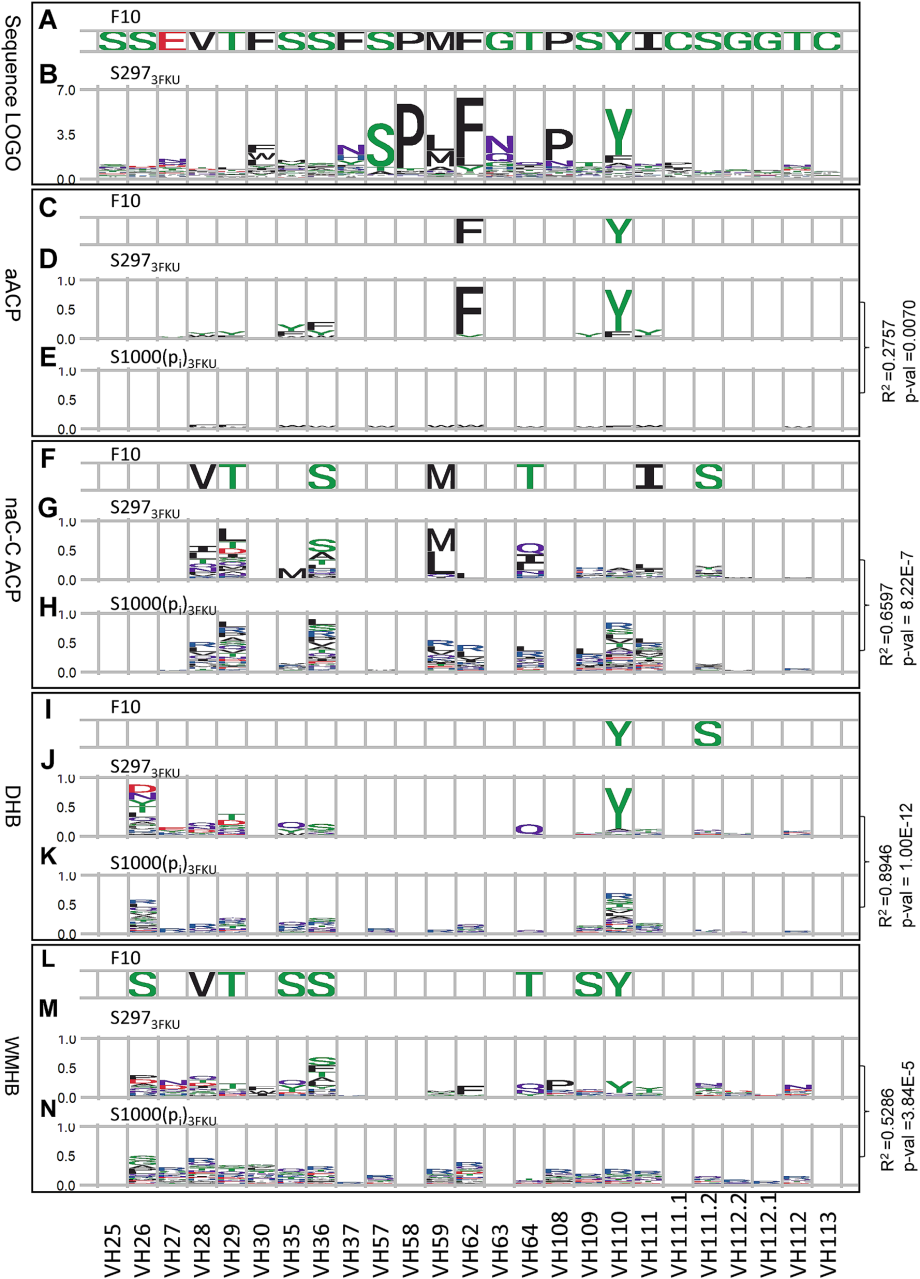
CDR sequence preferences for the interface residues in F10-HA complex structure (PDB code:3FKU). Complex structures of the 381 CDR sequence variants shown in Supplementary Tables S2–S4 in Tung et al.^62^ were modelled with default Fold-X^59^ based on the F10-HA complex structure in PDB code: 3FKU^61^. These computational structures form the dataset S381_3FKU_ as the positive control group for HA binding. The amino acid type distribution probabilities for the degenerate codon NNK (p_i_ of Eq. (3) in Supplemental Methods) were applied to generate 1000 CDR sequence variants for the computational complex structures built with Fold-X. These computational structures form the dataset S1000(p_i_)_3FKU_ as the null hypothesis control group for HA binding. The panels (**A**–**N**) were calculated as described in Fig. 2 with S381_3FKU_ S1000(p_i_)_3FKU_ datasets.

The conclusion above is consistent with that from the F10-HA (influenza hemagglutinin) antibody–protein complex structure^61^, for which the indispensable interactions of the antibody F10 binding to HA have been elucidated with the same experimental procedure^62^. Since the light chain of F10 does not involve in the interface of the native F10-HA complex structure^61^, only the heavy chain CDR positions were investigated for the CDR amino acid preferences in the F10-HA combination shown in Fig. 3. The results in Fig. 3 are similar to those in Fig. 2 in conclusion: two of the CDR aromatic residues (VH62 and VH110 in Fig. 3D comparing with (E); R^2^ = 0.28, P-value = 0.007) are indispensable for the specific binding of F10 to HA. The other three types of interactions are not likely to predominantly underlie the specificity of the native complex structure, judging by the similarity of the sequence profile pairs derived from positive and null hypothesis datasets, as shown in comparison of Fig. 3G with H (R^2^ = 0.66, P-value = 8.2 × 10^–7^), Fig. 3J with K (R^2^ = 0.89, P-value = 1.0 × 10^–12^), and Fig. 3M with N (R^2^ = 0.53, P-value = 3.8 × 10^–5^). The conservativeness of serine and proline in VH57 and VH58 in the sequence LOGO (Fig. 3B) is likely due to the CDR structural requirements, rather than antigen binding, because these two residues do not contact with the antigen based on the native F10-HA complex structure.

**Figure 4:**
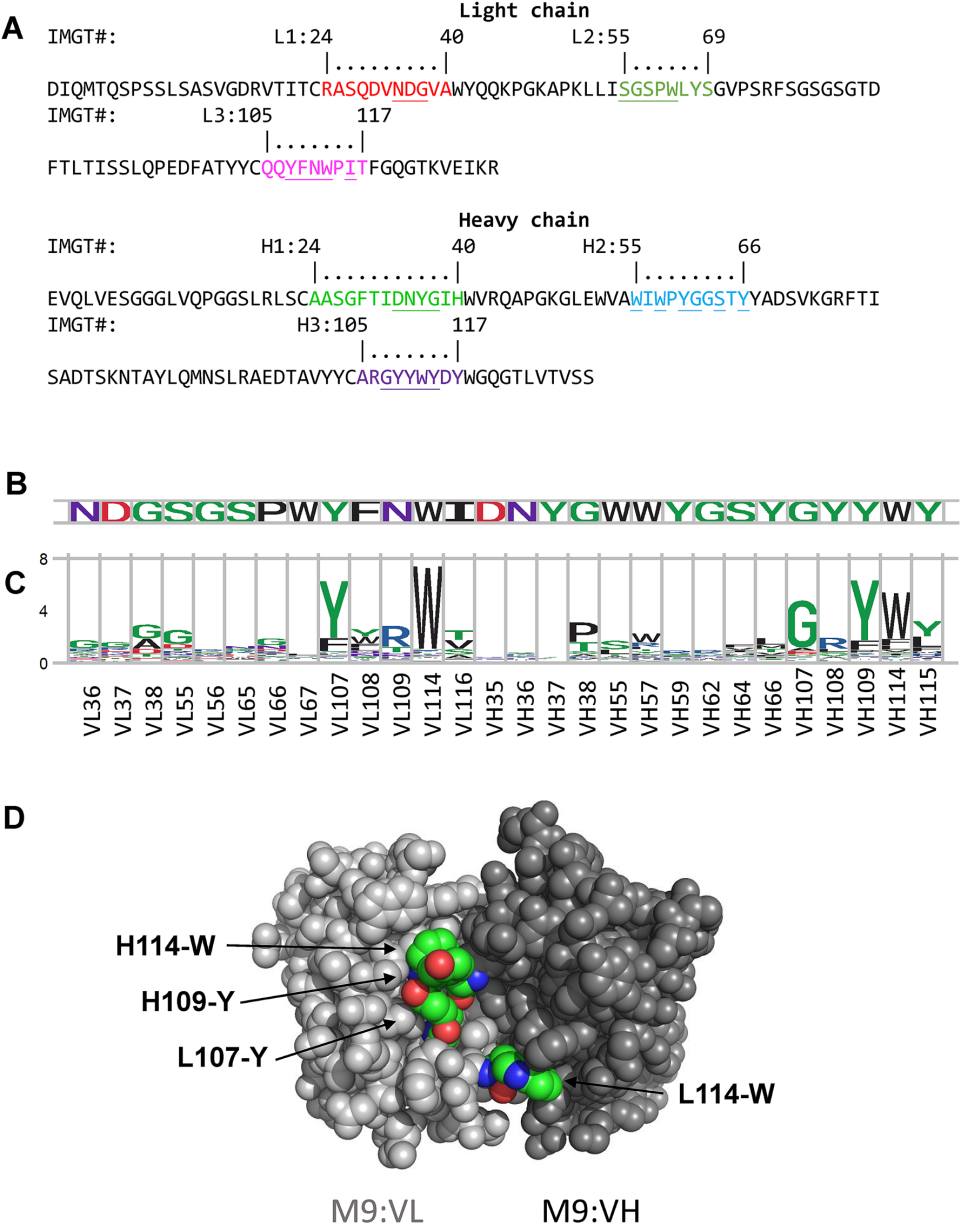
Amino acid type preferences for antibody M9 binding to MSLN. Antibody M9 binding to mesothelin (MSLN) had been attained from the phage-display synthetic antibody libraries developed in our lab^55^. (**A**) The sequences of the VL and VH domains of M9 are shown with the CDRs highlighted in colors with IMGT numbering. (**B**) The subset of CDR positions (x-axis) in M9 (underlined in the sequences in (**A**)) were enumerated in degenerate codon NNK with phage-displayed synthetic antibody libraries, and the CDR variants of M9 binding to the native epitope of MSLN were selected and screened from the phage-displayed synthetic antibody libraries. The experimental procedure was followed without modification as in our published works^48,62^. The CDR sequences of these CDR variants of M9 are listed in Supplementary Table S4. (**C**) The amino acid type preferences for the MSLN-binding CDR variants of M9 are shown as sequence LOGO (Supplemental Methods) for each of the CDR positions (x-axis), calculated with the CDR sequences of the CDR variants of M9 in Supplementary Table S4. (**D**) The antibody M9 VL (colored in grey) and VH (colored in black) variable domain structures were computationally modelled with RosettaAntibody modeling software^63^ with default parameters. The four CDR positions highly conserved in aromatic amino acid types are highlighted in green for the aromatic carbon atoms.

**Figure 5:**
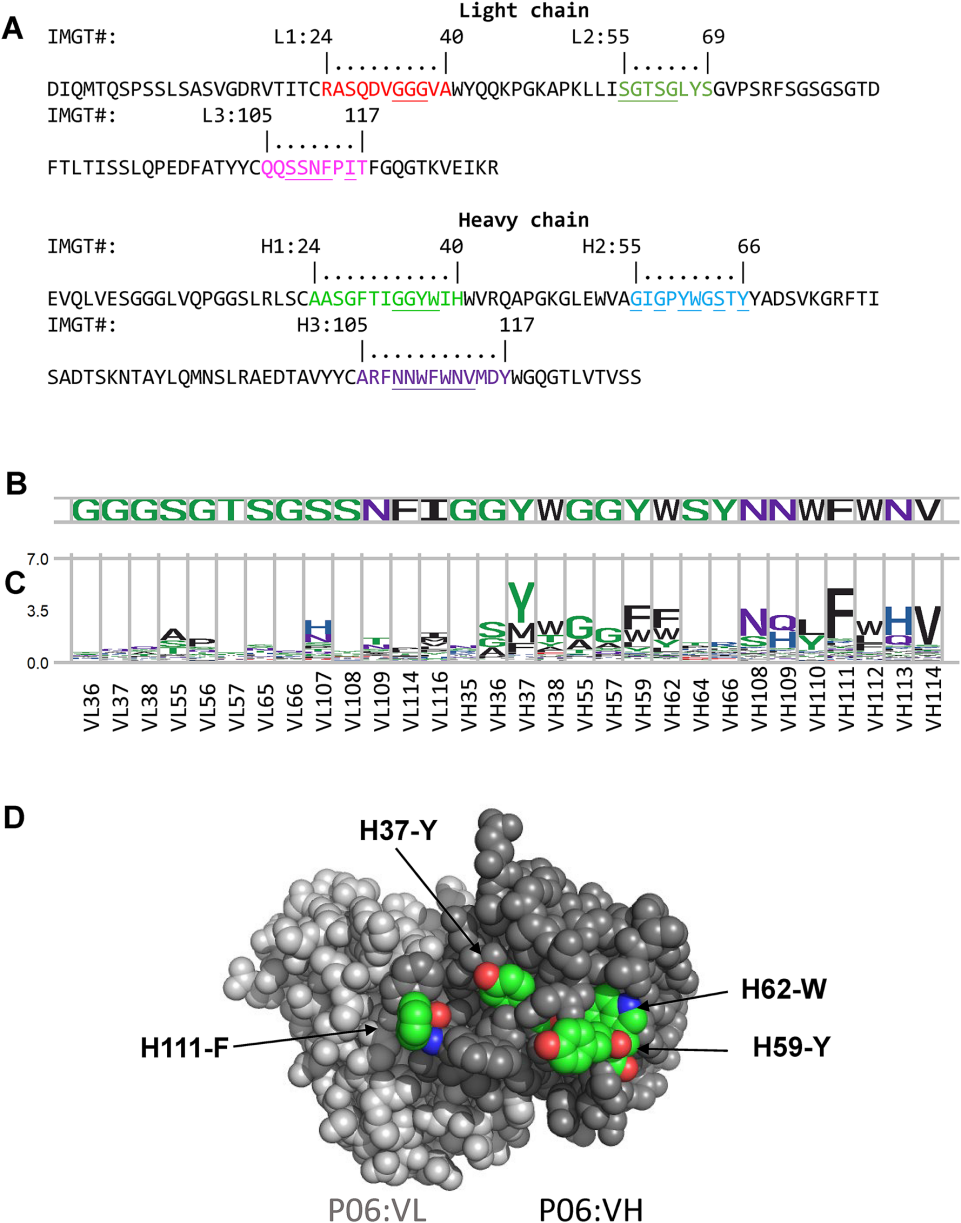
Amino acid type preferences for antibody P06 binding to HA. Antibody P06 binding to influenza hemagglutinin (HA) had been attained from the phage-display synthetic antibody libraries developed in our lab^55,64^. (**A**) The sequences of the VL and VH domains of P06 are shown with the CDRs highlighted in colors with IMGT numbering. (**B**) The subset of CDR positions (x-axis) in P06 (underlined in the sequences in (**A**)) were enumerated in degenerate codon NNK with phage-displayed synthetic antibody libraries, and the CDR variants of P06 binding to the native epitope of HA were selected and screened from the phage-displayed synthetic antibody libraries. The experimental procedure was followed without modification as in our published works^48,62^. The CDR sequences of these CDR variants of P06 are listed in Supplementary Table S5. (**C**) The amino acid type preferences for the HA-binding CDR variants of P06 are shown as sequence LOGO (Supplemental Methods) for each of the CDR positions (x-axis) calculated with the CDR sequences of the CDR variants of P06 in Supplementary Table S5. (**D**) The antibody P06 VL (colored in grey) and VH (colored in black) variable domain structures were computationally modelled with RosettaAntibody modeling software^63^ with default parameters. The four CDR positions highly conserved in aromatic amino acid types are highlighted in green for the aromatic carbon atoms.

In order to rule out the possibility that the germline antibody sequences or affinity maturation processes in nature could be the intrinsic factors leading the subsets of CDR aromatic residues to be the predominant determinant governing the specificity of the antibody–protein recognition, we experimentally investigated amino acid type preferences in the CDR positions of two artificial antibodies binding to proteins (M9-MSLN (human mesothelin) in Fig. 4 and P06-HA (influenza hemagglutinin) in Fig. 5. These two antibodies (M9 and P06) had been attained from the phage-displayed synthetic antibody libraries^55^ constructed with the scFv framework of human variable domain sequence combination of V_H_3–23–J_H_4 for the VH domain and Vκ1–Jκ1 for the VL domain with the CS combination of 1–2–2–1–1 shared by all the antibody structures analyzed in this work. The amino acid type preferences for each of the subset of the CDR positions (Figs. 4B and 5B) in M9-MSLN and P06-HA interfaces are shown in Figs. 4C and 5C respectively, which were attained following the same experimental procedure in attaining Figs. 2B and 3B ^48,62^. The results in Figs. 4C and 5C indicate that aromatic amino acid types are highly conserved in a few of the CDR positions in both M9-MSLN and P06-HA interfaces, and these CDR positions with strong aromatic amino acid type preferences are exposed to the surface of the antigen binding sites on the variable domains of M9 and P06 (Figs. 4D and 5D respectively). These results agree with the conclusions attained from Figs. 2 and 3, in that the antibody–protein interaction specificity is closely associated with a few of the CDR positions that are highly conserved for aromatic amino acid types to interact with the corresponding protein antigens. The MSLN-binding CDR variants of M9 and HA-binding CDR variants of P06 are artificially derived antibodies from phage-displayed synthetic antibody libraries without the limitations of the germline sequences and natural antibody affinity maturation, indicating that the dominance of the paratope-epitope interface specificity determinant by a subset of aromatic CDR residues is a generalizable phenomenon, likely driven by energetic principles governing antibody–protein recognitions.

## Discussion

With the computational antibody–protein complex analyses in the presence of interfacial waters and experimental investigations identifying indispensable CDR residues for specific antibody–protein complexes from enumerated CDR variants, we concluded that the indispensable interactions dictating the epitope specificity of the natural antibodies are mainly involved with the antibodies’ CDR aromatic sidechains. We found that antibodies recognize cognate protein antigens by forming key contacts involving a subset of aromatic CDR residue sidechains on the exposed CDR residue positions, and in addition, direct and water-mediated hydrogen bonds involving polar CDR residues occupying another set of CDR residue positions in the partially solvated interfaces bridging the CDRs and the cognate protein antigens^15,16,65,66^. These antibody–protein interfaces are not necessarily optimally complementary in geometrical shape as in the interfaces of the permanent protein–protein interactions. The interfacial direct/water-mediated hydrogen bonds could stabilize the complex interfaces but without substantial energetic driving force to determine epitope location, because the donors and acceptors involving direct/water-mediated hydrogen bonds in the partially solvated interface are exposed to the aqueous environment, and hence are frequently freely exchangeable with those from solvation water molecules^22,42^ and thus are not specific enough to select epitope location. Moreover, water-mediated hydrogen bonds are more extensive than direct hydrogen bonds in the antibody–protein complex interfaces, and the statistical overlapping of the CDR positions suitable for direct hydrogen bonds and water mediated hydrogen bonds suggests that these two types of interfacial polar interactions are interchangeable. Hence, as observed in Figs. 2 and 3, few polar CDR residues are highly indispensable for the corresponding interfacial structures. By contrast, the stereospecific features of the interactions involving aromatic CDR residues to protein backbone atoms and sidechain carbons have been well-established^2^, as demonstrated experimentally in the guest–host systems in aqueous environment^26,27,40^. Evidently, the specific interactions involving a subset of aromatic CDR residues surrounded with interfacial polar interactions largely form the predominant determinant underlying the specificity and affinity of the stereospecific antibody–protein complexes in nature.

These antibody–protein recognition principles described in the previous paragraph provide sensible insights into the antibody–protein complex formation. These principles explain that the antibodies from an antibody repertoire with limited sequence and structural diversity can recognize almost unlimited average protein antigen surfaces intermixed with roughly half hydrophobic and half hydrophilic residues^66^, where desolvation of the ubiquitous hydrophilic interfacial groups is prohibitive^33^ against formation of contiguously tightly packed interface as the predominant determinant for the specificity of paratope-epitope complexes. Although the protein sequences space in nature could be unlimited, aromatic sidechain binding sites composed of backbone atoms and sidechain carbons are common features ubiquitously shared on all protein surfaces^2^, such that the diversity space of the epitopes on protein antigens mapped by the aromatic sidechain binding propensity and electrostatic complementarity are vastly smaller in comparison with the protein sequence space of the epitopes characterized by amino acid type. Our findings explain why limited amino acid types encoded in the CDRs of natural antibodies can recognize almost unlimited protein antigen sequences. Similarly, although protein surface shape space in nature could be unlimited, aromatic sidechain contacts on the protein antigens do not necessitate large complimentary and continuous interfaces between the combining antibody-antigen pairs. The findings explain why the limited canonical CDR structures in the CDRH1–H2–L1–L2–L3 of natural antibodies^54^ do not impose limitation on the antibodies from recognizing a large variety of protein surface shapes. From these perspectives, it can be envisaged that antibodies with limited sequence and structural variations in an antibody repertoire are capable of recognizing seemly unlimited protein antigens as observed in nature—a phenomenon underlain by the aromatic and polar amino acids encoded in natural antibody CDRs.

## Methods

The computational methodologies related to ISMBLab-H2O have been published by our lab^48^ and are described in detail in Supplemental Methods. The LOGO calculation (see Supplemental Methods) and all the experimental methodologies have been published by our lab^48,55,64,67^ and have been used in this work without modification. Other technical details mentioned in this work are described below.

### ISMBLab-H2O prediction of water molecule placement around protein surfaces

The ISMBLab-H2O water placement prediction algorithm places the first water molecule at the grid position with the highest probability of water oxygen in the water oxygen probability density map (PDM) around the query protein structure. The construction of the PDM has been published previously^48^ and the detailed method is shown in Supplemental Methods. If the water molecule assigned to the initial position clashes with the query protein in van der Waals (vdW) volume, the center of the water oxygen moves in the direction away from the nearest protein atom to eliminate the clash; this step repeats until the water molecule no longer clashes with the query protein. If the clash cannot be resolved in less or equal to 10 cycles of moving the water molecule away from the query protein, the water molecule placement is abolished and the procedure return to the cycle of placing the next water molecules. Once the water molecule is placed, grid positions within the radius of water vdW volume (vdW(H_2_O) = 1.4 Å, Supplementary Table S3) are removed. The algorithm repeats the cycle of assigning a water molecule at the position of highest PDM value among the remaining grid positions and removing grid positions within the radius of the predicted water molecule until no further water molecule can be assigned around the query protein structure. Supplementary Fig. S1 shows the exemplary ISMBLab-H2O predictions of water molecule placements around the 20 natural amino acid types, and Supplementary Fig. S2A shows the exemplary ISMBLab-H2O predictions of water molecule placements around the antibody–protein complex G6-VEGF.

### META and RANDOM predictions of water molecule placement around protein surfaces

The META water molecule placement prediction in Table 1B was carried out by pooling together all predicted water molecule placements from the 4 prediction algorithms (Dowser++^49^, Fold-X^50^, 3D-RISM^51^ and ISMBLab-H2O), removing redundant predicted water molecules by the criterion of water center-to-center distance ≤ 2 × vdW(H_2_O) + 0.5 Å following the order of ISMBLab-H2O > Dowser++ > 3D-RISM > Fold-X in decreasing priority of surviving the removal of redundant predicted water molecules. Supplementary Fig. S2A–E show the exemplary predictions of water molecule placements around the antibody–protein complex G6-VEGF with these 5 water molecule placement prediction algorithms.

Three sets of RANDOM predictions (RANDOM (1–III) in Table 1B) were carried out by randomly assigning non-overlapping (center-to-center distance ≥ 2 × vdW(H_2_O) + 0.5 Å) water molecule placements within the interfacial spaces of the protein complexes until water placement saturation. The interfacial space in a protein-protein interaction complex is defined as the intersection space from the volumes of the first solvation layer of the interacting proteins, where the volume of the first solvation layer of a protein is the volume in the corresponding solvent accessible surface defined by a probe of radius = vdW(H_2_O) + 0.5 Å minus the van der Waals volume of the protein; vdW(H_2_O) = 1.4 Å is the van der Waals radius of a water molecule used throughout this work.

### Water-mediated hydrogen bonding (WMHB) and direct hydrogen bonding (DHB) in proteins

Both direct hydrogen bond (DHB) and water-mediated hydrogen bond (WMHB) were defined with VMD (http://www.ks.uiuc.edu/Research/vmd/) hydrogen bonding donor (D) and acceptor (A) criteria for both protein main-chain and side-chain: distance cutoff (D-A) ≤ 3.5 Å and angle cutoff (D-H-A) ≥ 150°. Psfgen (http://www.ks.uiuc.edu/Research/vmd/plugins/psfgen/) and CHARMM (http://mackerell.umaryland.edu/charmm_ff.shtml) were used to add hydrogen to protein structure. Some side-chain donors (Arg NH2, Lys NZ, Ser OG, Thr OG1, Asn ND2, Gln NE2, Tyr OH) are flexible in torsion angle rotation, such that for these donors, the angle cutoff was turned off with an additional constraint that a hydrogen must exist between the donor and acceptor. For defining water-mediated hydrogen bonds, the water molecule was treated as a rotating rigid body. For each of the three rotational degree of freedoms of the water molecule, the grid system of 36° interval was used to reduce the infinite conformational space to 500 grid points (10 × 10 × 5), which were enumerated for hydrogen bonding to the hydrogen bond donors/acceptors on the nearby proteins. Hydrogen bonding interaction mediated by an interface water molecule between the donor/acceptor from two separate proteins is defined as water-mediated hydrogen bonding (WMHB) if the interface water molecule is capable of forming hydrogen bonding with the two proteins. Supplementary Fig. S2G and H show examples of DHB and WMHB in the interface of the G6-VEGF complex.

### Atomistic contact pairs (ACPs)

The atomistic contact pair (ACP) of atom x in antibody A and atom y in protein antigen B in the AB complex forms when the center-to-center distant between x and y is less than vdW(x) + vdW(y) + 0.5 Å, where vdW(x) is the van der Waals radius of protein atom x. Supplementary Table S3 shows the list of van der Waals radius of protein atoms used in this work. Supplementary Fig. S2F shows examples of carbon-carbon ACPs in the interface of the G6-VEGF complex.

### S88 and S880 datasets

The S88 dataset contains the antibody–protein complexes from PDB (Supplementary Table S1) where the antibodies are limited in canonical structure (CS) for CDRH1–H2–L1–L2–L3 to the combination of CS type 1–2–2–1–1^54^ with the CDR sequence length of 13–10–11–8–9^55,56^. These complex structures were collected based on the following sequential criteria: (1) the initial list of antibody–protein complex downloaded from the structural antibody database (http://opig.stats.ox.ac.uk/webapps/newsabdab/sabdab/); (2) complex structures with protein antigen < 35 amino acids removed from the list; (3) antibodies with CDR length of H1, H2, L1, L2, and L3 respectively equal to 13, 10, 11, 8, and 9^56^ were selected; (4) removal of complexes with incomplete structure in any of the CDRs in the antibody variable domains; (5) complexes with CDR canonical structure combination of 1–2–2–1–1 for CDRH1–H2–L1–L2–L3 confirmed were selected; (6) manual selection of antibody–protein complexes with conformational epitope (epitope on the protein antigen composed with more than one discontinuous peptide segments); (7) clustering heavy chain amino acid sequences of the antibody variable domains using CD-Hit (https://github.com/weizhongli/cdhit) with sequence identity cutoff 95%, and constructing the non-redundant antibody–protein complexes list from the center of each of the antibody clusters.

S880 contains artificially generated antibody–protein complex structures derived by docking only the native paratope of an antibody with PatchDock^57^ to the non-native epitope surface of its corresponding protein from each of the antibody–protein pairs in the S88 dataset; that is, only the native paratope residues and the non-native epitope residues, where the residues in the native paratope-epitope are defined by the native antibody–protein complex in S88, were assigned as the PatchDock input for docking interfaces. Top 10 of the artificially docked complex structures, for which the paratopes were centered around the native paratopes and the epitopes were not overlapped with the native epitope on the counterpart protein antigen, were ranked by the geometrical complementarity with PatchDock and selected for each of the parent complexes in the S88 dataset to form the S880 dataset with 880 artificially generated complex structures.

The structures of the antibody and antigen for each of the modelled complex structures in S880 remain identical to those in their parent complex structures attained from PDB. That is, the PatchDock performed rigid body docking for each of the antibody-antigen pairs in S88 for artificial complex structures with non-native but optimal geometrical complementarity interfaces. Although docking of the antibody-antigen pairs with flexible protein structures could further improve geometrical complementarity in the interfaces, the prediction accuracy of the conformational changes during forming artificial complex structures is not possible to be validated. In addition, docking with flexible protein structures would consume exponentially increasing computational resources depending on the conformational space to explore. With the large number of complex structures for modelling in this work, we opted for the rigid body docking with PatchDock to limit the computational work to realistically accessible computational resources, with the understanding that the geometrical complementarity of the interfaces of the modelled complex structure from rigid body docking could be the lower limit of the interface matching. Arguably, the construction of S880 dataset with rigid body docking as a first approximation of a null hypothesis to compare with the S88 dataset described above fits the purpose of this study.

### Assessment of the uncertainty of computationally modeled structures on antibody–protein contact analyses of modelled antibody–protein complexes

Complex structures of the 244 CDR sequence variants shown in Supplementary Table 1A–E in Yu et al.^48^ were modelled with default Fold-X structural modeling algorithm^59^ based on the G6(Ab)–VEGF(Ag) complex structure in PDB code:2FJG^60^. Each of the 244 CDR variants was different from the parent antibody G6 in the amino acid sequence of only one CDR with sequence variation of no more than 5 residues (see the residue positions in Fig. 2); the other 5 CDRs remained the same as in the parent antibody G6. Only the sidechains of the CDR sequence variations were computational modelled with Fold-X—the VEGF structure and the G6 structure excluding the sidechain structures of the computationally mutated CDR residues remained the same as in the experimental structure 2FJG. These computationally modelled complex structures formed the dataset S244_2FJG_ as the positive control group.

We compared the modelled structures in S2442_FJG_ with another set of 1000 modelled G6-VEGF complex structures, for which the CDR sequence variants were generated with amino acid type distribution probabilities (q_ji_ in Eq. (3) in Supplemental Methods) used for the LOGO calculation (Fig. 2B). Like the construction of dataset S244_2FJG_ with VEGF structure and G6 structure excluding the computationally mutated sidechains unchanged, only the sidechains of the variant amino acids different from G6 were computationally modelled with Fold-X to form the dataset S1000(q_ji_)_2FJG_ as the comparable group for the positive control group S244_2FJG_. In difference, each of the variants in S1000(q_ji_)_2FJG_ had randomly picked amino acid type based on the distribution probability q_ji_ in all 30 residue positions of the 6 CDRs. As such, the variants in S1000(q_ji_)_2FJG_ were different from antibody G6 in 30 CDR residue positions distributed in all 6 CDRs at most, while the variants in S244_2FJG_ were different from antibody G6 in 5 CDR residue positions at most distributed in only 1 CDR.

To assess the impact of the uncertainty of the computationally modelled structures towards the analysis results of the interacting contacts in the antibody-antigen interfaces of the modelled complex structures, we compared the analysis results with the two datasets side-by-side. If no difference of the statistical analyses were observed, the impact of the structural modelling uncertainty would be deemed as insignificant in terms of interpretating the statistical analyses. We calculated the percentage of the antibody aromatic residue (FWY) at each of the CDR positions involving aACPs with VEGF in S244_2FJG_ dataset (Supplementary Fig. S5D) and compared the percentages with those calculated with the complex structures in S1000(q_ji_)_2FJG_ dataset (Supplementary Fig. S5E). We calculated the percentages of amino acid types involving naC-C ACPs with S244_2FJG_ dataset (Supplementary Fig. S5G) and compared the percentages with those calculated with S1000(q_ji_)_2FJG_ dataset (Supplementary Fig. S5H). We calculated the percentages of amino acid types involving DHBs and WMHBs with S244_2FJG_ dataset (Supplementary Fig. S5J and M) and compared the percentages with those calculated with S1000(q_ji_)_2FJG_ dataset (Supplementary Fig. S5K and N) respectively. The highly correlated side-by-side profile pairs (D and E, G and H, J and K, M and N for R^2^ = 1.0, 0.96, 0.97, 0.85 respectively) indicate that the modelling uncertainties associated with the computational complex structures in S244_2FJG_ and S1000(q_ji_)_2FJG_ datasets are not expected to be significant for the quantitative conclusions from the amino acid sequence profiles calculated based on the computationally modelled structures.

## Supplementary Information

Supplementary Information.

## Data Availability

ISMBLab-H2O and the datasets associated with the manuscript are available on the https://ismblab.genomics.sinica.edu.tw (http://140.109.55.4/).

## Abbreviations

Ab: Antibody
Ag: Antigen
ACP: Atomistic contact pair
aACP: Aromatic atomistic contact pair
C–C ACP: Carbon–carbon atomistic contact pair
naC-C ACP: Non-aromatic carbon–carbon atomistic contact pair
naC-P ACP: Non-aromatic carbon–polar atomistic contact pair
D-D/A-A ACP: Hydrogen bond donor–donor or acceptor-acceptor atomistic contact pair
CDR: Complementarity determining region
CS: Canonical structure
DHB: Direct hydrogen bond
IIA: Interfacial interaction atom
WMHB: Water-mediated hydrogen bond
VEGF: Vascular endothelial growth factor
HA: Influenza hemagglutinin
MSLN: Mesothelin
