# Supplementary material for: Antibody CDR amino acids underlying the functionality of antibody repertoires in recognizing diverse protein antigens
Source: Sci Rep. 2022 Jul 22;12:12555. doi: 10.1038/s41598-022-16841-9 (PMC9307644; doi:10.1038/s41598-022-16841-9)
Supplement: Supplementary file 1 — Supplementary Information. [file 41598_2022_16841_MOESM1_ESM.doc]

Supplemental Material

Title

**Antibody CDR amino acids underlying the functionality of antibody repertoires in recognizing diverse protein antigens**

Authors and Affiliations:

Hung-Pin Peng^a^, Hung-Ju Hsu^b^, Chung-Ming Yu^b^, Fei-Hung Hung^a^, Chao-Ping Tung^b^, Yu-Chuan Huang^a^, Chi-Yung Chen^b^, Pei-Hsun Tsai^a^, and An-Suei Yang^a,b,1^

^a^ Biomedical Translation Research Center, Academia Sinica, Taipei, Taiwan 155.

^b^ Genomics Research Center, Academia Sinica, Taipei, Taiwan 115.

Contact:

^1^ corresponding author: An-Suei Yang, Genomics Research Center, Academia Sinica, 128 Academia Rd., Sec.2, Nankang Dist., Taipei, Taiwan 115. Phone: +8862-2-2787-1232 email: [yangas@gate.sinica.edu.tw](mailto:yangas@gate.sinica.edu.tw)

Running Title: determinants in antibody-protein interactions

Keywords: antibody-protein interaction, complementarity determining region, phage-display synthetic antibody library, specificity of antibody binding, aromatic interactions, hydrogen bonding

**Table of Contents**

**Supplemental Methods**

1. Amino acid conformation clustering

2. Protein atomistic non-covalent interacting database

3. Predicting probability density maps (PDM) of non-covalent interacting atoms for protein surfaces

4. Pairwise amino acid type contact preferences in antibody (Ab)-protein antigen (Ag) interaction interfaces

5. Computation of the LOGO plots

**Supplemental Data**

**Supplementary Figure S1, related to Table 1.** Water molecule placement predictions around all 20 natural amino acids.

**Supplementary Figure S2, related to Table 1 and Figure 1.** Water molecule placement predictions on the antibody-protein complex (G6-VEGF).

**Supplementary Figure S3, related to main text.** Distributions of interfacial interacting atoms (IIAs) versus predicted interfacial waters around the IIAs in the interfaces of the antibody (Ab)-protein antigen (Ag) complexes in S88 and S880 datasets.

**Supplementary Figure S4, related to main text.** Pairwise amino acid type contact preferences in antibody-protein interaction interfaces of the antibody-protein complexes in S88 dataset in comparison with those in S880 dataset.

**Supplementary Figure S5, related to Figures 2 and 3.** Assessment of computationally modeled structures.

**Supplementary Table S1, related to Figure 1.** List of 88 non-redundant 1-2-2-1-1 antibody-protein complex PDB code and the sequences in the CDRs of the antibodies.

**Supplementary Table S2, related to Supplementary Figure S1.** Amino acid conformation classifications.

**Supplementary Table S3, related to Figure 1**. Atom types and van der Waals radius in protein structures.

**Supplementary Table S4, related to Figure 4.** CDR variant sequences of M9.

**Supplementary Table S5, related to Figure 5.** CDR variant sequences of P06.

**Supplemental References**

**Supplemental Methods**

***1. Amino acid conformation clustering***

Amino acids in proteins are limited in structural diversity. Protein structures are determined by mainchain and sidechain torsion angles of the constituent amino acids. The distributions of the torsion angles are clustered around prevalent conformational centers, instead of spreading continuously over the torsion angle space. The mainchain torsion angles are clustered at the α- and the β-regions in the Ramachandran plot; the distributions of the sidechain torsion angles are also concentrated on only a few allowable regions, depending on the chemical constituents of the sidechain ^1,2^. Moreover, the distribution of each of the sidechain torsion angles is dependent on the torsion angles of the backbone of the amino acids ^3^. Thus, amino acid conformations in proteins can be organized into limited sets of clusters based on the mainchain and sidechain torsion angle set of each of the amino acid types, allowing interacting atom pair database retaining conformational information of the parent amino acids.

Database for non-covalent interacting atom pairs in proteins was organized according to parent amino acid conformational types. To cluster amino acid conformations into a limited set of clusters for each type of amino acid, we assigned torsion angles to each of the amino acids in known protein structures with the computer program DSSP ^4^ and MOLEMAN 2 ^5^. For each type of amino acid from the protein structure entries in PDB, a set of vectors with torsion angle elements in degree ({*φ*, *ψ*, χ*_1_*, …, χ*_i_*}, where *φ*, *ψ* are backbone torsion angles and χ*_i_* are sidechain torsion angles as defined conventionally) was established; amino acid residues with incomplete structure were excluded from the data sets. The vectors were used as input to the fuzzy c-means algorithm ^6^ for clustering. The number of the clusters was determined as the minimal integer satisfying the condition that increasing the number of clusters beyond this minimal integer made little change to the partition index and separation index – two fuzzy c-means algorithm indexes describing the relative mean distance within and between clusters ^7^. To augment the optimal decision on cluster numbers, we calculated the distribution of the intra-cluster RMSD (root mean squared deviation) in Å for superimposed amino acid structures between cluster members and the centroid conformation within a cluster for each cluster sets. The convergence of this intra-cluster RMSD to a minimal RMSD provided a more structure-related reference in contrast to the torsion angle-based structural descriptors in determining the optimal cluster number. After the determination of the cluster numbers, the centroid conformation of each of the clusters was determined as the center of mass of the vectors in the cluster. The number of clusters, the torsion angles of the centroid conformations, and the distribution information of the members in the clusters are listed in Supplementary Table S2.

***2. Protein atomistic non-covalent interaction database***

Atomistic contact interactions in proteins of known structures were organized into a database containing non-covalent atomistic interaction information for atom pairs in protein structures. For each of the atoms in residue X of a protein, the non-covalent interacting atoms were recorded as the following: Following the work of Laskowski et al. ^8^, for each atom (P) in residue X, the relative location of the atom P was defined with two consecutive atoms R and Q, where R is covalently linked to P, and Q is covalently linked to R. Atom R was set at the origin of the reference coordinate system; atom P was located on the z-axis; atom Q was on the z-x plane of the reference coordination system. In principle, all non-covalent interacting atoms to atom P were recorded in the database with the reference coordination system. After the survey on all the non-covalent interacting atom pairs, the database was organized into a large number of files; each file is specific to an amino acid type, a conformational type based on the torsion angle vector of the amino acid, an atom type in the parent amino acid, and the interacting atom type. The structure of the data files facilitates the speedy random access of the database in predicting distribution of probability density maps (PDM) of non-covalent interacting atoms.

Water oxygen distributions around the surface amino acids in 915 non-redundant protein structures solved to high resolution (resolution<1.5Å, sequence identity less than 30%, different graph topology and subunit structure) ^9^ were recorded with the same P-R-Q reference coordination system and were stored in the file system as described above. Water oxygens within 3.2 Å radius (within hydrogen bonding distance) to the interacting amino acid atoms were recorded in the database. This database was used to generate the PDM of water around protein atoms (see below), examples of the PDMs are shown in Supplementary Figure S1.

***3. Predicting probability density maps (PDM) of non-covalent interacting atoms for protein surfaces***

A probability density map (PDM) of a non-covalent interacting atom type is a three-dimensional distribution of likelihood for the type of atom to appear around protein surface amino acids. In this work, the PDMs were reconstructed from the interacting atom pair databases described in the previous section for water molecules on protein surfaces.

To construct a PDM for an interacting atom type on a target protein surface, the computer algorithm first enclosed the target protein structure in a rectangular box clearing the structure by a margin of at least 7 Å from all sides of the protein’s edge. The three-dimensional rectangular box was then gridded with 0.5 Å per unit in three-dimensional space. This grid size was a balance between the resolution of the PDM and the computational resources needed for the PDM construction. The grid points enclosed within the Connolly surface ^10^ of the target protein were masked from assigning PDM.

The torsion angles of sidechain and mainchain of all the amino acids in the protein structure were calculated with MOLMAN2 and DSSP respectively. For each of the amino acid residues in the protein, the conformational type of the amino acid X was determined by the torsion angle vector, which had the least Euclidean distance to the centroid conformation of the assigned conformational cluster. With the assignment of the conformational type for each of the amino acids in the protein structure, the non-covalent interacting atoms around each atom P in the protein structure were allocated from the database according to the atom type of P, the assigned three-atom reference system P-R-Q as described in the previous section, the amino acid type of the parent residue containing atom P, and the conformational type of the parent amino acid. Interacting atoms outside the sphere with the radius equal to the sum of the van der Waals radii of the interacting atom and atom P plus a tolerance of 0.5 Å were not included as the interacting atoms with atom P. The coordinates of the allocated interacting atoms were transformed to the coordination system of the protein structure and mapped around the protein surface. An atom of non-covalent interaction was to be mapped only once for which the distance of the atom to P was the shortest.

In order to keep PDMs high in information content and low in noise from irrelevant interactions, allocation of interacting atoms according to the amino acid conformational type (as described above) is crucial for retaining information content in PDMs. Alternative approach for PDM construction with interacting atoms allocated from mixed amino acid conformational types would lead to loss of fidelity in relative orientations of the interacting atoms, resulting in spreading PDMs around dihedral bonds. We found that mapping interacting atoms obtained from an atom in an amino acid conformational type onto the surroundings of the atom in another amino acid conformational type led to serious spatial distortion of the distribution of the interacting atoms.

PDMs were constructed by mapping the interacting atoms allocated from the database as described in the previous paragraphs to the 3D grid system. To construct the PDM, each of the interacting atoms was distributed to 8 nearest grid points; the portion of the distribution was normalized by the database redundancy and was inversely proportional to the square of the distance from the atom to the grid:

$v_{ji}=\frac{1}{p_{i}n}\frac{\frac{1}{d_{ji}^{2}}}{\sum_{k=1}^{8} \frac{1}{d_{ki}^{2}}}$ Eq. (1)

, where *v_ji_* is the value to be accumulated at a nearest grid point *j* for interacting atom *i*; *d_ji_* is the distance of grid point *j* to the center of the interacting atom *i*; grid points indexed *k*=1~8 are the nearest grids to the atom *i*; *n* is the number of residues collected in the database for the amino acid in the target protein with the conformational type defined by the torsion angle vector; *p_i_* is the background probability for atom type *i* to appear in all protein structures (when calculating water oxygen PDM, *p_i_* equals to 1). The factor 1/*n* in the Equation is to normalize the interacting atom density according to one conformation for each of the residues in the target protein and the background probability *p_i_* is to normalize the PDM based on the appearance frequency of the atom type *i* in proteins (except for water oxygen). The PDM for each of the interacting atom types was additively accumulated to completion as each of the atoms in the target protein surface finished contributing to the PDMs.

***4. Pairwise amino acid type contact preferences in antibody (Ab)-protein antigen (Ag) interaction interfaces***

In Ab-Ag complexes, pairwise amino acid type contacts are categorized into three contact groups: contact group 1 – the mainchain and sidechain carbons of amino acid type x in Ab contact the mainchain and sidechain carbon of amino acid type y in Ag through at least one carbon-carbon atomistic contact pair (C-C ACP); contact group 2 – the mainchain and sidechain hydrogen bond acceptor/donor of amino acid type x in Ab contacts the mainchain and sidechain hydrogen bond donor/acceptor of amino acid type y in Ag through at least one DHB; contact group 3 – the mainchain and sidechain hydrogen bond acceptor/donor of amino acid type x in Ab interacts with the mainchain and sidechain hydrogen bond donor/acceptor of amino acid type y in Ag through at least one WMHB. The pairwise amino acid type contact preferences for amino acid type x in Ab to interact with amino acid type y in the corresponding Ag in the Ab-Ag complexes in nature (complex structures in S88) are compared with those in the PatchDock-generated artificial Ab-Ag complexes in S880 based on the log-odd ratio equation below:

P_n_(x,y) = log_10_ [(C(n)^s88^_x-y_/Σ_z=20 AA_C(n)^s88^_x-z_)/ (C(n)^s880^_x-y_/Σ_z=20 AA_C(n)^s880^_x-z_)] Eq. (2)

, where P_n_(x,y) is the log-odd ratio pairwise amino acid type contact preference for amino acid type x in Ab interacting with amino acid type y in the corresponding Ag for contact group n (n=1~3) (see above) in the Ab-Ag complexes in S88 in comparison with those in S880; C(n)^s88^_x-y_ and C(n)^s880^_x-y_ are the number of contacts from the contact group n of Ab amino acid type x to Ag amino acid type y in the natural Ab-Ag complexes in S88 and in the PatchDock-generated artificial Ab-Ag complexes in S880, respectively.

***5. Computation of the LOGO plots***

The size *d_ji_* (in half-bit unit) for amino acid type *i* at position *j* in a sequence LOGO is calculated with the equation:

Eq. (3)

*C_ji_* is the count for amino acid *i* at position *j* in *M_j_* count of the CDR sequences containing position *j*; *p_i_* is the background probability for amino acid *i* encoded in the NNK degenerate codon ^11^; the square root of *M_j_* in the equation is the pseudo count to prevent singularity when *C_ji_* equals to zero. Equation (3) is modified after the original formulation ^12^.

**Supplemental Data**

**Supplementary Figure S1, related to Table 1.** Water molecule placement predictions around all 20 natural amino acids. The amino acid structures, as shown by the ball-and-stick models with colored atoms in red, white, grey, blue and yellow for oxygen, hydrogen, carbon, nitrogen, and sulfur respectively, are determined with the torsion angles in the mainchain and sidechain torsion angle library shown in Supplementary Table S2. The PDM threshold (see Supplemental Methods) of 0.001 was used to show the PDM of water distribution probabilities, as indicated by the grey contours in the panels of this figure. Predicted ISMBLab-H2O water molecule placements are shown in the panels as the red spheres on top of the grey contours.

**
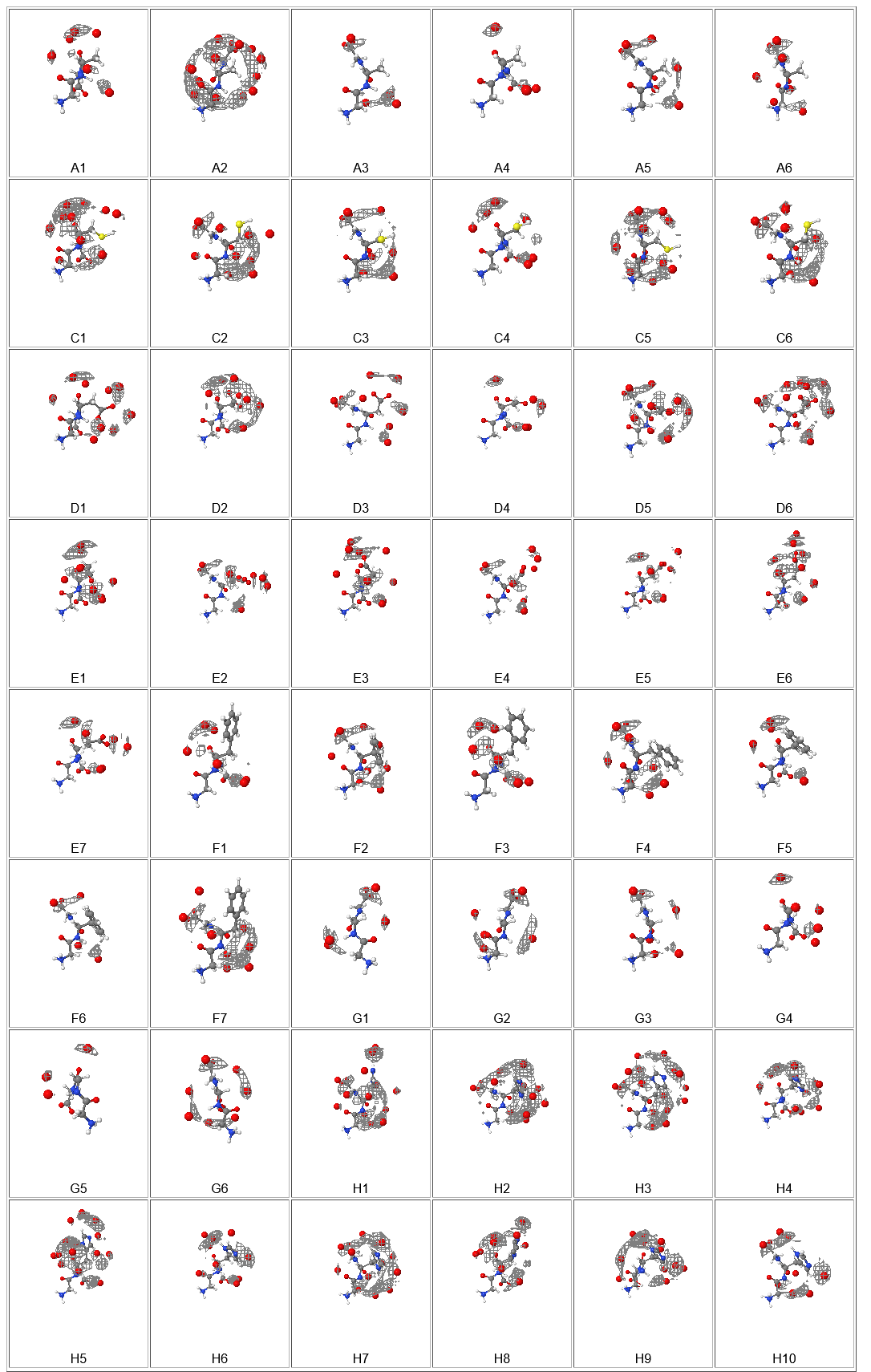
**

**
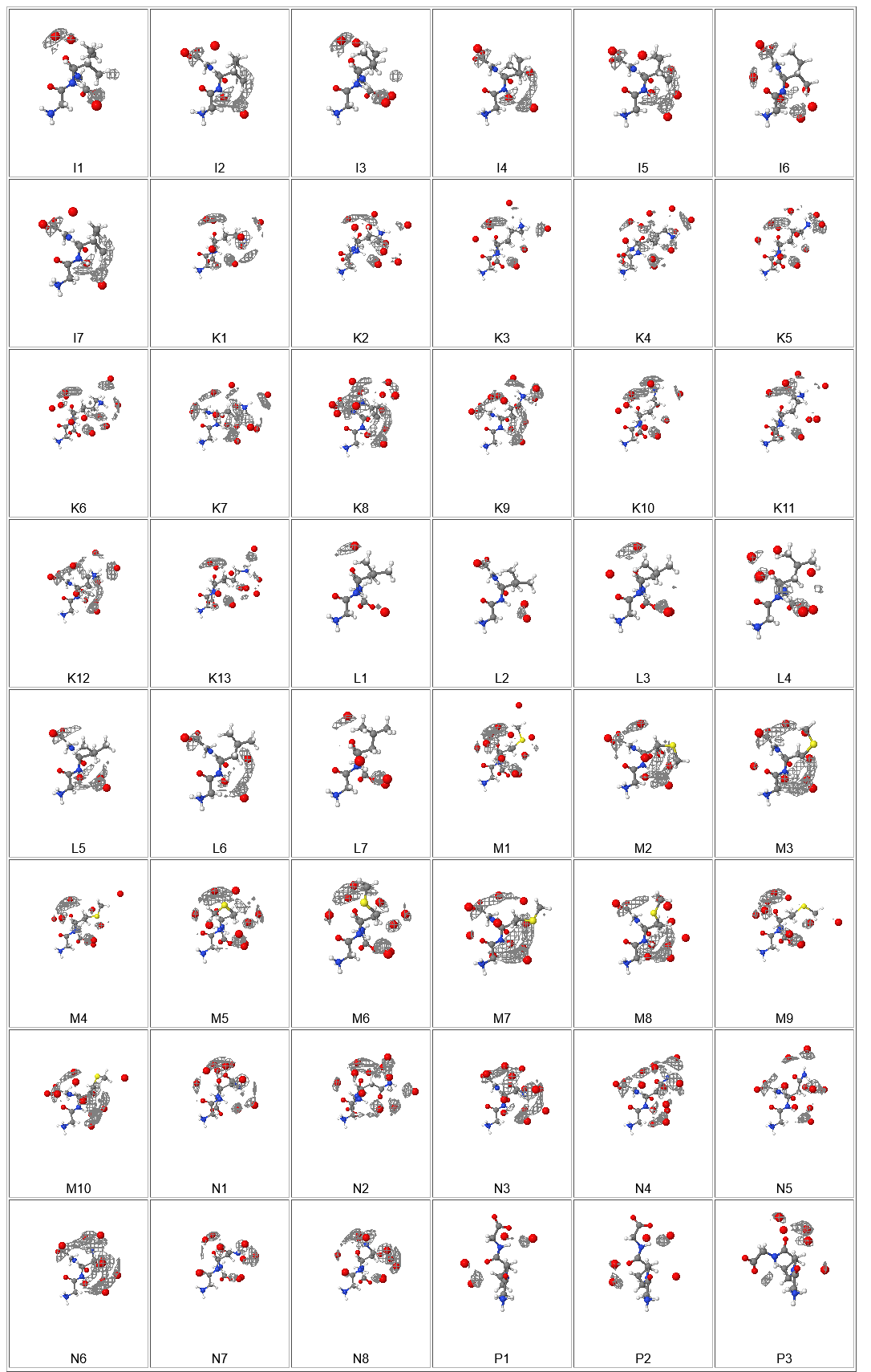
**

**
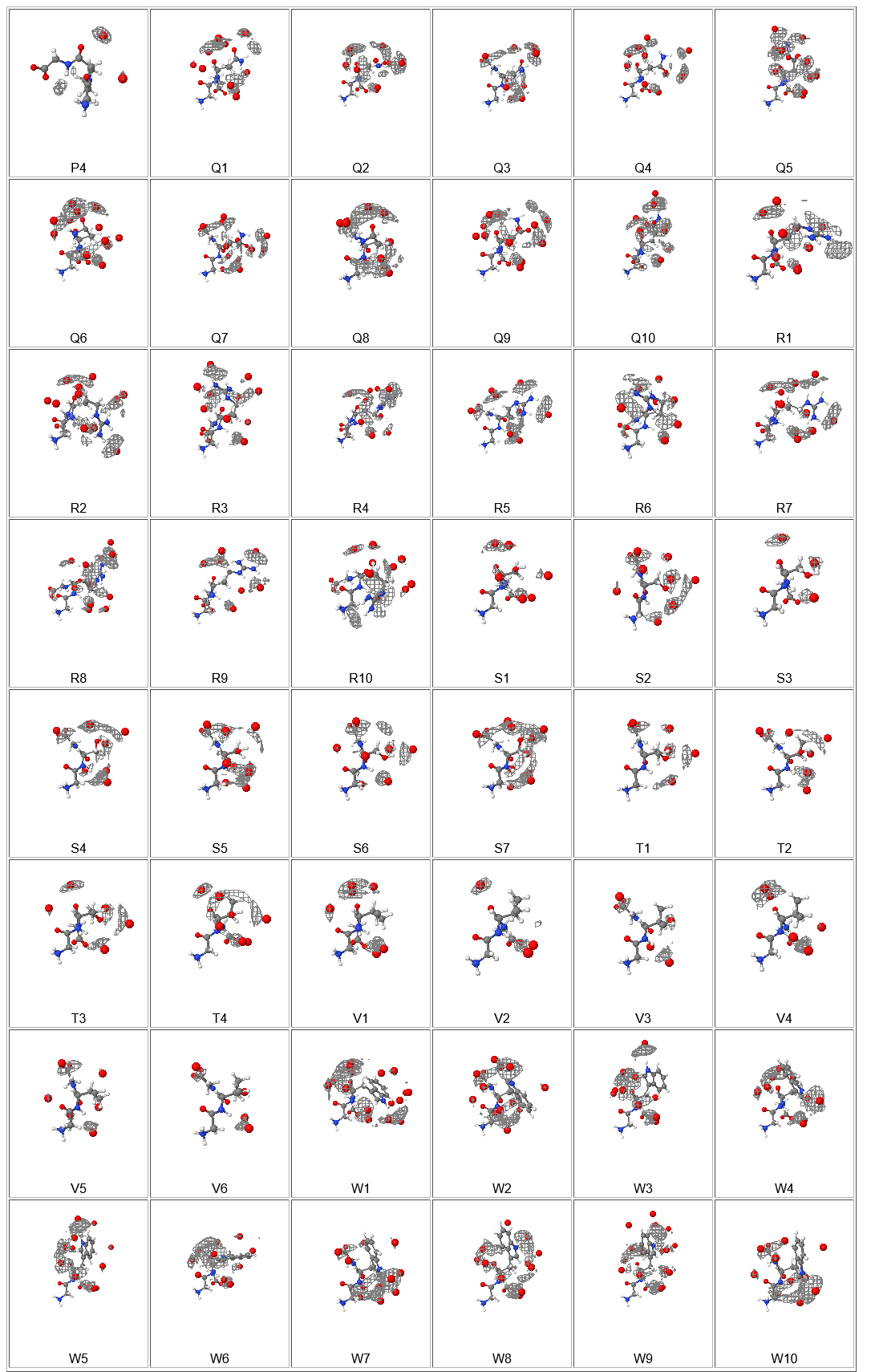
**

**
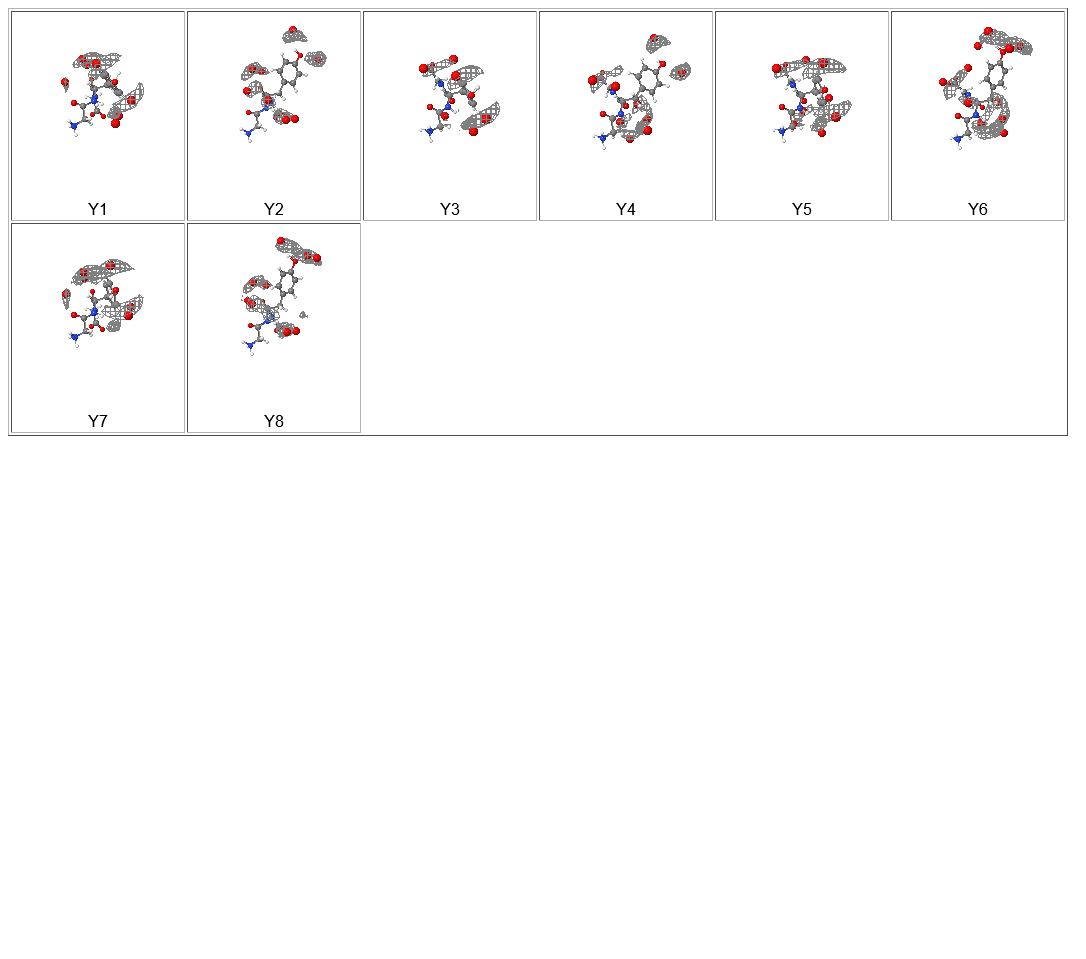
**

**Supplementary Figure S2, related to Table 1 and Figure 1.** Water molecule placement predictions on the antibody-protein complex (G6-VEGF) ^13^. The amino acid structures are shown by the stick models, and the predicted water structures are shown in red spheres. The carbons of the antibody G6 are colored in green and the carbons of the protein VEGF are colored in cyan. The prediction results with ISMBLab-H2O, Dowser++, 3D-RISM, Fold-X and META are shown in panels (A)~(E) respectively. In the antibody-protein complex interface, the carbon-carbon atomistic contact pairs are linked with white bars (panel F); the direct hydrogen bonding donor/acceptor pairs are linked with green bars (panel G); the water-mediated hydrogen bonding donor/acceptor pairs are linked in magenta bars (panel H).

**
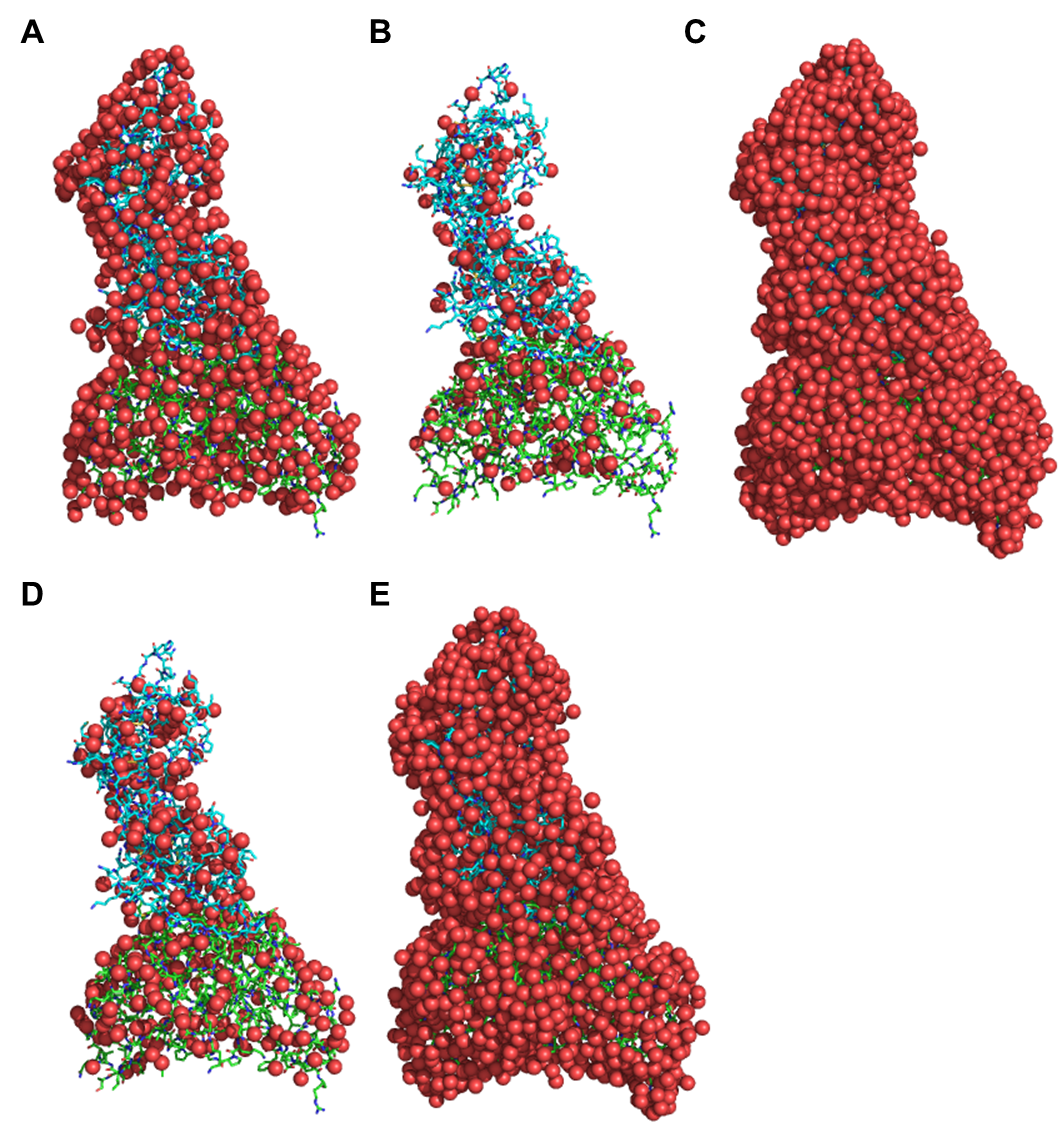
**

**
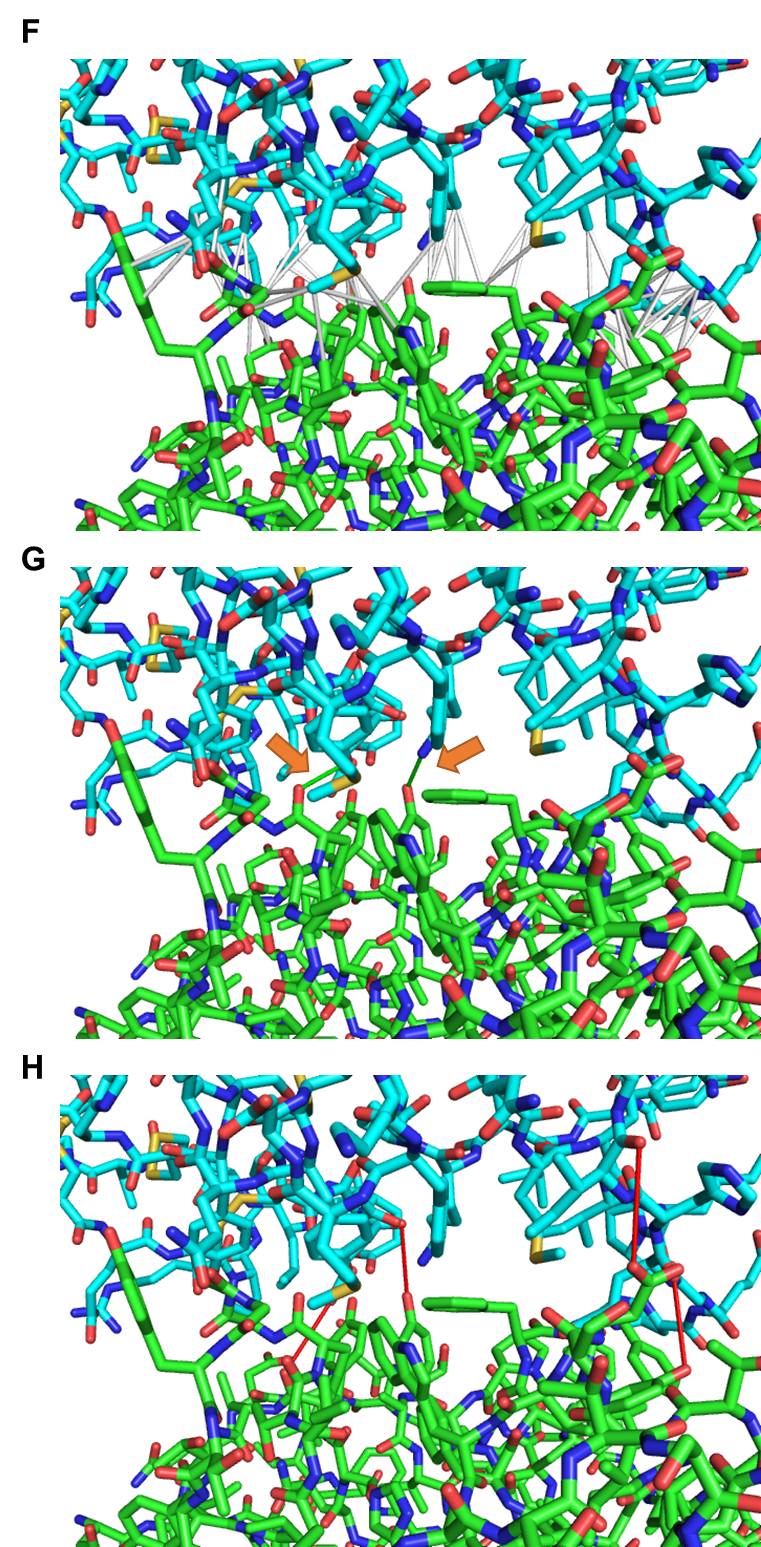
**

**Supplementary Figure S3, related to main text.** Distributions of interfacial interacting atoms (IIAs) versus predicted interfacial waters around the IIAs in the interfaces of the antibody (Ab)-protein antigen (Ag) complexes in S88 and S880 datasets. Water molecule placements on Ab and Ag in B form (bound Ab and Ag in a complex structure) and NB form (non-bound isolated Ab or Ag) were predicted with the META algorithm for each of the antibody-protein complexes in the S88 and S880 datasets (see Methods). Predicted interfacial waters around a protein interfacial interacting atom (IIA) x were assigned to the atom x by a water-x center-to-center distance threshold ≤ vdW(x)+vdW(H_2_O)+0.5Å, where vdW(x) is the van der Waals radius for the protein atom x (Supplementary Table S3); IIAs of Ab and Ag are the atoms involving in ACP, DHB, or WMHB in the Ab-Ag complex structure. (A) The curves are the distributions of number of IIAs per complex structure (y-axis) versus the number of predicted interfacial waters assigned to the IIAs (x-axis) for the Abs and Ags in B and NB form in the complex structures of S88 and S880 datasets. Panels (B)~(G) show the distributions of each respective subset of IIAs: The y-axis shows in (B) the numbers of IIAs involving aACPs; in (C) the numbers of IIAs involving naC-C ACPs; in (D) the numbers of IIAs involving DHBs; in (E) the numbers of IIAs involving WMHBs; in (F) the numbers of IIAs involving carbon-polar atomistic contact pairs (naC-P ACPs); in (G) the numbers of IIAs involving hydrogen bond donor-donor or acceptor-acceptor atomistic contact pairs (D-D/A-A ACPs). Data distributions in panel (A) are sums of the corresponding data distributions in panels (B)~(G). The datasets in (A)~(G) are plotted as the percentage out of total IIAs (y-axis) in the (H)~(N) panels respectively.


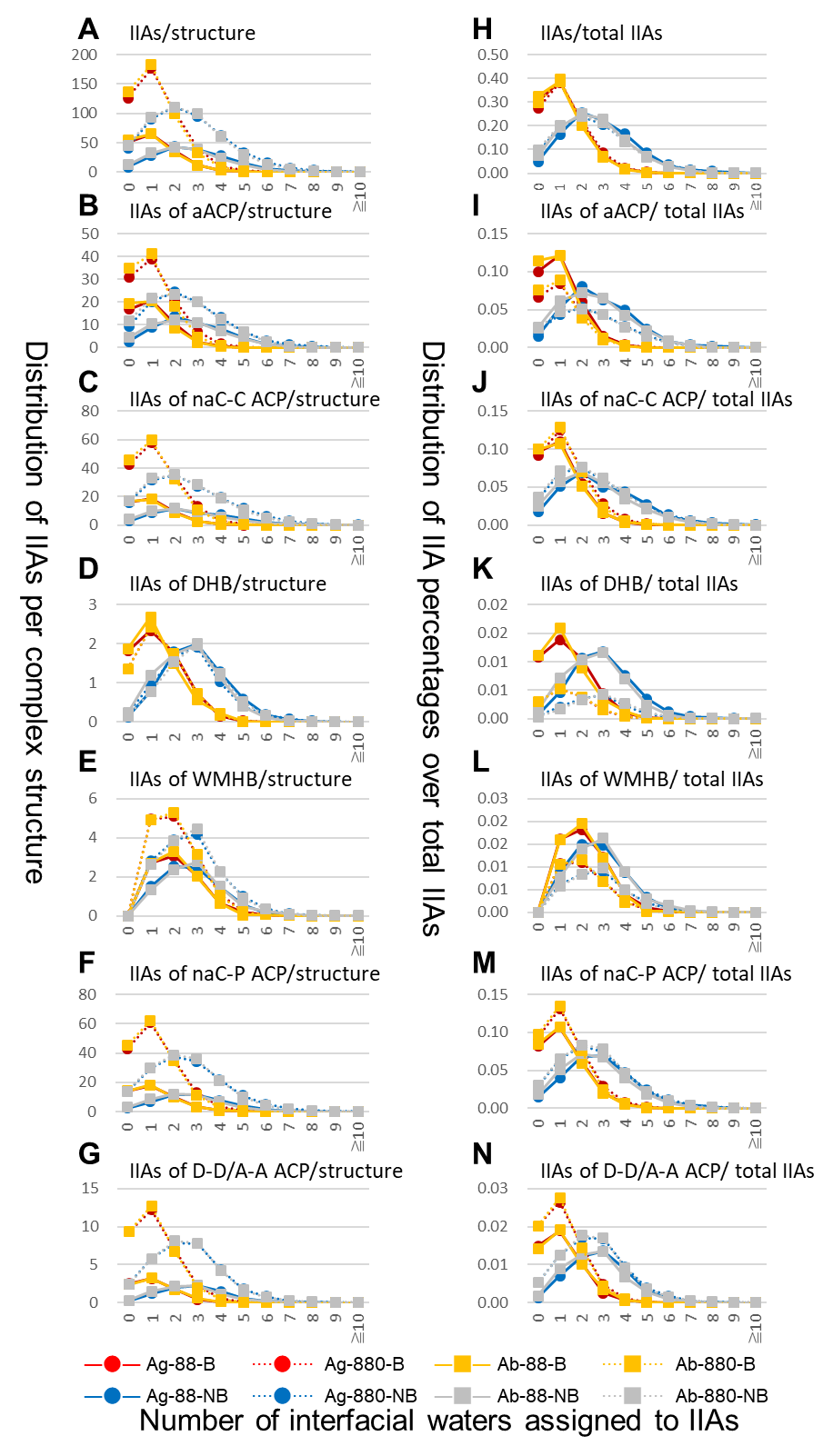


**Supplementary Figure S4, related to main text.** Pairwise amino acid type contact preferences in antibody-protein interaction interfaces of the antibody-protein complexes in S88 dataset in comparison with those in S880 dataset. The matrix of the figure shows the values of P_n_(x,y) in Eq. (2) in Supplemental Methods, which are the log-odd ratio pairwise amino acid type contact preferences for amino acid type x in Ab interacting with amino acid type y in the corresponding Ag for contact group n (n=1~3): x-y contact group 1 through C-C ACP, contact group 2 through DHB and contact group 3 through WMHB. When any of the terms in Eq. (2) in Supplemental Methods equals to zero, the corresponding P_n_(x,y) in the matrix is blank.

**
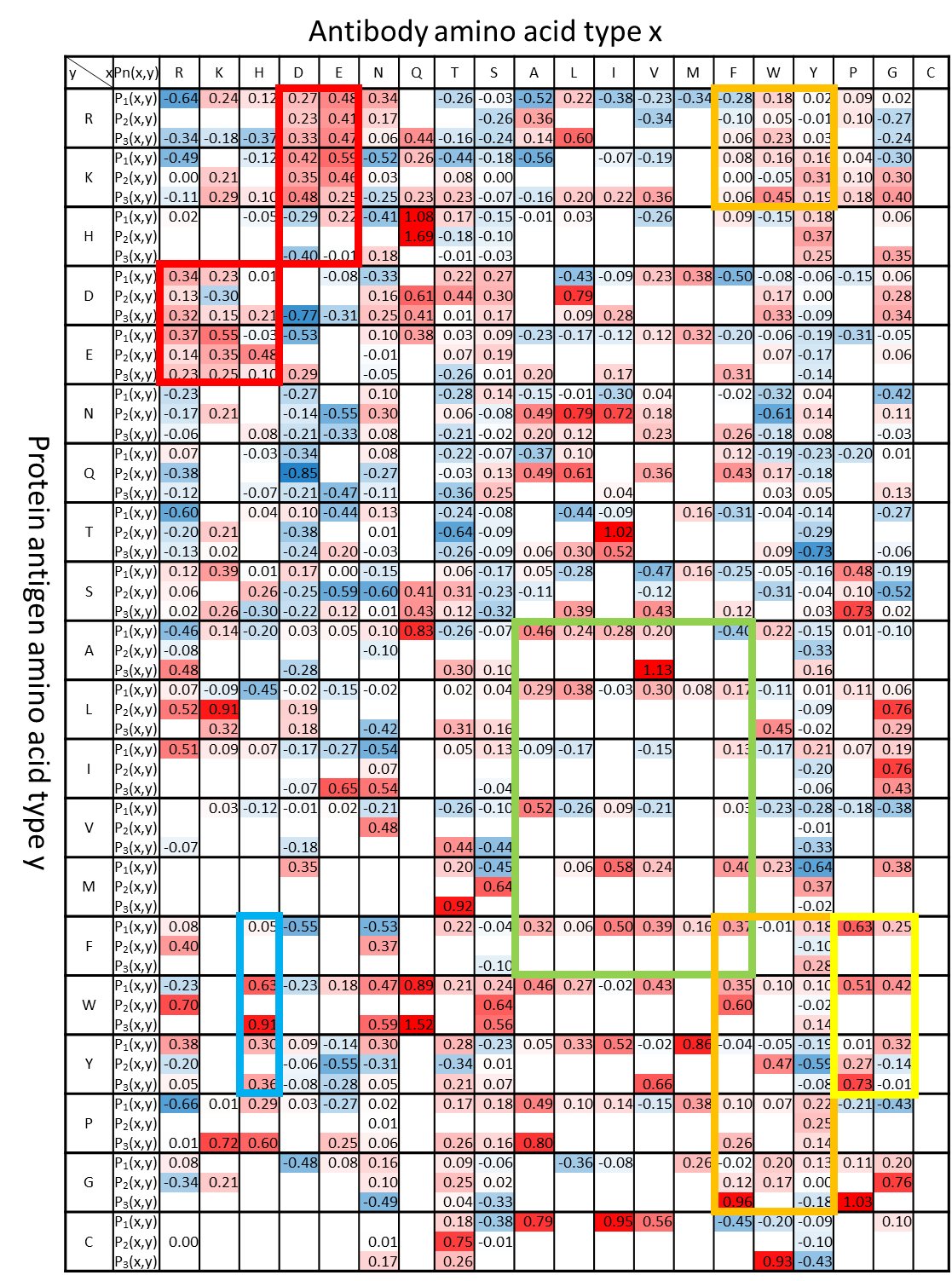
**

**Supplementary Figure S5, related to Figures 2 and 3.** Assessment of computationally modeled structures. Complex structures of the 244 CDR sequence variants shown in Supplementary Table 1(A)~(E) in Yu et al. ^14^ were modelled with default Fold-X structural modeling algorithm ^15^ based on the G6(Ab)-VEGF(Ag) complex structure in PDB code:2FJG ^13^. These computational structures form the dataset S244_2FJG_ as the positive control group. (A) The amino acid sequence of the G6 CDR residues are shown with the IMGT numbering in the x-axis. (B) The CDR sequences in S244_2FJG_ were used to calculate the antibody sequence LOGO (d_ji_ of Eq. (3) in Supplemental Methods) (y-axis) for the CDR positions (x-axis). The amino acid type distribution probabilities (q_ji_ in Eq. (3) in Supplemental Methods) used for the LOGO calculation were applied to generate 1000 CDR sequence variants for the computational complex structures built with Fold-X. These computational structures form the dataset S1000(q_ji_)_2FJG_ as the comparable group for the positive control group S244_2FJG_. (C) This panel shows the aromatic residue (FWY) positions and amino acid types in G6 forming aACPs with VEGF in the experimental complex structure. (D) The y-axis shows the percentage of the antibody aromatic residue (FWY) at each of the CDR positions (x-axis) involving aACPs with VEGF in S244_2FJG_ dataset; (E) The y-axis shows the percentage of the antibody aromatic residue (FWY) at each of the CDR positions (x-axis) involving aACPs with the VEGF in S1000(q_ji_)_2FJG_ dataset. (F)~(H) These panels follow the same description as in the panels in (C)~(E) for the percentages of amino acid types involving naC-C ACPs calculated with S244_2FJG_ dataset (panel G) and S1000(q_ji_)_2FJG_ dataset (panel H). Similarly, panels in (I)~(K) and panels in (L)~(N) show the percentages of amino acid types involving DHBs and WMHBs calculated with S244_2FJG_ dataset (panels J and M) and S1000(q_ji_)_2FJG_ dataset (panels K and N) respectively. The highly correlated sequence profile pairs (D and E, G and H, J and K, M and N for R^2^ = 1.0, 0.96, 0.97, 0.85 respectively) indicate that the modelling uncertainties associated with the computational complex structures in S244_2FJG_ and S1000(q_ji_)_2FJG_ datasets are within the tolerance for the quantitative conclusions from the amino acid sequence profiles calculated based on the computationally modelled structures.

**
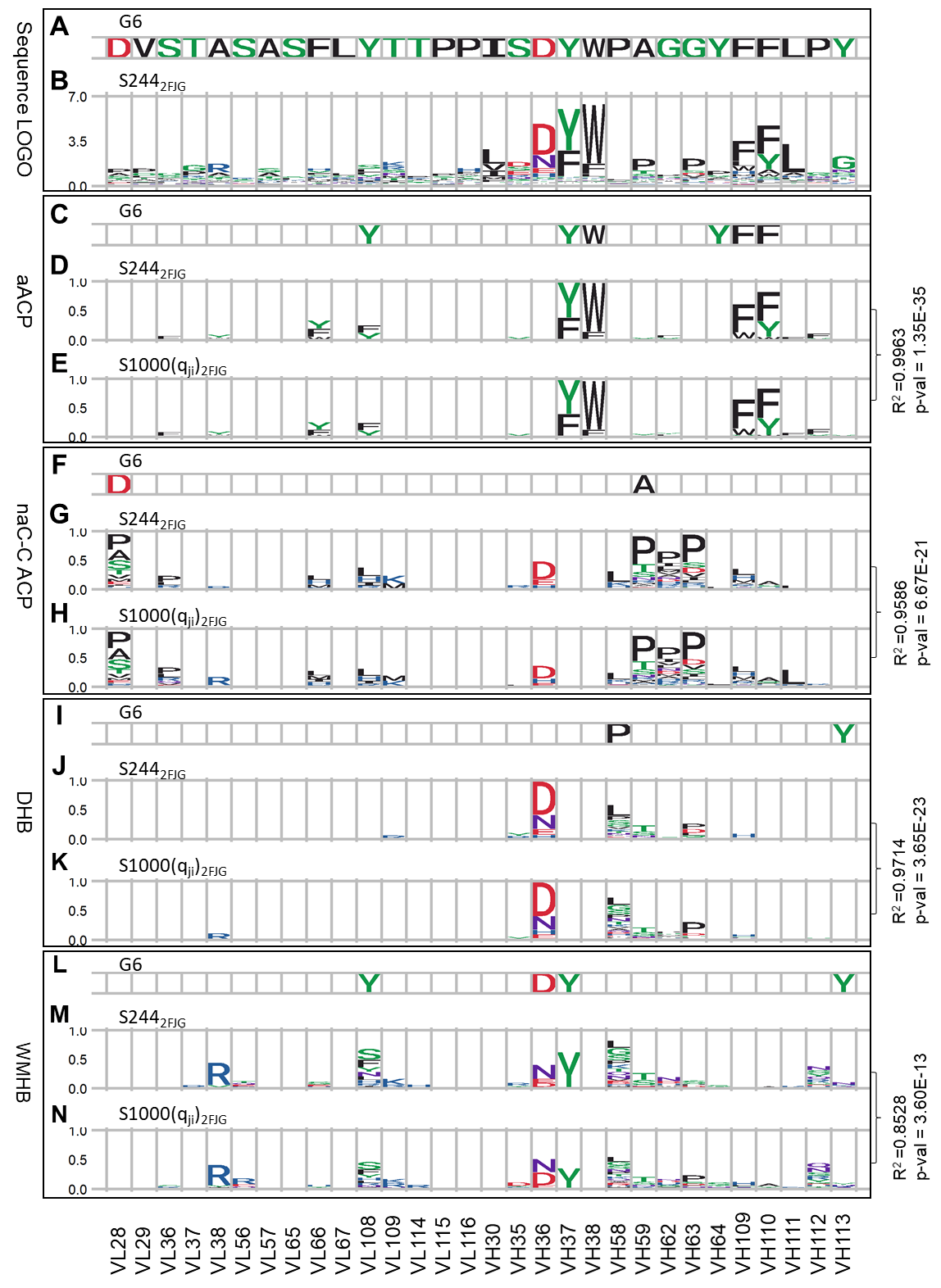
**

**Supplementary Table S1, related to Figure 1.** List of 88 non-redundant 1-2-2-1-1 antibody-protein complex PDB code and the sequences in the CDRs of the antibodies in the S88 dataset. The CDR residue ranges are marked in IMGT numbering in the first row.

| PDBID | L1(24-40) | L2(55-69) | L3(105-117) | H1(24-40) | H2(55-66) | H3(105-117) |
| --- | --- | --- | --- | --- | --- | --- |
| 3skj | RASQSISTWLA | YKASNLHT | QQYNSYSRT | AASGFTFSHYMMA | RIGPSGGPTH | AGYDSGYDYVAVAGPAEYFQH |
| 4ers | RASQGIRNDLG | YAASSLES | LQHNSNPLT | AASGFTFSSYGMH | VMYYDGSNKD | AREKDHYDILTGYNYYYGLDV |
| 4mwf | RASQSVSSNLA | YGASTRAT | QQYYRSPLT | ETSGGTFDNYALN | GVVPLFGTTR | VRSVTPRYCG-GGFCYGEFDY |
| 4jhw | QASQDIVNYLN | YVASNLET | QQYDNLPLT | QASGGPLRNYIIN | GIIPVLGTVH | ATETALVVST--TYLPHYFDN |
| 3sob | RASQDVSTAVA | YSASFLYS | QQSYTTPPT | AASGFTFTNSYIH | WITPYGGYTN | ARGSGHVNAV--KNYGYVMDY |
| 4n9g | RASQGISNYLA | YYTSHLES | QQHNSYPRT | VVSGGSFSSYYWT | EMNGNSGYTN | ARDAIVMVF---TDMRGRVDV |
| 4cad | RASGNIHNYLA | YNAKTLAD | QHFWSTPWT | KATGYKFSSYWIE | EIFPGSGNTN | ARRGAFYSY---GSSYYAMDF |
| 3vg9 | RASEFIYSSLT | YAATNLAD | QHFYGSTWA | KTSGDSFTAYNMN | NINPYYGSTR | AREGNYYDG----GSVRYFDY |
| 2qql | RASQDVSTAVA | YSASFLYS | QQAWAYLPT | AASGFTISGYGIH | YIYPDSGYTD | AREDFRNRR----RLWYVMDY |
| 3o0r | RASKSIRKYLA | YSGSTLQF | QQHNEYPLT | KASGYSFTSYWMH | AVYPGNSDTS | SRSSLDGYY----VKNWCFDV |
| 4g80 | RASQSVSSAVA | YSASSLYS | QQHQYNSLI | AASGFNVYYSSIH | YISPSSGSTY | ARKQYSYWR----DSYWAMDY |
| 2qqn | RASQYFSSYLA | YGASSRAS | QQYLGSPPT | AASGFTFSSYAMS | QISPAGGYTN | ARGELPYY-----RMSKVMDV |
| 3g6j | RASQDVSTAVA | YSASFLYS | QQSYATLPT | AASGFSFTSSSVS | LIYPYNGFNY | ARNALYGS-----GGYYAMDY |
| 4plj | RASENIYSNLV | YAATNLPD | QHFWETPFT | KASGYTFTDFNMH | YIYPYNGITG | ARERFGVG-----NNYAWFTY |
| 1qfw | KASETVDSFVS | FGASNRFS | GQTYNHPYT | AASGFAFSSFDMS | SITNVGTYTY | ARQGTAAQ------PYWYFDV |
| 4ki5 | RASQEISGYLS | YAASTLDS | LQYASYPYT | KATGYTFSSYWIE | EILPGSGSTN | TRTSYYFG------SSYDFDV |
| 3vrl | RASGNIHNYLA | YNAKTLAD | QHFWSTPRT | AASGFTFSSYTMS | IISSGGSYTY | TRDEGNGN------YVEAMDY |
| 3kr3 | RASQSISNYLN | YTASTLQS | QQSYNSPWT | AASGFTFSNYIMW | VISSSGGMTR | ARDNGDYV------GEKGFDI |
| 4qti | RASQDIGSSLN | YATSSLDS | LQYATSPYT | KAAGYTFTAYWIE | EILPGSSSTN | TRDFSGDR------SNLYFDV |
| 4f2m | RASQSIGTSIH | KYASESIS | QQTDSWPTT | KASGYAFSSSWMN | RIYPGDGETN | ARGGYRYD------PYYAMDY |
| 4dke | RASQDVSTAVA | YSASFLYS | QQSFYFPNT | AASGFTFSSTWIH | RISPYYYYSD | ARGLGKGS------KRGAMDY |
| 2r56 | RASQGISSRLA | YAASSLQS | QQYHSYPWT | TASGFTFRHHGMT | SLSGSGTKTH | AKAKRVG-------ATGYFDL |
| 3p0y | RASQDLATDVA | YSASFLYS | QQSEPEPYT | AASGFTLSGDWIH | EISAAGGYTD | ARESRVS-------FEAAMDY |
| 4rgm | RASQEISDYLT | YVASSLDS | LQYANYPWT | KASGYIFTIAGIQ | WINTHSGVPE | ARIYYGN-------NGGVMDY |
| 4k2u | RAGQDISNYLN | YYTSRLHS | QQGSTFPWT | TASGFNIKDNYMH | RIDPANGNTK | ARHYDGY-------FLYYFEY |
| 3ulv | RASQSIGLYLA | YAASSLQS | QQGNTLSYT | KGSGYSFTNYWVG | FIDPSDSYTN | ARELYQG-------YMDTFDS |
| 4uao | QASQDIGNNLI | YYATKLAN | LQYKQFPLT | AASGFTFSNYYMA | TITTSGSRSY | ARRGYGG-------YSEDFDY |
| 3wd5 | RASQGIRNYLA | YAASTLQS | QRYNRAPYT | AASGFTFDDYAMH | AITWNSGHID | AKVSYLS-------TASSLDY |
| 3eoa | RASKTISKYLA | YSGSTLQS | QQHNEYPLT | AASGYSFTGHWMN | MIHPSDSETR | ARGIYFY-------GTTYFDY |
| 4bz2 | KASQGINSDLS | YRANRLVD | LQYDEFPLT | KASGYTFTDYNMH | YTYPYNGGIG | VRRGYRY-------DGAHFDY |
| 4cmh | KASQDVSTVVA | YSASYRYI | QQHYSPPYT | KASGYTFTDYWMQ | TIYPGDGDTG | ARGDYYG--------SNSLDY |
| 1ncd | KASQDVSTAVV | YWASTRHI | QQHYSPPWT | KASGYTFTNYGMN | WINTNTGEPT | ARGEDNF--------GSLSDY |
| 1yjd | HASQNIYVWLN | YKASNLHT | QQGQTYPYT | EASGYTFTSYYIH | CIYPGNVNTN | TRSHYGL--------DWNFDV |
| 3ubx | KASEDIYNRIA | SGATSLET | QHYWSSPLT | KASGYSFTNYGMH | WINTYTGEPT | ASAAGIR--------WAWFAW |
| 1rjl | HASQNINVWLN | YMASNLHT | QQGQSFPLT | KASGFTFTSSWMH | EIHPNSGNTH | ARMRYGD--------YYAMDN |
| 4zfo | KASQSVDSNVA | FSASLRFS | QQYNNYPLT | AASGIDFSRYWMS | EINPDSSTIN | ASLYYDY--------GDAMDY |
| 1n8z | RASQDVNTAVA | YSASFLYS | QQHYTTPPT | AASGFNIKDTYIH | RIYPTNGYTR | SRWGGDG--------FYAMDY |
| 4qci | SGDSLGSYFVH | YDDSNRPS | SAFTHNSDV | AASGFTFSSYAMS | YISDDGSLKY | ARHPYWY--------GGQLDL |
| 3pgf | RASQSVSSAVA | YSASSLYS | QQSSYIPVT | AASGFNFSSSYIH | YISSYSGYTS | ARTPWWY--------WSGLDY |
| 2fjg | RASQDVSTAVA | YSASFLYS | QQSYTTPPT | AASGFTISDYWIH | GITPAGGYTY | ARFVFFL--------PYAMDY |
| 2vwe | RASQDISNFLN | YYTSTLHS | QQGKTLPPT | KASGYTFTGFWIH | HINPGNGGTN | ARSYSNY--------VRAMDY |
| 4jqi | RASQSVSSAVA | YSASSLYS | QQYKYVPVT | AASGFNVYSSSIH | SISSYYGYTY | ARSRQFW--------YSGLDY |
| 3mxw | KASQSVSNDLT | YYASNRYT | QQDYGSPPT | KGSGYTFIDEALH | VIRPYSGETN | ARDWER---------GDFFDY |
| 1kb5 | RASKNIYSYLA | YNAKTLGE | QHHYGTPYT | KASGYSFTGYNMN | NIDPYYGGIS | ARSRTD---------LYYFDY |
| 3r1g | RASQDVSTAVA | YSASFLYS | QQFPTYLPT | AASGFTFLGYGIH | WISPAGGSTD | ARGPFS---------PWVMDY |
| 3wih | RASQDISNFLN | YYTSRLHS | QQGNTLPLT | KASGYTFTDYYMN | DIVPNNGDTT | ARFSNY---------VYPFDY |
| 3nid | HASQGISSNIG | YYGTNLVD | VQYAQLPYT | TASGFNIKDTYVH | RIDPANGYTK | VRPLYD---------YYAMDY |
| 3ulu | RASGNIHNYLA | YNAKTLAD | QHFWSTPFT | KASGYIFTTYWIH | EINPNNGRIN | TRVGVM---------ITTFPY |
| 4dgi | RASQNIGTSIH | KYASESIS | QQSNTWPYT | KASGYTFTDYWMH | SIDPSDSYTS | SRSGYG---------YYAMEY |
| 1lk3 | KASESVTSRMH | YKASNLAS | QQSWNGPLT | KASGYTFTDFYIH | YINPNSGYTN | TRGVPG---------NNWFPY |
| 2ypv | KASQDIHNYLN | YRANRLVD | LQYDEFPPT | KASGYSFSDYNMS | IIDPKYGTIN | VRDYYG---------SSYFDY |
| 3s88 | KASQDVTTAVA | YWASTRHT | QQHYSTPLT | AASGFAFNYYDMF | YINSGGGNTY | ARQLYG---------NSFFDY |
| 2zjs | RASQDISNYLN | YYTSRLHS | QQGNTLPWT | KATGYTFSSYWIA | EILPGSGSTN | ARSPYY---------YGNWDY |
| 3l95 | RASQDVSTAVA | YSASFLYS | QQFYTTPST | AASGFTFSSYWIH | RINPPNRSNQ | ARGSGF---------RWVMDY |
| 3liz | KASQDINNYLS | YRADRLVD | LQYDELPYT | AASGFTFSSFAMS | TINSNGASTY | TRDPAG---------RAWFAY |
| 1s78 | KASQDVSIGVA | YSASYRYT | QQYYIYPYT | AASGFTFTDYTMD | DVNPNSGGSI | ARNLGP---------SFYFDY |
| 4g6j | RASQSIGSSLH | KYASQSFS | HQSSSLPFT | AASGFTFSVYGMN | IIWYDGDNQY | ARDLRT----------GPFDY |
| 3sqo | RASQGISSALA | YSASYRYT | QQRYSLWRT | KASGYTFTSYYMH | EISPFGGRTN | ARERPL----------YASDL |
| 3or6 | RASQSIGTDIH | KYASESIS | QQSNRWPFT | KASGYTFTSDWIH | EIIPSYGRAN | ARERGD----------GYFAV |
| 1ynt | RASQDISNYLN | YYTSRLHS | QQGNTLPYT | KGSGYTFTDYGMH | IISTYSGDAS | ARSSTW----------YYFDY |
| 1fsk | KASENVDTYVF | YGPSNRYT | GQSYSYPYT | KASGYTFTSYWIN | NIFPSDSYTN | TRGARD----------TWFAY |
| 4mxv | RASQAVSSAVA | YSASHRYT | QESYSTPWT | AASGYTFTSYVIH | YNNPYNAGTN | SRPTML----------PWFAY |
| 4o02 | RASQDISNYLS | FYTSKLHS | QQGNTFPYT | KASGYTFSSFWMH | YINPRSGYTE | ASFLGR----------GAMDY |
| 4dkf | RASQSISSYLA | YGASSRAS | QQYWSEPVT | AASGFSFTSYGIS | HIDWYGGDTD | ARGGPD----------YAMDV |
| 4oii | RASGNIHNYLA | YNAKTLAD | QHFWSTPRT | KASGYTFTSYWMH | DINPNNGGPS | TIDDG-----------YRFGY |
| 4p59 | RASQGISNWLA | YGASSLQS | QQYSSFPTT | AASGFTFSSYAMS | AINSQGKSTY | ARWGD-----------EGFDI |
| 4l5f | ITSTDIDDDMN | SEGNTLRP | LQSDNLPLT | KASGYSFIGYYIH | EINPRTGDTT | TKRIN-----------WALDY |
| 2bdn | KATEDIYNRLA | SGATSLET | QQFWSAPYT | PASGLNIKDTYMH | RIDPANGNTK | ARGVF-----------GFFDY |
| 1wej | RASGNIHNYLA | YNAKTLAD | QHFWSTPWT | TASGFNIKDTYMH | RIDPASGNTK | AGYDY-----------GNFDY |
| 1jps | RASRDIKSYLN | YYATSLAE | LQHGESPWT | AASGFNIKEYYMH | LIDPEQGNTI | ARDTA-----------AYFDY |
| 2yc1 | RASQSVRSYLA | SDASNRAT | QQYRYSPRT | TGSGFTFDNYAMH | GISRSSGDID | ARGGV-----------GSFDT |
| 2r0l | RASQDVSTAVA | YSASFLYS | QQSYTTPPT | AASGFTISNSGIH | WIYPTGGATD | ARFWW-----------RSFDY |
| 2r0k | RASQDVSTAVA | YSASFLYS | QQSYTTPPT | AASGFTITGSAIH | IINPNGGYTY | ARSAR-----------FSFDY |
| 3n85 | RASQSVSSAVA | YSASSLYS | QQWWWWPST | AASGFSIWWSWIH | SISPSSGWTS | ARWWS-----------SAMDY |
| 3s37 | RASQGIDNWLG | YDASNLDT | QQAKAFPPT | AASGFTFSSYSMN | SISSSSSYIY | ARVTD------------AFDI |
| 2nr6 | KASQIVSTAVA | YSASYRYT | QQHYNSPQT | TASGFNIKDTYIH | RIDPANGNTR | ARWVR------------QMDY |
| 3r08 | QASQDISNYLN | YYTNKLAD | QQYYNYPWT | EASGFTFSGYGMH | YITSSSINIK | ARFDW------------DKNY |
| 1p2c | RASQSISNNLH | KYTSQSMS | QQSGSWPRT | KATGYTFTTYWIE | EILPGSDSTY | ARGDG------------FYVY |
| 4rdq | RASENIYSYLT | YNAKTLTE | QHHYGTPPT | KASGYTFTNYWMH | MIDPSKSETT | AREVY------------YFDY |
| 2uzi | RASQSISSYLN | YSASVLQS | QQSVMIPMT | AASGFTFSTFSMN | YISRTSKTIY | ARGR-------------FFDY |
| 4d9r | ITSTDIDDDMN | SGGNTLRP | LQSDSLPYT | KASGYTFTNYGMN | WINTYTGETT | EREG-------------GVNN |
| 3d85 | RASQSISDYLH | KYASQSIS | QNGHSFPFT | KASGYTFTSNVMH | YINPYNDGTK | ARNW-------------DVAY |
| 2oz4 | RASQSIGTSIH | EYASESIS | QQSNVWPFT | KTSGYTFSEFTMH | GINTINGGSS | ATKG--------------FAY |
| 1fj1 | KASQDINKYIA | HYTSTLQP | LQYDNLQRT | KASGYTFTDYSMY | WINTETGEPT | ARG---------------LDS |
| 2vxt | RASQDIGSKLY | YATSSLDS | LQYASSPYT | KASGYSFTDYFIY | DIDPYNGDTS | ARG---------------LRF |
| 2r4r | KASQDINSYLS | YRANRLVD | LQYDEFPYT | KASGYIFTDYYIN | EIYPGSGNID | VRG---------------FGY |
| 2dtg | RASQDIGGNLY | YATSSLDP | LQYSSSPWT | KASGYTFTNYDIH | WIYPGDGSTK | ARE---------------WAY |
| 2q8b | KASQSVSNDVV | YYASIRYT | QQGFSSPRT | IVSGFKIKDTSMH | RIDPANDNSE | TLS----------------HF |

**Supplementary Table S2, related to Methods.** Amino acid conformation classifications. Geometric parameter distributions and clustering criteria are shown for each amino acid type and conformational clusters with side-chain chi1, chi2, chi3 and chi4 and main-chain phi and psi dihedral angles for the centroid conformations.

| ID | Conf. cluster | PHI | PSI | CHI1 | CHI2 | CHI3 | CHI4 | Conformer Percentage(%) | RMSD  Mean(Å) | RMSD  Variance(Å) |
| --- | --- | --- | --- | --- | --- | --- | --- | --- | --- | --- |
| 1 | A1 | -82.142 | -9.6013 |  |  |  |  | 10.01 | 0.179 | 0.018 |
| 2 | A2 | -116.43 | -154.16 |  |  |  |  | 0.78 | 0.267 | 0.015 |
| 3 | A3 | -67.793 | 142.8 |  |  |  |  | 13.36 | 0.145 | 0.008 |
| 4 | A4 | -61.851 | -41.202 |  |  |  |  | 53.29 | 0.108 | 0.014 |
| 5 | A5 | -107.55 | 127.66 |  |  |  |  | 10.28 | 0.203 | 0.017 |
| 6 | A6 | -148.18 | 151.86 |  |  |  |  | 12.28 | 0.132 | 0.004 |
| Total number of conformer : 154325, Partition Index: 0.0014, Separation Index: 1.65E-08 | | | | | | | | | | |
| 7 | C1 | -78.619 | -25.132 | 76.137 |  |  |  | 9.68 | 0.482 | 0.088 |
| 8 | C2 | -109.11 | 117.19 | -168.43 |  |  |  | 11.66 | 0.259 | 0.016 |
| 9 | C3 | -102.49 | 138.96 | -62.872 |  |  |  | 24.44 | 0.236 | 0.015 |
| 10 | C4 | -68.589 | -34.663 | -72.594 |  |  |  | 36.22 | 0.304 | 0.072 |
| 11 | C5 | -141.95 | 159.21 | 62.667 |  |  |  | 9.62 | 0.233 | 0.016 |
| 12 | C6 | -103.93 | 120.53 | 170.01 |  |  |  | 8.38 | 0.234 | 0.010 |
| Total number of conformer : 29291, Partition Index: 0.0019, Separation Index: 8.94E-08 | | | | | | | | | | |
| 13 | D1 | -90.263 | -5.963 | 58.743 | -12.115 |  |  | 12.86 | 0.672 | 0.147 |
| 14 | D2 | -71.108 | -20.619 | -79.542 | 118.4 |  |  | 10.36 | 0.848 | 0.146 |
| 15 | D3 | -98.758 | 106.39 | -165.14 | -12.913 |  |  | 21.58 | 0.471 | 0.072 |
| 16 | D4 | -67.646 | -34.448 | -71.413 | -25.106 |  |  | 33.07 | 0.384 | 0.098 |
| 17 | D5 | -85.179 | 132.89 | -73.55 | -23.595 |  |  | 11.46 | 0.421 | 0.068 |
| 18 | D6 | -98.215 | 106.8 | 156.72 | 11.861 |  |  | 10.67 | 0.652 | 0.100 |
| Total number of conformer : 85476, Partition Index: 0.0032, Separation Index: 4.66E-08 | | | | | | | | | | |
| 19 | E1 | -69.204 | -18.681 | -109.19 | 81.349 | 27.178 |  | 10.47 | 0.900 | 0.077 |
| 20 | E2 | -103.85 | 125.63 | -74.788 | 149.08 | 3.8893 |  | 13.45 | 0.805 | 0.098 |
| 21 | E3 | -71.368 | -21.747 | -64.094 | -70.332 | -31.718 |  | 12.67 | 0.635 | 0.208 |
| 22 | E4 | -103.96 | 127.14 | -55.92 | -140.21 | -8.1659 |  | 17.93 | 0.807 | 0.083 |
| 23 | E5 | -70.698 | -21.246 | -67.657 | -156.37 | -2.6547 |  | 18.27 | 0.732 | 0.103 |
| 24 | E6 | -72.395 | -8.5072 | 153.26 | 103.48 | 15.235 |  | 10.23 | 0.885 | 0.096 |
| 25 | E7 | -68.109 | -29.53 | -75.578 | 158.95 | -8.4272 |  | 16.98 | 0.547 | 0.105 |
| Total number of conformer : 87635, Partition Index: 0.0031, Separation Index: 4.82E-08 | | | | | | | | | | |
| 26 | F1 | -65.06 | -39.612 | -147.63 | 76.556 |  |  | 19.55 | 0.808 | 0.168 |
| 27 | F2 | -109.68 | 131.88 | -63.333 | 86.014 |  |  | 16.76 | 0.331 | 0.037 |
| 28 | F3 | -62.803 | -43.236 | 167.33 | 69.859 |  |  | 12.48 | 0.460 | 0.230 |
| 29 | F4 | -136.26 | 147.98 | 87.349 | 76.702 |  |  | 11.36 | 0.753 | 0.148 |
| 30 | F5 | -77.92 | -22.377 | -69.948 | -59.487 |  |  | 16.26 | 0.552 | 0.188 |
| 31 | F6 | -108.88 | 134.04 | -60.765 | -77.5 |  |  | 17.50 | 0.596 | 0.288 |
| 32 | F7 | -100.3 | 111.99 | -153.61 | 69.477 |  |  | 6.09 | 0.386 | 0.021 |
| Total number of conformer : 84976, Partition Index: 0.0021, Separation Index: 3.37E-08 | | | | | | | | | | |
| 33 | G1 | 107.04 | -156.68 |  |  |  |  | 12.79 | 0.239 | 0.008 |
| 34 | G2 | -119.52 | -160.73 |  |  |  |  | 9.23 | 0.209 | 0.007 |
| 35 | G3 | -110.38 | 153.24 |  |  |  |  | 19.19 | 0.234 | 0.010 |
| 36 | G4 | -66.23 | -34.586 |  |  |  |  | 26.27 | 0.152 | 0.012 |
| 37 | G5 | 86.04 | 6.9536 |  |  |  |  | 23.96 | 0.161 | 0.009 |
| 38 | G6 | 116.61 | 158.61 |  |  |  |  | 8.57 | 0.206 | 0.008 |
| Total number of conformer : 125926, Partition Index: 9.69E-04, Separation Index: 1.26E-08 | | | | | | | | | | |
| 39 | H1 | -87.461 | 127.8 | -158.4 | -81.972 |  |  | 7.81 | 0.397 | 0.047 |
| 40 | H2 | -102.06 | 103.43 | -69.454 | 84.629 |  |  | 5.99 | 0.821 | 0.144 |
| 41 | H3 | -107.11 | 112.5 | 155.25 | 60.447 |  |  | 7.34 | 0.829 | 0.115 |
| 42 | H4 | -75.625 | -24.897 | -65.608 | 110.29 |  |  | 13.75 | 0.534 | 0.095 |
| 43 | H5 | -64.036 | -38.092 | -162.63 | 68.871 |  |  | 5.46 | 0.351 | 0.055 |
| 44 | H6 | -67.634 | -32.834 | -73.74 | -71.611 |  |  | 15.24 | 0.665 | 0.183 |
| 45 | H7 | -114.27 | 136.78 | -65.127 | 85.436 |  |  | 8.67 | 0.563 | 0.143 |
| 46 | H8 | -67.795 | -28.218 | 142.26 | -58.257 |  |  | 11.67 | 0.977 | 0.085 |
| 47 | H9 | -99.1 | 6.1504 | -60.747 | -74.739 |  |  | 7.20 | 0.385 | 0.042 |
| 48 | H10 | -113.58 | 136.07 | -56.917 | -82.811 |  |  | 16.86 | 0.549 | 0.158 |
| Total number of conformer : 42465, Partition Index: 0.0028, Separation Index: 9.08E-08 | | | | | | | | | | |
| 49 | I1 | -65.415 | -43.135 | -67.744 | 166.89 |  |  | 32.68 | 0.367 | 0.195 |
| 50 | I2 | -119.12 | 122.99 | -62.772 | 166.46 |  |  | 19.41 | 0.283 | 0.105 |
| 51 | I3 | -67.241 | -41.923 | -62.275 | -71.517 |  |  | 11.01 | 0.384 | 0.106 |
| 52 | I4 | -101.22 | 121.42 | -55.136 | -61.119 |  |  | 9.21 | 0.231 | 0.044 |
| 53 | I5 | -109.86 | 116.21 | -54.356 | -171.17 |  |  | 6.69 | 0.422 | 0.174 |
| 54 | I6 | -120.56 | 145.74 | 64.441 | 164.77 |  |  | 8.78 | 0.482 | 0.156 |
| 55 | I7 | -91.956 | 118.75 | -65.116 | 163.04 |  |  | 12.21 | 0.292 | 0.082 |
| Total number of conformer : 123327, Partition Index: 0.0014, Separation Index: 1.40E-08 | | | | | | | | | | |
| 56 | K1 | -73.566 | -7.1543 | -82.14 | 157.34 | -156.49 | 151.07 | 9.96 | 0.976 | 0.143 |
| 57 | K2 | -79.647 | 14.92 | -82.202 | 145.3 | -137.44 | -107 | 9.94 | 1.054 | 0.097 |
| 58 | K3 | -72.18 | -14.126 | -67.263 | -127.24 | -119.63 | -129.34 | 9.58 | 0.758 | 0.053 |
| 59 | K4 | -83.063 | 34.099 | 87 | 101.4 | 92.003 | 52.033 | 6.35 | 1.073 | 0.025 |
| 60 | K5 | -73.157 | -12.036 | -66.707 | -122.48 | -134.67 | 135.17 | 7.16 | 0.738 | 0.053 |
| 61 | K6 | -71.114 | -15.814 | -80.191 | 151.16 | 133.88 | 138.9 | 7.12 | 0.707 | 0.083 |
| 62 | K7 | -96.055 | 95.285 | -62.724 | 122.82 | 98.091 | 78.115 | 5.17 | 0.881 | 0.031 |
| 63 | K8 | -101.31 | 110.26 | -49.99 | -109.13 | 136.04 | -109.05 | 5.37 | 1.010 | 0.120 |
| 64 | K9 | -97.122 | 103.08 | -54.428 | -90.6 | -123.7 | 106.2 | 5.50 | 0.849 | 0.034 |
| 65 | K10 | -73.265 | -16.224 | -67.496 | -142.71 | 152.6 | -147.65 | 6.65 | 0.779 | 0.103 |
| 66 | K11 | -76.411 | -0.66278 | -66.449 | -138.29 | 141.03 | 128.09 | 11.28 | 0.930 | 0.096 |
| 67 | K12 | -99.686 | 111.97 | -55.974 | -102.33 | -130.79 | -114 | 7.59 | 0.791 | 0.056 |
| 68 | K13 | -74.477 | -4.71 | -80.937 | 144.52 | 130.59 | -132.08 | 8.58 | 0.872 | 0.083 |
| Total number of conformer : 67791, Partition Index: 0.0024, Separation Index: 4.28E-08 | | | | | | | | | | |
| 69 | L1 | -70.291 | -31.002 | -69.761 | 168.94 |  |  | 27.52 | 0.224 | 0.029 |
| 70 | L2 | -101.8 | 137.93 | -68.637 | 160.41 |  |  | 23.36 | 0.592 | 0.178 |
| 71 | L3 | -70.704 | -30.101 | -73.394 | -168.06 |  |  | 12.14 | 0.336 | 0.068 |
| 72 | L4 | -63.104 | -43.409 | 171.38 | 62.568 |  |  | 7.95 | 0.202 | 0.051 |
| 73 | L5 | -96.064 | 134.03 | -68.125 | -164.58 |  |  | 8.29 | 0.376 | 0.076 |
| 74 | L6 | -109.73 | 124.02 | 167.53 | 66.821 |  |  | 7.66 | 0.318 | 0.076 |
| 75 | L7 | -66.259 | -30.949 | -158.26 | 55.694 |  |  | 13.09 | 0.427 | 0.074 |
| Total number of conformer : 195756, Partition Index: 9.70E-04, Separation Index: 5.84E-09 | | | | | | | | | | |
| 76 | M1 | -71.922 | -22.202 | -66 | -160.29 | 73.812 |  | 7.29 | 0.618 | 0.126 |
| 77 | M2 | -106.17 | 110.29 | -76.654 | 157.82 | -88.483 |  | 9.65 | 0.869 | 0.127 |
| 78 | M3 | -108.7 | 132.87 | -52.977 | -153.94 | 70.182 |  | 8.50 | 0.753 | 0.105 |
| 79 | M4 | -68.158 | -32.486 | -75.158 | 164.1 | 71.084 |  | 14.45 | 0.614 | 0.144 |
| 80 | M5 | -70.699 | -24.971 | -66.77 | -56.255 | 100.53 |  | 5.67 | 0.438 | 0.116 |
| 81 | M6 | -69.391 | -28.276 | -68.679 | -56.41 | -70.667 |  | 15.81 | 0.447 | 0.125 |
| 82 | M7 | -103.92 | 124.05 | -77.263 | 156.88 | 70.712 |  | 7.38 | 0.678 | 0.086 |
| 83 | M8 | -98.782 | 131.27 | -58.696 | -78.65 | -72.985 |  | 13.48 | 0.730 | 0.110 |
| 84 | M9 | -69.839 | -27.824 | -68.4 | -162.13 | -79.179 |  | 9.37 | 0.461 | 0.080 |
| 85 | M10 | -103.69 | 94.787 | 144.15 | 125.14 | 46.469 |  | 8.39 | 0.894 | 0.058 |
| Total number of conformer : 42216, Partition Index: 0.0024, Separation Index: 7.66E-08 | | | | | | | | | | |
| 86 | N1 | -83.917 | -5.6463 | -73.371 | -57.038 |  |  | 13.54 | 0.525 | 0.144 |
| 87 | N2 | -95.342 | 6.3002 | 63.754 | -12.46 |  |  | 11.08 | 0.677 | 0.144 |
| 88 | N3 | -97.451 | 132.17 | -65.91 | -50.797 |  |  | 12.36 | 0.397 | 0.054 |
| 89 | N4 | -101.73 | 109.12 | 154.28 | -4.7755 |  |  | 9.69 | 0.713 | 0.062 |
| 90 | N5 | -96.178 | 104.32 | -164.09 | -25.792 |  |  | 16.03 | 0.468 | 0.063 |
| 91 | N6 | -97.238 | 122.17 | -70.893 | 97.232 |  |  | 6.60 | 0.650 | 0.096 |
| 92 | N7 | -63.285 | -34.657 | -73.964 | -22.311 |  |  | 20.50 | 0.412 | 0.114 |
| 93 | N8 | -69.688 | -24.427 | -73.737 | 128.67 |  |  | 10.20 | 0.629 | 0.149 |
| Total number of conformer : 68552, Partition Index: 0.0035, Separation Index: 6.59E-08 | | | | | | | | | | |
| 94 | P1 | -61.149 | 134.91 |  |  |  |  | 30.23 | 0.304 | 0.016 |
| 95 | P2 | -71.04 | 155.62 |  |  |  |  | 31.73 | 0.248 | 0.017 |
| 96 | P3 | -71.574 | -12.952 |  |  |  |  | 13.73 | 0.293 | 0.021 |
| 97 | P4 | -56.097 | -38.952 |  |  |  |  | 24.31 | 0.318 | 0.029 |
| Total number of conformer : 68722, Partition Index: 0.0013, Separation Index: 2.10E-08 | | | | | | | | | | |
| 98 | Q1 | -71.575 | -20.079 | -63.904 | -146.57 | 64.741 |  | 11.77 | 0.828 | 0.115 |
| 99 | Q2 | -71.922 | -21.656 | -71.791 | 159.13 | 46.315 |  | 8.76 | 0.601 | 0.081 |
| 100 | Q3 | -106.98 | 127.94 | -52.716 | -149.09 | 31.989 |  | 11.86 | 0.864 | 0.087 |
| 101 | Q4 | -69.086 | -28.723 | -70.292 | 163.88 | -45.085 |  | 12.29 | 0.601 | 0.127 |
| 102 | Q5 | -65.935 | -30.796 | -157.17 | 65.383 | 45.805 |  | 6.42 | 0.566 | 0.092 |
| 103 | Q6 | -72.618 | -24.029 | -64.127 | -60.339 | -46.113 |  | 9.39 | 0.554 | 0.144 |
| 104 | Q7 | -106.08 | 131.59 | -72.175 | 156.16 | -8.9235 |  | 13.91 | 0.805 | 0.086 |
| 105 | Q8 | -101.83 | 132.77 | -55.142 | -73.412 | -41.454 |  | 7.85 | 0.750 | 0.197 |
| 106 | Q9 | -71.113 | -22.69 | -64.129 | -159.53 | -41.663 |  | 8.85 | 0.624 | 0.102 |
| 107 | Q10 | -73.95 | -0.81599 | 155.61 | 99.656 | 22.42 |  | 8.90 | 0.947 | 0.071 |
| Total number of conformer : 54694, Partition Index: 0.0028, Separation Index: 7.49E-08 | | | | | | | | | | |
| 108 | R1 | -69.778 | -20.085 | -68.775 | -145.72 | -72.154 | -85.998 | 10.28 | 0.932 | 0.165 |
| 109 | R2 | -77.786 | 12.227 | -74.363 | 140.12 | -93.52 | 114.76 | 9.50 | 1.412 | 0.166 |
| 110 | R3 | -82.297 | 21.243 | -58.365 | -123.51 | 88.317 | -103.28 | 10.59 | 1.442 | 0.121 |
| 111 | R4 | -80.285 | 16.264 | -64.978 | 137.48 | 92.566 | 92.867 | 10.21 | 1.088 | 0.069 |
| 112 | R5 | -102.78 | 102.96 | -49.575 | -92.159 | -89.086 | -76.521 | 8.23 | 1.074 | 0.047 |
| 113 | R6 | -85.754 | 42.382 | -52.476 | -117.67 | 79.797 | 81.037 | 10.60 | 1.446 | 0.132 |
| 114 | R7 | -77.826 | 7.2829 | -73.277 | 140.23 | 68.506 | -116.46 | 10.79 | 1.216 | 0.125 |
| 115 | R8 | -89.179 | 57.151 | 34.91 | 94.565 | 40.8 | 39.151 | 7.57 | 1.519 | 0.033 |
| 116 | R9 | -77.218 | 1.4227 | -67.373 | -130.66 | -84.294 | 112.95 | 13.09 | 1.167 | 0.093 |
| 117 | R10 | -89.116 | 58.75 | -46.323 | 115.46 | -61.04 | -66.681 | 9.14 | 1.939 | 0.099 |
| Total number of conformer : 83239, Partition Index: 0.0039, Separation Index: 5.65E-08 | | | | | | | | | | |
| 118 | S1 | -66.297 | -38.237 | -71.084 |  |  |  | 21.85 | 0.324 | 0.087 |
| 119 | S2 | -145.89 | 157.4 | 66.36 |  |  |  | 11.41 | 0.174 | 0.008 |
| 120 | S3 | -72.687 | -26.372 | 69.151 |  |  |  | 25.74 | 0.318 | 0.071 |
| 121 | S4 | -103.47 | 129.9 | 168.87 |  |  |  | 11.23 | 0.240 | 0.009 |
| 122 | S5 | -98.997 | 138.07 | -64.032 |  |  |  | 11.63 | 0.242 | 0.014 |
| 123 | S6 | -77.462 | 156.11 | 67.174 |  |  |  | 11.32 | 0.176 | 0.009 |
| 124 | S7 | -116.05 | 125.46 | -168.07 |  |  |  | 6.81 | 0.286 | 0.017 |
| Total number of conformer : 98527, Partition Index: 0.0019, Separation Index: 2.63E-08 | | | | | | | | | | |
| 125 | T1 | -111.33 | 157.03 | 65.304 |  |  |  | 25.47 | 0.279 | 0.053 |
| 126 | T2 | -110.24 | 130.88 | -70.67 |  |  |  | 27.39 | 0.354 | 0.073 |
| 127 | T3 | -89.554 | -17.303 | 60.142 |  |  |  | 22.19 | 0.312 | 0.067 |
| 128 | T4 | -65.932 | -44.229 | -62.341 |  |  |  | 24.94 | 0.222 | 0.084 |
| Total number of conformer : 99530, Partition Index: 0.0021, Separation Index: 2.40E-08 | | | | | | | | | | |
| 129 | V1 | -87.803 | -12.843 | -52.794 |  |  |  | 7.26 | 0.312 | 0.049 |
| 130 | V2 | -65.416 | -43.004 | 168.77 |  |  |  | 28.50 | 0.190 | 0.047 |
| 131 | V3 | -111.44 | 124.14 | -173.66 |  |  |  | 17.30 | 0.170 | 0.008 |
| 132 | V4 | -69.349 | -41.751 | -170.83 |  |  |  | 4.58 | 0.194 | 0.027 |
| 133 | V5 | -123.51 | 153.34 | -53.799 |  |  |  | 13.09 | 0.320 | 0.085 |
| 134 | V6 | -108.45 | 126.71 | 170.37 |  |  |  | 29.28 | 0.263 | 0.057 |
| Total number of conformer : 151820, Partition Index: 0.001, Separation Index: 9.87E-09 | | | | | | | | | | |
| 135 | W1 | -72.025 | -14.996 | 63.363 | -87.685 |  |  | 6.06 | 0.325 | 0.065 |
| 136 | W2 | -116.25 | 136.68 | -68.587 | -76.754 |  |  | 14.26 | 1.131 | 0.299 |
| 137 | W3 | -64.952 | -37.487 | 168.14 | 75.165 |  |  | 9.80 | 0.614 | 0.212 |
| 138 | W4 | -71.368 | -27.338 | -69.821 | 103.97 |  |  | 15.22 | 0.370 | 0.055 |
| 139 | W5 | -62.471 | -41.706 | -168.81 | 80.36 |  |  | 6.58 | 0.314 | 0.089 |
| 140 | W6 | -72.518 | -23.709 | -66.421 | -16.91 |  |  | 8.90 | 0.429 | 0.079 |
| 141 | W7 | -90.491 | 136.08 | -65.997 | 89.713 |  |  | 13.36 | 0.508 | 0.233 |
| 142 | W8 | -64.175 | -37.517 | 168.61 | -102.35 |  |  | 6.74 | 0.434 | 0.082 |
| 143 | W9 | -64.164 | -35.801 | -165.14 | -101.32 |  |  | 5.46 | 0.427 | 0.074 |
| 144 | W10 | -118.18 | 140.7 | -56.733 | 72.332 |  |  | 13.61 | 0.836 | 0.448 |
| Total number of conformer : 29116, Partition Index: 0.0019, Separation Index: 1.06E-07 | | | | | | | | | | |
| 145 | Y1 | -80.429 | -20.716 | -67.526 | -64.872 |  |  | 16.25 | 0.568 | 0.206 |
| 146 | Y2 | -62.298 | -44.855 | 167.55 | 70.774 |  |  | 12.78 | 0.557 | 0.157 |
| 147 | Y3 | -111.32 | 137.51 | -60.034 | -78.785 |  |  | 16.70 | 0.625 | 0.319 |
| 148 | Y4 | -113.02 | 130.92 | 151.94 | 65.842 |  |  | 11.81 | 0.755 | 0.086 |
| 149 | Y5 | -114.67 | 140.18 | -59.911 | 84.664 |  |  | 16.00 | 0.329 | 0.052 |
| 150 | Y6 | -103.63 | 122.25 | -160.24 | 68.438 |  |  | 6.27 | 0.394 | 0.016 |
| 151 | Y7 | -82.448 | -18.398 | -65.626 | 95.415 |  |  | 11.84 | 0.457 | 0.075 |
| 152 | Y8 | -62.561 | -42.841 | -167.11 | 72.545 |  |  | 8.34 | 0.341 | 0.015 |
| Total number of conformer : 71876, Partition Index: 0.0018, Separation Index: 3.56E-08 | | | | | | | | | | |

**Supplementary Table S3, related to Methods**. Atom types in protein structures.

| ID # | Atom Type | Radius | Description |
| --- | --- | --- | --- |
| 1 | NH1 | 1.65 | Backbone NH |
| 2 | C | 1.76 | Backbone C |
| 3 | CH1E | 1.87 | Backbone CA (exc. Gly) |
| 4 | O | 1.40 | Backbone O |
| 5 | CH0 | 1.76 | Arg CZ, Asn CG, Asp CG, Gln CD, Glu CD |
| 6 | CH1S | 1.87 | Sidechain CH1: Ile CB, Leu CG, Thr CB, Val CB |
| 7 | CH2E | 1.87 | Tetrahedral CH2 (except CH2P,CH2G) All CB |
| 8 | CH3E | 1.87 | Tetrahedral CH3 |
| 9 | CR1E | 1.76 | Aromatic CH (except CR1W, CRHH, CR1H) |
| 10 | OH1 | 1.40 | Alcohol OH (Ser OG, Thr OG1, Tyr OH) |
| 11 | OC | 1.40 | Carboxyl O (Asp OD1, OD2, Glu OE1, OE2) |
| 12 | OS | 1.40 | Sidechain O: Asn OD1, Gln OE1 |
| 13 | CH2G | 1.87 | Gly CA |
| 14 | CH2P | 1.87 | Pro CB, CG, CD |
| 15 | NH1S | 1.65 | Sidechain NH: Arg NE, His ND1, NE1, Trp NE1 |
| 16 | NC2 | 1.65 | Arg NH1, NH2 |
| 17 | NH2 | 1.65 | Asn ND2, Gln NE2 |
| 18 | CR1W | 1.76 | Trp CZ2, CH2 |
| 19 | CY2 | 1.76 | Tyr CZ |
| 20 | SC | 1.85 | Cys S |
| 21 | CF | 1.76 | Phe CG |
| 22 | SM | 1.85 | Met S |
| 23 | CY | 1.76 | Tyr CG |
| 24 | CW | 1.76 | Trp CD2, CE2 |
| 25 | CRHH | 1.76 | His CE1 |
| 26 | NH3 | 1.50 | Lys NZ |
| 27 | CR1H | 1.76 | His CD2 |
| 28 | C5 | 1.76 | His CG |
| 29 | N | 1.65 | Pro N |
| 30 | C5W | 1.76 | Trp CG |
| 31 | HOH | 1.40 | Water |
|  |  |  |  |

**Supplementary Table S4, related to Figure 4**. CDR variant sequences of M9. The sequence ranges of the CDR residue positions are labelled in the first row by the IMGT numbering.

| Name | **L1(24~40)** | **L2(55~69)** | **L3(105~117)** | **H1(24~40)** | **H2(55~66)** | **H3(105~117)** |
| --- | --- | --- | --- | --- | --- | --- |
| M9 | RASQDVNDGVA | SGSPWLYS | QQYFNWPIT | AASGFTIDNYGIH | WIWPYGGSTY | ARGYYWYDY |
| L1-01 | RASQDVRGGVA | SGSPWLYS | QQYFNWPIT | AASGFTIDNYGIH | WIWPYGGSTY | ARGYYWYDY |
| L1-02 | RASQDVGEDVA | SGSPWLYS | QQYFNWPIT | AASGFTIDNYGIH | WIWPYGGSTY | ARGYYWYDY |
| L1-03 | RASQDVGEGVA | SGSPWLYS | QQYFNWPIT | AASGFTIDNYGIH | WIWPYGGSTY | ARGYYWYDY |
| L1-04 | RASQDVGWDVA | SGSPWLYS | QQYFNWPIT | AASGFTIDNYGIH | WIWPYGGSTY | ARGYYWYDY |
| L1-05 | RASQDVQQAVA | SGSPWLYS | QQYFNWPIT | AASGFTIDNYGIH | WIWPYGGSTY | ARGYYWYDY |
| L1-06 | RASQDVGGDVA | SGSPWLYS | QQYFNWPIT | AASGFTIDNYGIH | WIWPYGGSTY | ARGYYWYDY |
| L1-07 | RASQDVGGEVA | SGSPWLYS | QQYFNWPIT | AASGFTIDNYGIH | WIWPYGGSTY | ARGYYWYDY |
| L1-08 | RASQDVGRGVA | SGSPWLYS | QQYFNWPIT | AASGFTIDNYGIH | WIWPYGGSTY | ARGYYWYDY |
| L1-09 | RASQDVAKGVA | SGSPWLYS | QQYFNWPIT | AASGFTIDNYGIH | WIWPYGGSTY | ARGYYWYDY |
| L1-10 | RASQDVSGGVA | SGSPWLYS | QQYFNWPIT | AASGFTIDNYGIH | WIWPYGGSTY | ARGYYWYDY |
| L1-11 | RASQDVRRGVA | SGSPWLYS | QQYFNWPIT | AASGFTIDNYGIH | WIWPYGGSTY | ARGYYWYDY |
| L1-12 | RASQDVGTTVA | SGSPWLYS | QQYFNWPIT | AASGFTIDNYGIH | WIWPYGGSTY | ARGYYWYDY |
| L1-13 | RASQDVGKGVA | SGSPWLYS | QQYFNWPIT | AASGFTIDNYGIH | WIWPYGGSTY | ARGYYWYDY |
| L1-14 | RASQDVGRDVA | SGSPWLYS | QQYFNWPIT | AASGFTIDNYGIH | WIWPYGGSTY | ARGYYWYDY |
| L1-15 | RASQDVGGGVA | SGSPWLYS | QQYFNWPIT | AASGFTIDNYGIH | WIWPYGGSTY | ARGYYWYDY |
| L1-16 | RASQDVGKDVA | SGSPWLYS | QQYFNWPIT | AASGFTIDNYGIH | WIWPYGGSTY | ARGYYWYDY |
| L1-17 | RASQDVRNAVA | SGSPWLYS | QQYFNWPIT | AASGFTIDNYGIH | WIWPYGGSTY | ARGYYWYDY |
| L1-18 | RASQDVTSSVA | SGSPWLYS | QQYFNWPIT | AASGFTIDNYGIH | WIWPYGGSTY | ARGYYWYDY |
| L1-19 | RASQDVKGAVA | SGSPWLYS | QQYFNWPIT | AASGFTIDNYGIH | WIWPYGGSTY | ARGYYWYDY |
| L1-20 | RASQDVGMTVA | SGSPWLYS | QQYFNWPIT | AASGFTIDNYGIH | WIWPYGGSTY | ARGYYWYDY |
| L1-21 | RASQDVTSAVA | SGSPWLYS | QQYFNWPIT | AASGFTIDNYGIH | WIWPYGGSTY | ARGYYWYDY |
| L1-22 | RASQDVNRGVA | SGSPWLYS | QQYFNWPIT | AASGFTIDNYGIH | WIWPYGGSTY | ARGYYWYDY |
| L1-23 | RASQDVGNGVA | SGSPWLYS | QQYFNWPIT | AASGFTIDNYGIH | WIWPYGGSTY | ARGYYWYDY |
| L1-24 | RASQDVREAVA | SGSPWLYS | QQYFNWPIT | AASGFTIDNYGIH | WIWPYGGSTY | ARGYYWYDY |
| L1-25 | RASQDVQDAVA | SGSPWLYS | QQYFNWPIT | AASGFTIDNYGIH | WIWPYGGSTY | ARGYYWYDY |
| L1-26 | RASQDVRGAVA | SGSPWLYS | QQYFNWPIT | AASGFTIDNYGIH | WIWPYGGSTY | ARGYYWYDY |
| L1-27 | RASQDVAEGVA | SGSPWLYS | QQYFNWPIT | AASGFTIDNYGIH | WIWPYGGSTY | ARGYYWYDY |
| L1-28 | RASQDVGGTVA | SGSPWLYS | QQYFNWPIT | AASGFTIDNYGIH | WIWPYGGSTY | ARGYYWYDY |
| L1-29 | RASQDVSRGVA | SGSPWLYS | QQYFNWPIT | AASGFTIDNYGIH | WIWPYGGSTY | ARGYYWYDY |
| L1-30 | RASQDVSTAVA | SGSPWLYS | QQYFNWPIT | AASGFTIDNYGIH | WIWPYGGSTY | ARGYYWYDY |
| L1-31 | RASQDVVDAVA | SGSPWLYS | QQYFNWPIT | AASGFTIDNYGIH | WIWPYGGSTY | ARGYYWYDY |
| L1-32 | RASQDVGMGVA | SGSPWLYS | QQYFNWPIT | AASGFTIDNYGIH | WIWPYGGSTY | ARGYYWYDY |
| L1-33 | RASQDVEGGVA | SGSPWLYS | QQYFNWPIT | AASGFTIDNYGIH | WIWPYGGSTY | ARGYYWYDY |
| L1-34 | RASQDVSDAVA | SGSPWLYS | QQYFNWPIT | AASGFTIDNYGIH | WIWPYGGSTY | ARGYYWYDY |
| L1-35 | RASQDVEGAVA | SGSPWLYS | QQYFNWPIT | AASGFTIDNYGIH | WIWPYGGSTY | ARGYYWYDY |
| L1-36 | RASQDVANGVA | SGSPWLYS | QQYFNWPIT | AASGFTIDNYGIH | WIWPYGGSTY | ARGYYWYDY |
| L2-01 | RASQDVNDGVA | DENVRLYS | QQYFNWPIT | AASGFTIDNYGIH | WIWPYGGSTY | ARGYYWYDY |
| L2-02 | RASQDVNDGVA | DKGqRLYS | QQYFNWPIT | AASGFTIDNYGIH | WIWPYGGSTY | ARGYYWYDY |
| L2-03 | RASQDVNDGVA | DqARLLYS | QQYFNWPIT | AASGFTIDNYGIH | WIWPYGGSTY | ARGYYWYDY |
| L2-04 | RASQDVNDGVA | DRDRILYS | QQYFNWPIT | AASGFTIDNYGIH | WIWPYGGSTY | ARGYYWYDY |
| L2-05 | RASQDVNDGVA | DRGKYLYS | QQYFNWPIT | AASGFTIDNYGIH | WIWPYGGSTY | ARGYYWYDY |
| L2-06 | RASQDVNDGVA | DRGTWLYS | QQYFNWPIT | AASGFTIDNYGIH | WIWPYGGSTY | ARGYYWYDY |
| L2-07 | RASQDVNDGVA | DRMIVLYS | QQYFNWPIT | AASGFTIDNYGIH | WIWPYGGSTY | ARGYYWYDY |
| L2-08 | RASQDVNDGVA | DRMSMLYS | QQYFNWPIT | AASGFTIDNYGIH | WIWPYGGSTY | ARGYYWYDY |
| L2-09 | RASQDVNDGVA | DRVIWLYS | QQYFNWPIT | AASGFTIDNYGIH | WIWPYGGSTY | ARGYYWYDY |
| L2-10 | RASQDVNDGVA | ESVTGLYS | QQYFNWPIT | AASGFTIDNYGIH | WIWPYGGSTY | ARGYYWYDY |
| L2-11 | RASQDVNDGVA | FQMGELYS | QQYFNWPIT | AASGFTIDNYGIH | WIWPYGGSTY | ARGYYWYDY |
| L2-12 | RASQDVNDGVA | GALGGLYS | QQYFNWPIT | AASGFTIDNYGIH | WIWPYGGSTY | ARGYYWYDY |
| L2-13 | RASQDVNDGVA | GENEGLYS | QQYFNWPIT | AASGFTIDNYGIH | WIWPYGGSTY | ARGYYWYDY |
| L2-14 | RASQDVNDGVA | GGENRLYS | QQYFNWPIT | AASGFTIDNYGIH | WIWPYGGSTY | ARGYYWYDY |
| L2-15 | RASQDVNDGVA | GGFGMLYS | QQYFNWPIT | AASGFTIDNYGIH | WIWPYGGSTY | ARGYYWYDY |
| L2-16 | RASQDVNDGVA | GGITSLYS | QQYFNWPIT | AASGFTIDNYGIH | WIWPYGGSTY | ARGYYWYDY |
| L2-17 | RASQDVNDGVA | GGNNWLYS | QQYFNWPIT | AASGFTIDNYGIH | WIWPYGGSTY | ARGYYWYDY |
| L2-18 | RASQDVNDGVA | GGWGQLYS | QQYFNWPIT | AASGFTIDNYGIH | WIWPYGGSTY | ARGYYWYDY |
| L2-19 | RASQDVNDGVA | GIGGILYS | QQYFNWPIT | AASGFTIDNYGIH | WIWPYGGSTY | ARGYYWYDY |
| L2-20 | RASQDVNDGVA | GIHGTLYS | QQYFNWPIT | AASGFTIDNYGIH | WIWPYGGSTY | ARGYYWYDY |
| L2-21 | RASQDVNDGVA | GIKGLLYS | QQYFNWPIT | AASGFTIDNYGIH | WIWPYGGSTY | ARGYYWYDY |
| L2-22 | RASQDVNDGVA | GLRGGLYS | QQYFNWPIT | AASGFTIDNYGIH | WIWPYGGSTY | ARGYYWYDY |
| L2-23 | RASQDVNDGVA | GMqGRLYS | QQYFNWPIT | AASGFTIDNYGIH | WIWPYGGSTY | ARGYYWYDY |
| L2-24 | RASQDVNDGVA | GNMGYLYS | QQYFNWPIT | AASGFTIDNYGIH | WIWPYGGSTY | ARGYYWYDY |
| L2-25 | RASQDVNDGVA | GqYKLLYS | QQYFNWPIT | AASGFTIDNYGIH | WIWPYGGSTY | ARGYYWYDY |
| L2-26 | RASQDVNDGVA | GRNDGLYS | QQYFNWPIT | AASGFTIDNYGIH | WIWPYGGSTY | ARGYYWYDY |
| L2-27 | RASQDVNDGVA | GRWDMLYS | QQYFNWPIT | AASGFTIDNYGIH | WIWPYGGSTY | ARGYYWYDY |
| L2-28 | RASQDVNDGVA | GSWNMLYS | QQYFNWPIT | AASGFTIDNYGIH | WIWPYGGSTY | ARGYYWYDY |
| L2-29 | RASQDVNDGVA | GTAGRLYS | QQYFNWPIT | AASGFTIDNYGIH | WIWPYGGSTY | ARGYYWYDY |
| L2-30 | RASQDVNDGVA | GTqNLLYS | QQYFNWPIT | AASGFTIDNYGIH | WIWPYGGSTY | ARGYYWYDY |
| L2-31 | RASQDVNDGVA | GVANLLYS | QQYFNWPIT | AASGFTIDNYGIH | WIWPYGGSTY | ARGYYWYDY |
| L2-32 | RASQDVNDGVA | GVNNLLYS | QQYFNWPIT | AASGFTIDNYGIH | WIWPYGGSTY | ARGYYWYDY |
| L2-33 | RASQDVNDGVA | GWDRLLYS | QQYFNWPIT | AASGFTIDNYGIH | WIWPYGGSTY | ARGYYWYDY |
| L2-34 | RASQDVNDGVA | GWNGLLYS | QQYFNWPIT | AASGFTIDNYGIH | WIWPYGGSTY | ARGYYWYDY |
| L2-35 | RASQDVNDGVA | GWNKFLYS | QQYFNWPIT | AASGFTIDNYGIH | WIWPYGGSTY | ARGYYWYDY |
| L2-36 | RASQDVNDGVA | GWNNHLYS | QQYFNWPIT | AASGFTIDNYGIH | WIWPYGGSTY | ARGYYWYDY |
| L2-37 | RASQDVNDGVA | GWNNKLYS | QQYFNWPIT | AASGFTIDNYGIH | WIWPYGGSTY | ARGYYWYDY |
| L2-38 | RASQDVNDGVA | GWNNWLYS | QQYFNWPIT | AASGFTIDNYGIH | WIWPYGGSTY | ARGYYWYDY |
| L2-39 | RASQDVNDGVA | GWRDALYS | QQYFNWPIT | AASGFTIDNYGIH | WIWPYGGSTY | ARGYYWYDY |
| L2-40 | RASQDVNDGVA | GYGGLLYS | QQYFNWPIT | AASGFTIDNYGIH | WIWPYGGSTY | ARGYYWYDY |
| L2-41 | RASQDVNDGVA | GYRGLLYS | QQYFNWPIT | AASGFTIDNYGIH | WIWPYGGSTY | ARGYYWYDY |
| L2-42 | RASQDVNDGVA | KERRLLYS | QQYFNWPIT | AASGFTIDNYGIH | WIWPYGGSTY | ARGYYWYDY |
| L2-43 | RASQDVNDGVA | KMREVLYS | QQYFNWPIT | AASGFTIDNYGIH | WIWPYGGSTY | ARGYYWYDY |
| L2-44 | RASQDVNDGVA | NEGGRLYS | QQYFNWPIT | AASGFTIDNYGIH | WIWPYGGSTY | ARGYYWYDY |
| L2-45 | RASQDVNDGVA | NEKDNLYS | QQYFNWPIT | AASGFTIDNYGIH | WIWPYGGSTY | ARGYYWYDY |
| L2-46 | RASQDVNDGVA | NIRLGLYS | QQYFNWPIT | AASGFTIDNYGIH | WIWPYGGSTY | ARGYYWYDY |
| L2-47 | RASQDVNDGVA | NVGEGLYS | QQYFNWPIT | AASGFTIDNYGIH | WIWPYGGSTY | ARGYYWYDY |
| L2-48 | RASQDVNDGVA | RGRGILYS | QQYFNWPIT | AASGFTIDNYGIH | WIWPYGGSTY | ARGYYWYDY |
| L2-49 | RASQDVNDGVA | RMEDFLYS | QQYFNWPIT | AASGFTIDNYGIH | WIWPYGGSTY | ARGYYWYDY |
| L2-50 | RASQDVNDGVA | RQMIELYS | QQYFNWPIT | AASGFTIDNYGIH | WIWPYGGSTY | ARGYYWYDY |
| L2-51 | RASQDVNDGVA | RRDRHLYS | QQYFNWPIT | AASGFTIDNYGIH | WIWPYGGSTY | ARGYYWYDY |
| L2-52 | RASQDVNDGVA | SGRLGLYS | QQYFNWPIT | AASGFTIDNYGIH | WIWPYGGSTY | ARGYYWYDY |
| L2-53 | RASQDVNDGVA | SLPAGLYS | QQYFNWPIT | AASGFTIDNYGIH | WIWPYGGSTY | ARGYYWYDY |
| L2-54 | RASQDVNDGVA | SQALLLYS | QQYFNWPIT | AASGFTIDNYGIH | WIWPYGGSTY | ARGYYWYDY |
| L2-55 | RASQDVNDGVA | TGEVQLYS | QQYFNWPIT | AASGFTIDNYGIH | WIWPYGGSTY | ARGYYWYDY |
| L2-56 | RASQDVNDGVA | TMGGDLYS | QQYFNWPIT | AASGFTIDNYGIH | WIWPYGGSTY | ARGYYWYDY |
| L2-57 | RASQDVNDGVA | TNDGTLYS | QQYFNWPIT | AASGFTIDNYGIH | WIWPYGGSTY | ARGYYWYDY |
| L2-58 | RASQDVNDGVA | TNNNFLYS | QQYFNWPIT | AASGFTIDNYGIH | WIWPYGGSTY | ARGYYWYDY |
| L2-59 | RASQDVNDGVA | TRWDLLYS | QQYFNWPIT | AASGFTIDNYGIH | WIWPYGGSTY | ARGYYWYDY |
| L2-60 | RASQDVNDGVA | VGENELYS | QQYFNWPIT | AASGFTIDNYGIH | WIWPYGGSTY | ARGYYWYDY |
| L2-61 | RASQDVNDGVA | VRDGALYS | QQYFNWPIT | AASGFTIDNYGIH | WIWPYGGSTY | ARGYYWYDY |
| L2-62 | RASQDVNDGVA | WNGIKLYS | QQYFNWPIT | AASGFTIDNYGIH | WIWPYGGSTY | ARGYYWYDY |
| L2-63 | RASQDVNDGVA | WREGELYS | QQYFNWPIT | AASGFTIDNYGIH | WIWPYGGSTY | ARGYYWYDY |
| L3-01 | RASQDVNDGVA | SGSPWLYS | QQYFRWPVT | AASGFTIDNYGIH | WIWPYGGSTY | ARGYYWYDY |
| L3-02 | RASQDVNDGVA | SGSPWLYS | QQYYRWPTT | AASGFTIDNYGIH | WIWPYGGSTY | ARGYYWYDY |
| L3-03 | RASQDVNDGVA | SGSPWLYS | QQYYRWPVT | AASGFTIDNYGIH | WIWPYGGSTY | ARGYYWYDY |
| L3-04 | RASQDVNDGVA | SGSPWLYS | QQYWTWPTT | AASGFTIDNYGIH | WIWPYGGSTY | ARGYYWYDY |
| L3-05 | RASQDVNDGVA | SGSPWLYS | QQFYNWPST | AASGFTIDNYGIH | WIWPYGGSTY | ARGYYWYDY |
| L3-06 | RASQDVNDGVA | SGSPWLYS | QQYWKWPIT | AASGFTIDNYGIH | WIWPYGGSTY | ARGYYWYDY |
| L3-07 | RASQDVNDGVA | SGSPWLYS | QQYQRWPST | AASGFTIDNYGIH | WIWPYGGSTY | ARGYYWYDY |
| L3-08 | RASQDVNDGVA | SGSPWLYS | QQYWTWPAT | AASGFTIDNYGIH | WIWPYGGSTY | ARGYYWYDY |
| L3-09 | RASQDVNDGVA | SGSPWLYS | QQYYRWPST | AASGFTIDNYGIH | WIWPYGGSTY | ARGYYWYDY |
| L3-10 | RASQDVNDGVA | SGSPWLYS | QQYYRWPAT | AASGFTIDNYGIH | WIWPYGGSTY | ARGYYWYDY |
| L3-11 | RASQDVNDGVA | SGSPWLYS | QQFYPWPTT | AASGFTIDNYGIH | WIWPYGGSTY | ARGYYWYDY |
| L3-12 | RASQDVNDGVA | SGSPWLYS | QQFFQWPTT | AASGFTIDNYGIH | WIWPYGGSTY | ARGYYWYDY |
| L3-13 | RASQDVNDGVA | SGSPWLYS | QQYWNWPTT | AASGFTIDNYGIH | WIWPYGGSTY | ARGYYWYDY |
| L3-14 | RASQDVNDGVA | SGSPWLYS | QQYNRWPTT | AASGFTIDNYGIH | WIWPYGGSTY | ARGYYWYDY |
| L3-15 | RASQDVNDGVA | SGSPWLYS | QQYNTWPST | AASGFTIDNYGIH | WIWPYGGSTY | ARGYYWYDY |
| L3-16 | RASQDVNDGVA | SGSPWLYS | QQFYTWPVT | AASGFTIDNYGIH | WIWPYGGSTY | ARGYYWYDY |
| L3-17 | RASQDVNDGVA | SGSPWLYS | QQYFRWPTT | AASGFTIDNYGIH | WIWPYGGSTY | ARGYYWYDY |
| L3-18 | RASQDVNDGVA | SGSPWLYS | QQYWRWPAT | AASGFTIDNYGIH | WIWPYGGSTY | ARGYYWYDY |
| L3-19 | RASQDVNDGVA | SGSPWLYS | QQYNRWPVT | AASGFTIDNYGIH | WIWPYGGSTY | ARGYYWYDY |
| L3-20 | RASQDVNDGVA | SGSPWLYS | QQYWQWPVT | AASGFTIDNYGIH | WIWPYGGSTY | ARGYYWYDY |
| L3-21 | RASQDVNDGVA | SGSPWLYS | QQYQRWPTT | AASGFTIDNYGIH | WIWPYGGSTY | ARGYYWYDY |
| L3-22 | RASQDVNDGVA | SGSPWLYS | QQYQRWPVT | AASGFTIDNYGIH | WIWPYGGSTY | ARGYYWYDY |
| L3-23 | RASQDVNDGVA | SGSPWLYS | QQYSRWPTT | AASGFTIDNYGIH | WIWPYGGSTY | ARGYYWYDY |
| L3-24 | RASQDVNDGVA | SGSPWLYS | QQYWRWPVT | AASGFTIDNYGIH | WIWPYGGSTY | ARGYYWYDY |
| L3-25 | RASQDVNDGVA | SGSPWLYS | QQYARWPTT | AASGFTIDNYGIH | WIWPYGGSTY | ARGYYWYDY |
| L3-26 | RASQDVNDGVA | SGSPWLYS | QQYLRWPST | AASGFTIDNYGIH | WIWPYGGSTY | ARGYYWYDY |
| L3-27 | RASQDVNDGVA | SGSPWLYS | QQYIRWPVT | AASGFTIDNYGIH | WIWPYGGSTY | ARGYYWYDY |
| L3-28 | RASQDVNDGVA | SGSPWLYS | QQFYTWPST | AASGFTIDNYGIH | WIWPYGGSTY | ARGYYWYDY |
| L3-29 | RASQDVNDGVA | SGSPWLYS | QQFWRWPVT | AASGFTIDNYGIH | WIWPYGGSTY | ARGYYWYDY |
| L3-30 | RASQDVNDGVA | SGSPWLYS | QQYLRWPVT | AASGFTIDNYGIH | WIWPYGGSTY | ARGYYWYDY |
| L3-31 | RASQDVNDGVA | SGSPWLYS | QQYLRWPTT | AASGFTIDNYGIH | WIWPYGGSTY | ARGYYWYDY |
| L3-32 | RASQDVNDGVA | SGSPWLYS | QQYFRWPAT | AASGFTIDNYGIH | WIWPYGGSTY | ARGYYWYDY |
| H1-01 | RASQDVNDGVA | SGSPWLYS | QQYFNWPIT | AASGFTIRPMVIH | WIWPYGGSTY | ARGYYWYDY |
| H1-02 | RASQDVNDGVA | SGSPWLYS | QQYFNWPIT | AASGFTIAQTAIH | WIWPYGGSTY | ARGYYWYDY |
| H1-03 | RASQDVNDGVA | SGSPWLYS | QQYFNWPIT | AASGFTIDVPPIH | WIWPYGGSTY | ARGYYWYDY |
| H1-04 | RASQDVNDGVA | SGSPWLYS | QQYFNWPIT | AASGFTIPTTAIH | WIWPYGGSTY | ARGYYWYDY |
| H1-05 | RASQDVNDGVA | SGSPWLYS | QQYFNWPIT | AASGFTIQASSIH | WIWPYGGSTY | ARGYYWYDY |
| H1-06 | RASQDVNDGVA | SGSPWLYS | QQYFNWPIT | AASGFTISKSTIH | WIWPYGGSTY | ARGYYWYDY |
| H1-07 | RASQDVNDGVA | SGSPWLYS | QQYFNWPIT | AASGFTITPVPIH | WIWPYGGSTY | ARGYYWYDY |
| H1-08 | RASQDVNDGVA | SGSPWLYS | QQYFNWPIT | AASGFTIVYTSIH | WIWPYGGSTY | ARGYYWYDY |
| H1-09 | RASQDVNDGVA | SGSPWLYS | QQYFNWPIT | AASGFTIFRSAIH | WIWPYGGSTY | ARGYYWYDY |
| H1-10 | RASQDVNDGVA | SGSPWLYS | QQYFNWPIT | AASGFTIRQYTIH | WIWPYGGSTY | ARGYYWYDY |
| H1-11 | RASQDVNDGVA | SGSPWLYS | QQYFNWPIT | AASGFTIRMSTIH | WIWPYGGSTY | ARGYYWYDY |
| H1-12 | RASQDVNDGVA | SGSPWLYS | QQYFNWPIT | AASGFTIQRLPIH | WIWPYGGSTY | ARGYYWYDY |
| H1-13 | RASQDVNDGVA | SGSPWLYS | QQYFNWPIT | AASGFTIQQNTIH | WIWPYGGSTY | ARGYYWYDY |
| H1-14 | RASQDVNDGVA | SGSPWLYS | QQYFNWPIT | AASGFTIRHLPIH | WIWPYGGSTY | ARGYYWYDY |
| H1-15 | RASQDVNDGVA | SGSPWLYS | QQYFNWPIT | AASGFTINKAPIH | WIWPYGGSTY | ARGYYWYDY |
| H1-16 | RASQDVNDGVA | SGSPWLYS | QQYFNWPIT | AASGFTINQRSIH | WIWPYGGSTY | ARGYYWYDY |
| H1-17 | RASQDVNDGVA | SGSPWLYS | QQYFNWPIT | AASGFTIRGGTIH | WIWPYGGSTY | ARGYYWYDY |
| H1-18 | RASQDVNDGVA | SGSPWLYS | QQYFNWPIT | AASGFTIRSQPIH | WIWPYGGSTY | ARGYYWYDY |
| H1-19 | RASQDVNDGVA | SGSPWLYS | QQYFNWPIT | AASGFTIGKTTIH | WIWPYGGSTY | ARGYYWYDY |
| H1-20 | RASQDVNDGVA | SGSPWLYS | QQYFNWPIT | AASGFTIPSHVIH | WIWPYGGSTY | ARGYYWYDY |
| H1-21 | RASQDVNDGVA | SGSPWLYS | QQYFNWPIT | AASGFTITGARIH | WIWPYGGSTY | ARGYYWYDY |
| H1-22 | RASQDVNDGVA | SGSPWLYS | QQYFNWPIT | AASGFTIMAWPIH | WIWPYGGSTY | ARGYYWYDY |
| H1-23 | RASQDVNDGVA | SGSPWLYS | QQYFNWPIT | AASGFTIHRATIH | WIWPYGGSTY | ARGYYWYDY |
| H1-24 | RASQDVNDGVA | SGSPWLYS | QQYFNWPIT | AASGFTILYTTIH | WIWPYGGSTY | ARGYYWYDY |
| H1-25 | RASQDVNDGVA | SGSPWLYS | QQYFNWPIT | AASGFTIAARTIH | WIWPYGGSTY | ARGYYWYDY |
| H1-26 | RASQDVNDGVA | SGSPWLYS | QQYFNWPIT | AASGFTIQNLPIH | WIWPYGGSTY | ARGYYWYDY |
| H1-27 | RASQDVNDGVA | SGSPWLYS | QQYFNWPIT | AASGFTIPSVPIH | WIWPYGGSTY | ARGYYWYDY |
| H1-28 | RASQDVNDGVA | SGSPWLYS | QQYFNWPIT | AASGFTITKYTIH | WIWPYGGSTY | ARGYYWYDY |
| H1-29 | RASQDVNDGVA | SGSPWLYS | QQYFNWPIT | AASGFTINKGTIH | WIWPYGGSTY | ARGYYWYDY |
| H1-30 | RASQDVNDGVA | SGSPWLYS | QQYFNWPIT | AASGFTIASGPIH | WIWPYGGSTY | ARGYYWYDY |
| H1-31 | RASQDVNDGVA | SGSPWLYS | QQYFNWPIT | AASGFTIYRDRIH | WIWPYGGSTY | ARGYYWYDY |
| H1-32 | RASQDVNDGVA | SGSPWLYS | QQYFNWPIT | AASGFTIQRMPIH | WIWPYGGSTY | ARGYYWYDY |
| H1-33 | RASQDVNDGVA | SGSPWLYS | QQYFNWPIT | AASGFTIKPLPIH | WIWPYGGSTY | ARGYYWYDY |
| H1-34 | RASQDVNDGVA | SGSPWLYS | QQYFNWPIT | AASGFTIPIITIH | WIWPYGGSTY | ARGYYWYDY |
| H1-35 | RASQDVNDGVA | SGSPWLYS | QQYFNWPIT | AASGFTIQHHTIH | WIWPYGGSTY | ARGYYWYDY |
| H1-36 | RASQDVNDGVA | SGSPWLYS | QQYFNWPIT | AASGFTIESIPIH | WIWPYGGSTY | ARGYYWYDY |
| H1-37 | RASQDVNDGVA | SGSPWLYS | QQYFNWPIT | AASGFTILKSAIH | WIWPYGGSTY | ARGYYWYDY |
| H1-38 | RASQDVNDGVA | SGSPWLYS | QQYFNWPIT | AASGFTIDTRPIH | WIWPYGGSTY | ARGYYWYDY |
| H1-39 | RASQDVNDGVA | SGSPWLYS | QQYFNWPIT | AASGFTILGAPIH | WIWPYGGSTY | ARGYYWYDY |
| H1-40 | RASQDVNDGVA | SGSPWLYS | QQYFNWPIT | AASGFTIRRTGIH | WIWPYGGSTY | ARGYYWYDY |
| H1-41 | RASQDVNDGVA | SGSPWLYS | QQYFNWPIT | AASGFTIEMKPIH | WIWPYGGSTY | ARGYYWYDY |
| H1-42 | RASQDVNDGVA | SGSPWLYS | QQYFNWPIT | AASGFTISSRTIH | WIWPYGGSTY | ARGYYWYDY |
| H1-43 | RASQDVNDGVA | SGSPWLYS | QQYFNWPIT | AASGFTIRNRPIH | WIWPYGGSTY | ARGYYWYDY |
| H1-44 | RASQDVNDGVA | SGSPWLYS | QQYFNWPIT | AASGFTIQHVTIH | WIWPYGGSTY | ARGYYWYDY |
| H1-45 | RASQDVNDGVA | SGSPWLYS | QQYFNWPIT | AASGFTINRTPIH | WIWPYGGSTY | ARGYYWYDY |
| H1-46 | RASQDVNDGVA | SGSPWLYS | QQYFNWPIT | AASGFTISTLPIH | WIWPYGGSTY | ARGYYWYDY |
| H1-47 | RASQDVNDGVA | SGSPWLYS | QQYFNWPIT | AASGFTIWQRPIH | WIWPYGGSTY | ARGYYWYDY |
| H1-48 | RASQDVNDGVA | SGSPWLYS | QQYFNWPIT | AASGFTIFSSPIH | WIWPYGGSTY | ARGYYWYDY |
| H1-49 | RASQDVNDGVA | SGSPWLYS | QQYFNWPIT | AASGFTITANPIH | WIWPYGGSTY | ARGYYWYDY |
| H1-50 | RASQDVNDGVA | SGSPWLYS | QQYFNWPIT | AASGFTIGTGPIH | WIWPYGGSTY | ARGYYWYDY |
| H1-51 | RASQDVNDGVA | SGSPWLYS | QQYFNWPIT | AASGFTINSWPIH | WIWPYGGSTY | ARGYYWYDY |
| H1-52 | RASQDVNDGVA | SGSPWLYS | QQYFNWPIT | AASGFTIKEAPIH | WIWPYGGSTY | ARGYYWYDY |
| H1-53 | RASQDVNDGVA | SGSPWLYS | QQYFNWPIT | AASGFTIHEKPIH | WIWPYGGSTY | ARGYYWYDY |
| H1-54 | RASQDVNDGVA | SGSPWLYS | QQYFNWPIT | AASGFTIASKSIH | WIWPYGGSTY | ARGYYWYDY |
| H1-55 | RASQDVNDGVA | SGSPWLYS | QQYFNWPIT | AASGFTIAKTPIH | WIWPYGGSTY | ARGYYWYDY |
| H1-56 | RASQDVNDGVA | SGSPWLYS | QQYFNWPIT | AASGFTINHTPIH | WIWPYGGSTY | ARGYYWYDY |
| H1-57 | RASQDVNDGVA | SGSPWLYS | QQYFNWPIT | AASGFTISHLPIH | WIWPYGGSTY | ARGYYWYDY |
| H1-58 | RASQDVNDGVA | SGSPWLYS | QQYFNWPIT | AASGFTIARLPIH | WIWPYGGSTY | ARGYYWYDY |
| H1-59 | RASQDVNDGVA | SGSPWLYS | QQYFNWPIT | AASGFTITTMPIH | WIWPYGGSTY | ARGYYWYDY |
| H1-60 | RASQDVNDGVA | SGSPWLYS | QQYFNWPIT | AASGFTISPSPIH | WIWPYGGSTY | ARGYYWYDY |
| H1-61 | RASQDVNDGVA | SGSPWLYS | QQYFNWPIT | AASGFTIAHTPIH | WIWPYGGSTY | ARGYYWYDY |
| H2-01 | RASQDVNDGVA | SGSPWLYS | QQYFNWPIT | AASGFTIDNYGIH | SIFPRSGFTL | ARGYYWYDY |
| H2-02 | RASQDVNDGVA | SGSPWLYS | QQYFNWPIT | AASGFTIDNYGIH | QILPTKGFTF | ARGYYWYDY |
| H2-03 | RASQDVNDGVA | SGSPWLYS | QQYFNWPIT | AASGFTIDNYGIH | SIHPNKGFTL | ARGYYWYDY |
| H2-04 | RASQDVNDGVA | SGSPWLYS | QQYFNWPIT | AASGFTIDNYGIH | MIRPGRGWTM | ARGYYWYDY |
| H2-05 | RASQDVNDGVA | SGSPWLYS | QQYFNWPIT | AASGFTIDNYGIH | SIWPSRGYTT | ARGYYWYDY |
| H2-06 | RASQDVNDGVA | SGSPWLYS | QQYFNWPIT | AASGFTIDNYGIH | SIWPHKGFTF | ARGYYWYDY |
| H2-07 | RASQDVNDGVA | SGSPWLYS | QQYFNWPIT | AASGFTIDNYGIH | NILPKQGLTV | ARGYYWYDY |
| H2-08 | RASQDVNDGVA | SGSPWLYS | QQYFNWPIT | AASGFTIDNYGIH | LIWPVRGMTV | ARGYYWYDY |
| H2-09 | RASQDVNDGVA | SGSPWLYS | QQYFNWPIT | AASGFTIDNYGIH | SIRPKRGVTT | ARGYYWYDY |
| H2-10 | RASQDVNDGVA | SGSPWLYS | QQYFNWPIT | AASGFTIDNYGIH | SIRPLSGFTT | ARGYYWYDY |
| H2-11 | RASQDVNDGVA | SGSPWLYS | QQYFNWPIT | AASGFTIDNYGIH | QIRPVSGNTL | ARGYYWYDY |
| H2-12 | RASQDVNDGVA | SGSPWLYS | QQYFNWPIT | AASGFTIDNYGIH | LIWPWRGITF | ARGYYWYDY |
| H2-13 | RASQDVNDGVA | SGSPWLYS | QQYFNWPIT | AASGFTIDNYGIH | SIRPGSGFTL | ARGYYWYDY |
| H2-14 | RASQDVNDGVA | SGSPWLYS | QQYFNWPIT | AASGFTIDNYGIH | LISPRPGFTV | ARGYYWYDY |
| H2-15 | RASQDVNDGVA | SGSPWLYS | QQYFNWPIT | AASGFTIDNYGIH | SITPFRGFTL | ARGYYWYDY |
| H2-16 | RASQDVNDGVA | SGSPWLYS | QQYFNWPIT | AASGFTIDNYGIH | LIWPRNGHTY | ARGYYWYDY |
| H2-17 | RASQDVNDGVA | SGSPWLYS | QQYFNWPIT | AASGFTIDNYGIH | LIWPRNGVTL | ARGYYWYDY |
| H2-18 | RASQDVNDGVA | SGSPWLYS | QQYFNWPIT | AASGFTIDNYGIH | SIWPSAGITT | ARGYYWYDY |
| H2-19 | RASQDVNDGVA | SGSPWLYS | QQYFNWPIT | AASGFTIDNYGIH | GIFPHKGITL | ARGYYWYDY |
| H2-20 | RASQDVNDGVA | SGSPWLYS | QQYFNWPIT | AASGFTIDNYGIH | LIYPRPGVTI | ARGYYWYDY |
| H2-21 | RASQDVNDGVA | SGSPWLYS | QQYFNWPIT | AASGFTIDNYGIH | SIGPWRGVTM | ARGYYWYDY |
| H2-22 | RASQDVNDGVA | SGSPWLYS | QQYFNWPIT | AASGFTIDNYGIH | SIPPKTGLTR | ARGYYWYDY |
| H2-23 | RASQDVNDGVA | SGSPWLYS | QQYFNWPIT | AASGFTIDNYGIH | LIWPLSGATV | ARGYYWYDY |
| H2-24 | RASQDVNDGVA | SGSPWLYS | QQYFNWPIT | AASGFTIDNYGIH | NITPRMGLTM | ARGYYWYDY |
| H2-25 | RASQDVNDGVA | SGSPWLYS | QQYFNWPIT | AASGFTIDNYGIH | SIYPKAGFTL | ARGYYWYDY |
| H2-26 | RASQDVNDGVA | SGSPWLYS | QQYFNWPIT | AASGFTIDNYGIH | TILPRAGMTL | ARGYYWYDY |
| H2-27 | RASQDVNDGVA | SGSPWLYS | QQYFNWPIT | AASGFTIDNYGIH | TIPPRSGQTM | ARGYYWYDY |
| H2-28 | RASQDVNDGVA | SGSPWLYS | QQYFNWPIT | AASGFTIDNYGIH | SIRPLRGITN | ARGYYWYDY |
| H2-29 | RASQDVNDGVA | SGSPWLYS | QQYFNWPIT | AASGFTIDNYGIH | EIKPRVGWTL | ARGYYWYDY |
| H2-30 | RASQDVNDGVA | SGSPWLYS | QQYFNWPIT | AASGFTIDNYGIH | LIKPGVGTTV | ARGYYWYDY |
| H2-31 | RASQDVNDGVA | SGSPWLYS | QQYFNWPIT | AASGFTIDNYGIH | TIWPINGDTT | ARGYYWYDY |
| H2-32 | RASQDVNDGVA | SGSPWLYS | QQYFNWPIT | AASGFTIDNYGIH | LIFPRLGSTL | ARGYYWYDY |
| H2-33 | RASQDVNDGVA | SGSPWLYS | QQYFNWPIT | AASGFTIDNYGIH | QITPSKGWTS | ARGYYWYDY |
| H2-34 | RASQDVNDGVA | SGSPWLYS | QQYFNWPIT | AASGFTIDNYGIH | SIRPLHGFTF | ARGYYWYDY |
| H2-35 | RASQDVNDGVA | SGSPWLYS | QQYFNWPIT | AASGFTIDNYGIH | RINPSRGVTM | ARGYYWYDY |
| H2-36 | RASQDVNDGVA | SGSPWLYS | QQYFNWPIT | AASGFTIDNYGIH | LIPPTNGHTF | ARGYYWYDY |
| H2-37 | RASQDVNDGVA | SGSPWLYS | QQYFNWPIT | AASGFTIDNYGIH | SIWPNSGSTM | ARGYYWYDY |
| H2-38 | RASQDVNDGVA | SGSPWLYS | QQYFNWPIT | AASGFTIDNYGIH | GIWPTAGATL | ARGYYWYDY |
| H2-39 | RASQDVNDGVA | SGSPWLYS | QQYFNWPIT | AASGFTIDNYGIH | LIWPRSGITM | ARGYYWYDY |
| H2-40 | RASQDVNDGVA | SGSPWLYS | QQYFNWPIT | AASGFTIDNYGIH | SIWPFRGFTT | ARGYYWYDY |
| H2-41 | RASQDVNDGVA | SGSPWLYS | QQYFNWPIT | AASGFTIDNYGIH | SIWPRRGFTM | ARGYYWYDY |
| H2-42 | RASQDVNDGVA | SGSPWLYS | QQYFNWPIT | AASGFTIDNYGIH | LIWPFKGYTI | ARGYYWYDY |
| H2-43 | RASQDVNDGVA | SGSPWLYS | QQYFNWPIT | AASGFTIDNYGIH | TIWPDRGYTL | ARGYYWYDY |
| H2-44 | RASQDVNDGVA | SGSPWLYS | QQYFNWPIT | AASGFTIDNYGIH | LIRPIRGHTV | ARGYYWYDY |
| H2-45 | RASQDVNDGVA | SGSPWLYS | QQYFNWPIT | AASGFTIDNYGIH | TIWPSSGSTS | ARGYYWYDY |
| H2-46 | RASQDVNDGVA | SGSPWLYS | QQYFNWPIT | AASGFTIDNYGIH | SISPRQGVTM | ARGYYWYDY |
| H2-47 | RASQDVNDGVA | SGSPWLYS | QQYFNWPIT | AASGFTIDNYGIH | LIWPHMGRTI | ARGYYWYDY |
| H2-48 | RASQDVNDGVA | SGSPWLYS | QQYFNWPIT | AASGFTIDNYGIH | VIWPKRGSTV | ARGYYWYDY |
| H2-49 | RASQDVNDGVA | SGSPWLYS | QQYFNWPIT | AASGFTIDNYGIH | LIHPLRGATI | ARGYYWYDY |
| H2-50 | RASQDVNDGVA | SGSPWLYS | QQYFNWPIT | AASGFTIDNYGIH | AIWPRSGITL | ARGYYWYDY |
| H2-51 | RASQDVNDGVA | SGSPWLYS | QQYFNWPIT | AASGFTIDNYGIH | SINPFRGFTL | ARGYYWYDY |
| H2-52 | RASQDVNDGVA | SGSPWLYS | QQYFNWPIT | AASGFTIDNYGIH | SIYPGRGVTS | ARGYYWYDY |
| H2-53 | RASQDVNDGVA | SGSPWLYS | QQYFNWPIT | AASGFTIDNYGIH | SIWPDQGITT | ARGYYWYDY |
| H2-54 | RASQDVNDGVA | SGSPWLYS | QQYFNWPIT | AASGFTIDNYGIH | SIWPRLGITT | ARGYYWYDY |
| H2-55 | RASQDVNDGVA | SGSPWLYS | QQYFNWPIT | AASGFTIDNYGIH | SIWPRGGVTM | ARGYYWYDY |
| H2-56 | RASQDVNDGVA | SGSPWLYS | QQYFNWPIT | AASGFTIDNYGIH | SIFPYRGMTL | ARGYYWYDY |
| H2-57 | RASQDVNDGVA | SGSPWLYS | QQYFNWPIT | AASGFTIDNYGIH | SITPYRGCTF | ARGYYWYDY |
| H2-58 | RASQDVNDGVA | SGSPWLYS | QQYFNWPIT | AASGFTIDNYGIH | LILPRAGVTT | ARGYYWYDY |
| H2-59 | RASQDVNDGVA | SGSPWLYS | QQYFNWPIT | AASGFTIDNYGIH | SITPKIGMTF | ARGYYWYDY |
| H2-60 | RASQDVNDGVA | SGSPWLYS | QQYFNWPIT | AASGFTIDNYGIH | GIYPTRGMTV | ARGYYWYDY |
| H2-61 | RASQDVNDGVA | SGSPWLYS | QQYFNWPIT | AASGFTIDNYGIH | SIHPFRGVTL | ARGYYWYDY |
| H2-62 | RASQDVNDGVA | SGSPWLYS | QQYFNWPIT | AASGFTIDNYGIH | SIYPTRGMTL | ARGYYWYDY |
| H2-63 | RASQDVNDGVA | SGSPWLYS | QQYFNWPIT | AASGFTIDNYGIH | LIMPRKGFTA | ARGYYWYDY |
| H2-64 | RASQDVNDGVA | SGSPWLYS | QQYFNWPIT | AASGFTIDNYGIH | SIWPRKGVTL | ARGYYWYDY |
| H2-65 | RASQDVNDGVA | SGSPWLYS | QQYFNWPIT | AASGFTIDNYGIH | SIYPRQGITT | ARGYYWYDY |
| H2-66 | RASQDVNDGVA | SGSPWLYS | QQYFNWPIT | AASGFTIDNYGIH | AIFPVMGSTL | ARGYYWYDY |
| H2-67 | RASQDVNDGVA | SGSPWLYS | QQYFNWPIT | AASGFTIDNYGIH | AIFPFGGKTT | ARGYYWYDY |
| H2-68 | RASQDVNDGVA | SGSPWLYS | QQYFNWPIT | AASGFTIDNYGIH | SIKPSYGYTL | ARGYYWYDY |
| H2-69 | RASQDVNDGVA | SGSPWLYS | QQYFNWPIT | AASGFTIDNYGIH | WIRPRQGGTH | ARGYYWYDY |
| H2-70 | RASQDVNDGVA | SGSPWLYS | QQYFNWPIT | AASGFTIDNYGIH | TIWPTNGSTM | ARGYYWYDY |
| H2-71 | RASQDVNDGVA | SGSPWLYS | QQYFNWPIT | AASGFTIDNYGIH | AIWPQKGITT | ARGYYWYDY |
| H2-72 | RASQDVNDGVA | SGSPWLYS | QQYFNWPIT | AASGFTIDNYGIH | SITPRLGLTM | ARGYYWYDY |
| H2-73 | RASQDVNDGVA | SGSPWLYS | QQYFNWPIT | AASGFTIDNYGIH | DIRPVNGITM | ARGYYWYDY |
| H2-74 | RASQDVNDGVA | SGSPWLYS | QQYFNWPIT | AASGFTIDNYGIH | SIWPSTGLTL | ARGYYWYDY |
| H2-75 | RASQDVNDGVA | SGSPWLYS | QQYFNWPIT | AASGFTIDNYGIH | SINPSYGYTM | ARGYYWYDY |
| H2-76 | RASQDVNDGVA | SGSPWLYS | QQYFNWPIT | AASGFTIDNYGIH | QIKPSWGHTL | ARGYYWYDY |
| H2-77 | RASQDVNDGVA | SGSPWLYS | QQYFNWPIT | AASGFTIDNYGIH | LIRPLMGNTI | ARGYYWYDY |
| H2-78 | RASQDVNDGVA | SGSPWLYS | QQYFNWPIT | AASGFTIDNYGIH | AIFPFGGKTS | ARGYYWYDY |
| H3-01 | RASQDVNDGVA | SGSPWLYS | QQYFNWPIT | AASGFTIDNYGIH | WIWPYGGSTY | ARGYYWYDY |
| H3-02 | RASQDVNDGVA | SGSPWLYS | QQYFNWPIT | AASGFTIDNYGIH | WIWPYGGSTY | ARGYFWYDY |
| H3-03 | RASQDVNDGVA | SGSPWLYS | QQYFNWPIT | AASGFTIDNYGIH | WIWPYGGSTY | ARCYPLMDY |
| H3-04 | RASQDVNDGVA | SGSPWLYS | QQYFNWPIT | AASGFTIDNYGIH | WIWPYGGSTY | ARGRFWYDY |
| H3-05 | RASQDVNDGVA | SGSPWLYS | QQYFNWPIT | AASGFTIDNYGIH | WIWPYGGSTY | ARGRYFLDY |
| H3-06 | RASQDVNDGVA | SGSPWLYS | QQYFNWPIT | AASGFTIDNYGIH | WIWPYGGSTY | ARGLYWQDY |
| H3-07 | RASQDVNDGVA | SGSPWLYS | QQYFNWPIT | AASGFTIDNYGIH | WIWPYGGSTY | ARGRYLYDY |
| H3-08 | RASQDVNDGVA | SGSPWLYS | QQYFNWPIT | AASGFTIDNYGIH | WIWPYGGSTY | ARARFWLDY |
| H3-09 | RASQDVNDGVA | SGSPWLYS | QQYFNWPIT | AASGFTIDNYGIH | WIWPYGGSTY | ARGRYWMDY |
| H3-10 | RASQDVNDGVA | SGSPWLYS | QQYFNWPIT | AASGFTIDNYGIH | WIWPYGGSTY | ARGFYWLDY |
| H3-11 | RASQDVNDGVA | SGSPWLYS | QQYFNWPIT | AASGFTIDNYGIH | WIWPYGGSTY | ARGSYWLDY |
| H3-12 | RASQDVNDGVA | SGSPWLYS | QQYFNWPIT | AASGFTIDNYGIH | WIWPYGGSTY | ARGRYWYDY |
| H3-13 | RASQDVNDGVA | SGSPWLYS | QQYFNWPIT | AASGFTIDNYGIH | WIWPYGGSTY | ARGRYWLDY |
| H3-14 | RASQDVNDGVA | SGSPWLYS | QQYFNWPIT | AASGFTIDNYGIH | WIWPYGGSTY | ARGRYYYDY |
| H3-15 | RASQDVNDGVA | SGSPWLYS | QQYFNWPIT | AASGFTIDNYGIH | WIWPYGGSTY | ARGRYWIDY |
| H3-16 | RASQDVNDGVA | SGSPWLYS | QQYFNWPIT | AASGFTIDNYGIH | WIWPYGGSTY | ARGRFWLDY |
| H3-17 | RASQDVNDGVA | SGSPWLYS | QQYFNWPIT | AASGFTIDNYGIH | WIWPYGGSTY | ARGRYIYDY |
| H3-18 | RASQDVNDGVA | SGSPWLYS | QQYFNWPIT | AASGFTIDNYGIH | WIWPYGGSTY | ARARYWLDY |
| H3-19 | RASQDVNDGVA | SGSPWLYS | QQYFNWPIT | AASGFTIDNYGIH | WIWPYGGSTY | ARGRYFYDY |
| H3-20 | RASQDVNDGVA | SGSPWLYS | QQYFNWPIT | AASGFTIDNYGIH | WIWPYGGSTY | ARGSYWYDY |
| H3-21 | RASQDVNDGVA | SGSPWLYS | QQYFNWPIT | AASGFTIDNYGIH | WIWPYGGSTY | ARGRYWLDY |
| H3-22 | RASQDVNDGVA | SGSPWLYS | QQYFNWPIT | AASGFTIDNYGIH | WIWPYGGSTY | ARGAYWYDY |
| H3-23 | RASQDVNDGVA | SGSPWLYS | QQYFNWPIT | AASGFTIDNYGIH | WIWPYGGSTY | ARGYYWYDY |
| H3-24 | RASQDVNDGVA | SGSPWLYS | QQYFNWPIT | AASGFTIDNYGIH | WIWPYGGSTY | ARGLYWYDY |
| H3-25 | RASQDVNDGVA | SGSPWLYS | QQYFNWPIT | AASGFTIDNYGIH | WIWPYGGSTY | ARGAYWLDY |
| H3-26 | RASQDVNDGVA | SGSPWLYS | QQYFNWPIT | AASGFTIDNYGIH | WIWPYGGSTY | ARGRYFYDY |
| H3-27 | RASQDVNDGVA | SGSPWLYS | QQYFNWPIT | AASGFTIDNYGIH | WIWPYGGSTY | ARGLYWLDY |
| H3-28 | RASQDVNDGVA | SGSPWLYS | QQYFNWPIT | AASGFTIDNYGIH | WIWPYGGSTY | ARGSYWFDY |
| H3-29 | RASQDVNDGVA | SGSPWLYS | QQYFNWPIT | AASGFTIDNYGIH | WIWPYGGSTY | ARGLYWFDY |
| H3-30 | RASQDVNDGVA | SGSPWLYS | QQYFNWPIT | AASGFTIDNYGIH | WIWPYGGSTY | ARGRFWYEY |
| H3-31 | RASQDVNDGVA | SGSPWLYS | QQYFNWPIT | AASGFTIDNYGIH | WIWPYGGSTY | ARGGYWFDY |
| H3-32 | RASQDVNDGVA | SGSPWLYS | QQYFNWPIT | AASGFTIDNYGIH | WIWPYGGSTY | ARGLYWVDY |
| H3-33 | RASQDVNDGVA | SGSPWLYS | QQYFNWPIT | AASGFTIDNYGIH | WIWPYGGSTY | ARGSYWLEY |
| H3-34 | RASQDVNDGVA | SGSPWLYS | QQYFNWPIT | AASGFTIDNYGIH | WIWPYGGSTY | ARDRYWYEY |
| H3-35 | RASQDVNDGVA | SGSPWLYS | QQYFNWPIT | AASGFTIDNYGIH | WIWPYGGSTY | ARGHYWHDY |
| H3-36 | RASQDVNDGVA | SGSPWLYS | QQYFNWPIT | AASGFTIDNYGIH | WIWPYGGSTY | AREMYVHDY |
| H3-37 | RASQDVNDGVA | SGSPWLYS | QQYFNWPIT | AASGFTIDNYGIH | WIWPYGGSTY | ARGRYWYDY |
| H3-38 | RASQDVNDGVA | SGSPWLYS | QQYFNWPIT | AASGFTIDNYGIH | WIWPYGGSTY | ARGRYFFDY |

**Supplementary Table S5, related to Figure 5**. CDR variant sequences of P06. The sequence ranges of the CDR residue positions are labelled in the first row by the IMGT numbering.

| Name | **L1(29~39)** | **L2(54~69)** | **L3(105~118)** | **H1(29~40)** | **H2(55~67)** | **H3(105~117)** |
| --- | --- | --- | --- | --- | --- | --- |
| P06 | VGGGV | ISGTSGLYS | QQSSNFPITF | TIGGYWIH | GIGPYWGSTYY | ARFNNWFWNVMDY |
| H1-01A | VGGGV | ISGTSGLYS | QQSSNFPITF | TIKPYEIH | GIGPYWGSTYY | ARFNNWFWNVMDY |
| H1-01B | VGGGV | ISGTSGLYS | QQSSNFPITF | TIRGMWIH | GIGPYWGSTYY | ARFNNWFWNVMDY |
| H1-01E | VGGGV | ISGTSGLYS | QQSSNFPITF | TINSYYIH | GIGPYWGSTYY | ARFNNWFWNVMDY |
| H1-03A | VGGGV | ISGTSGLYS | QQSSNFPITF | TIGSMWIH | GIGPYWGSTYY | ARFNNWFWNVMDY |
| H1-03D | VGGGV | ISGTSGLYS | QQSSNFPITF | TINSMWIH | GIGPYWGSTYY | ARFNNWFWNVMDY |
| H1-04A | VGGGV | ISGTSGLYS | QQSSNFPITF | TINSMWIH | GIGPYWGSTYY | ARFNNWFWNVMDY |
| H1-05B | VGGGV | ISGTSGLYS | QQSSNFPITF | TISSMWIH | GIGPYWGSTYY | ARFNNWFWNVMDY |
| H1-06A | VGGGV | ISGTSGLYS | QQSSNFPITF | TISSMWIH | GIGPYWGSTYY | ARFNNWFWNVMDY |
| H1-06D | VGGGV | ISGTSGLYS | QQSSNFPITF | TIRSMYIH | GIGPYWGSTYY | ARFNNWFWNVMDY |
| H1-06H | VGGGV | ISGTSGLYS | QQSSNFPITF | TISGYWIH | GIGPYWGSTYY | ARFNNWFWNVMDY |
| H1-07A | VGGGV | ISGTSGLYS | QQSSNFPITF | TISSYTIH | GIGPYWGSTYY | ARFNNWFWNVMDY |
| H1-07B | VGGGV | ISGTSGLYS | QQSSNFPITF | TIGCMWIH | GIGPYWGSTYY | ARFNNWFWNVMDY |
| H1-07D | VGGGV | ISGTSGLYS | QQSSNFPITF | TIRSMYIH | GIGPYWGSTYY | ARFNNWFWNVMDY |
| H1-08A | VGGGV | ISGTSGLYS | QQSSNFPITF | TIGSMWIH | GIGPYWGSTYY | ARFNNWFWNVMDY |
| H1-08B | VGGGV | ISGTSGLYS | QQSSNFPITF | TINSMWIH | GIGPYWGSTYY | ARFNNWFWNVMDY |
| H1-08C | VGGGV | ISGTSGLYS | QQSSNFPITF | TIPAYEIH | GIGPYWGSTYY | ARFNNWFWNVMDY |
| H1-08D | VGGGV | ISGTSGLYS | QQSSNFPITF | TIGGYMIH | GIGPYWGSTYY | ARFNNWFWNVMDY |
| H1-10A | VGGGV | ISGTSGLYS | QQSSNFPITF | TIGSMWIH | GIGPYWGSTYY | ARFNNWFWNVMDY |
| H1-10B | VGGGV | ISGTSGLYS | QQSSNFPITF | TIGSMWIH | GIGPYWGSTYY | ARFNNWFWNVMDY |
| H1-10C | VGGGV | ISGTSGLYS | QQSSNFPITF | TIRSMYIH | GIGPYWGSTYY | ARFNNWFWNVMDY |
| H1-11A | VGGGV | ISGTSGLYS | QQSSNFPITF | TIGSMWIH | GIGPYWGSTYY | ARFNNWFWNVMDY |
| H1-11B | VGGGV | ISGTSGLYS | QQSSNFPITF | TISSYWIH | GIGPYWGSTYY | ARFNNWFWNVMDY |
| H1-11C | VGGGV | ISGTSGLYS | QQSSNFPITF | TIGSMWIH | GIGPYWGSTYY | ARFNNWFWNVMDY |
| H1-11D | VGGGV | ISGTSGLYS | QQSSNFPITF | TIGSMWIH | GIGPYWGSTYY | ARFNNWFWNVMDY |
| 21-01A | VGGGV | ISGTSGLYS | QQSSNFPITF | TIGSMWIH | GIGPYWGSTYY | ARFNNWFWNVMDY |
| 21-01C | VGGGV | ISGTSGLYS | QQSSNFPITF | TIGSMWIH | GIGPYWGSTYY | ARFNNWFWNVMDY |
| 21-02A | VGGGV | ISGTSGLYS | QQSSNFPITF | TIGSMWIH | GIGPYWGSTYY | ARFNNWFWNVMDY |
| 21-02B | VGGGV | ISGTSGLYS | QQSSNFPITF | TIGSMWIH | GIGPYWGSTYY | ARFNNWFWNVMDY |
| 21-02F | VGGGV | ISGTSGLYS | QQSSNFPITF | TISSYWIH | GIGPYWGSTYY | ARFNNWFWNVMDY |
| 21-02G | VGGGV | ISGTSGLYS | QQSSNFPITF | TIGSMWIH | GIGPYWGSTYY | ARFNNWFWNVMDY |
| 21-02H | VGGGV | ISGTSGLYS | QQSSNFPITF | TIGSMWIH | GIGPYWGSTYY | ARFNNWFWNVMDY |
| 21-03A | VGGGV | ISGTSGLYS | QQSSNFPITF | TIGSMWIH | GIGPYWGSTYY | ARFNNWFWNVMDY |
| 21-03B | VGGGV | ISGTSGLYS | QQSSNFPITF | TIRSMYIH | GIGPYWGSTYY | ARFNNWFWNVMDY |
| 21-03D | VGGGV | ISGTSGLYS | QQSSNFPITF | TIGSMWIH | GIGPYWGSTYY | ARFNNWFWNVMDY |
| 21-03E | VGGGV | ISGTSGLYS | QQSSNFPITF | TIGSMWIH | GIGPYWGSTYY | ARFNNWFWNVMDY |
| 21-03H | VGGGV | ISGTSGLYS | QQSSNFPITF | TINSMWIH | GIGPYWGSTYY | ARFNNWFWNVMDY |
| 21-04E | VGGGV | ISGTSGLYS | QQSSNFPITF | TINSMWIH | GIGPYWGSTYY | ARFNNWFWNVMDY |
| 21-05A | VGGGV | ISGTSGLYS | QQSSNFPITF | TINSMWIH | GIGPYWGSTYY | ARFNNWFWNVMDY |
| 21-05C | VGGGV | ISGTSGLYS | QQSSNFPITF | TINSMWIH | GIGPYWGSTYY | ARFNNWFWNVMDY |
| 21-05F | VGGGV | ISGTSGLYS | QQSSNFPITF | TISSMWIH | GIGPYWGSTYY | ARFNNWFWNVMDY |
| 21-06C | VGGGV | ISGTSGLYS | QQSSNFPITF | TIGSMWIH | GIGPYWGSTYY | ARFNNWFWNVMDY |
| 21-06D | VGGGV | ISGTSGLYS | QQSSNFPITF | TIV*SPIH | GIGPYWGSTYY | ARFNNWFWNVMDY |
| 21-07B | VGGGV | ISGTSGLYS | QQSSNFPITF | TIRSMYIH | GIGPYWGSTYY | ARFNNWFWNVMDY |
| 21-07D | VGGGV | ISGTSGLYS | QQSSNFPITF | TIGSMWIH | GIGPYWGSTYY | ARFNNWFWNVMDY |
| 21-07E | VGGGV | ISGTSGLYS | QQSSNFPITF | TIRSMYIH | GIGPYWGSTYY | ARFNNWFWNVMDY |
| 21-08A | VGGGV | ISGTSGLYS | QQSSNFPITF | TIKMKPIH | GIGPYWGSTYY | ARFNNWFWNVMDY |
| 21-08C | VGGGV | ISGTSGLYS | QQSSNFPITF | TINSMWIH | GIGPYWGSTYY | ARFNNWFWNVMDY |
| 21-08H | VGGGV | ISGTSGLYS | QQSSNFPITF | TIGSMWIH | GIGPYWGSTYY | ARFNNWFWNVMDY |
| 21-09A | VGGGV | ISGTSGLYS | QQSSNFPITF | TIRSMYIH | GIGPYWGSTYY | ARFNNWFWNVMDY |
| 21-09D | VGGGV | ISGTSGLYS | QQSSNFPITF | TIGSMWIH | GIGPYWGSTYY | ARFNNWFWNVMDY |
| 21-09E | VGGGV | ISGTSGLYS | QQSSNFPITF | TIGSMWIH | GIGPYWGSTYY | ARFNNWFWNVMDY |
| 21-10B | VGGGV | ISGTSGLYS | QQSSNFPITF | TISSMWIH | GIGPYWGSTYY | ARFNNWFWNVMDY |
| 21-10C | VGGGV | ISGTSGLYS | QQSSNFPITF | TIGSMWIH | GIGPYWGSTYY | ARFNNWFWNVMDY |
| 21-10H | VGGGV | ISGTSGLYS | QQSSNFPITF | TIGSMWIH | GIGPYWGSTYY | ARFNNWFWNVMDY |
| 21-11B | VGGGV | ISGTSGLYS | QQSSNFPITF | TIGSMWIH | GIGPYWGSTYY | ARFNNWFWNVMDY |
| 21-11E | VGGGV | ISGTSGLYS | QQSSNFPITF | TISSMWIH | GIGPYWGSTYY | ARFNNWFWNVMDY |
| 21-11F | VGGGV | ISGTSGLYS | QQSSNFPITF | TIRGMWIH | GIGPYWGSTYY | ARFNNWFWNVMDY |
| 21-11G | VGGGV | ISGTSGLYS | QQSSNFPITF | TISSMWIH | GIGPYWGSTYY | ARFNNWFWNVMDY |
| 21-12C | VGGGV | ISGTSGLYS | QQSSNFPITF | TISSYTIH | GIGPYWGSTYY | ARFNNWFWNVMDY |
| 21-12D | VGGGV | ISGTSGLYS | QQSSNFPITF | TISSMWIH | GIGPYWGSTYY | ARFNNWFWNVMDY |
| 21-12F | VGGGV | ISGTSGLYS | QQSSNFPITF | TIGSMWIH | GIGPYWGSTYY | ARFNNWFWNVMDY |
| 21-12G | VGGGV | ISGTSGLYS | QQSSNFPITF | TIGSMWIH | GIGPYWGSTYY | ARFNNWFWNVMDY |
| 22-01A | VGGGV | ISGTSGLYS | QQSSNFPITF | TIGSMWIH | GIGPYWGSTYY | ARFNNWFWNVMDY |
| 22-01B | VGGGV | ISGTSGLYS | QQSSNFPITF | TISSMWIH | GIGPYWGSTYY | ARFNNWFWNVMDY |
| 22-01C | VGGGV | ISGTSGLYS | QQSSNFPITF | TIPSFWIH | GIGPYWGSTYY | ARFNNWFWNVMDY |
| 22-01E | VGGGV | ISGTSGLYS | QQSSNFPITF | TIGSMWIH | GIGPYWGSTYY | ARFNNWFWNVMDY |
| 22-01F | VGGGV | ISGTSGLYS | QQSSNFPITF | TIHSMYIH | GIGPYWGSTYY | ARFNNWFWNVMDY |
| 22-01G | VGGGV | ISGTSGLYS | QQSSNFPITF | TINSMWIH | GIGPYWGSTYY | ARFNNWFWNVMDY |
| 22-02A | VGGGV | ISGTSGLYS | QQSSNFPITF | TIGSMWIH | GIGPYWGSTYY | ARFNNWFWNVMDY |
| 22-02B | VGGGV | ISGTSGLYS | QQSSNFPITF | TIGSMWIH | GIGPYWGSTYY | ARFNNWFWNVMDY |
| 22-02C | VGGGV | ISGTSGLYS | QQSSNFPITF | TIGSMWIH | GIGPYWGSTYY | ARFNNWFWNVMDY |
| 22-02D | VGGGV | ISGTSGLYS | QQSSNFPITF | TIGSMWIH | GIGPYWGSTYY | ARFNNWFWNVMDY |
| 22-02E | VGGGV | ISGTSGLYS | QQSSNFPITF | TIRSMYIH | GIGPYWGSTYY | ARFNNWFWNVMDY |
| 22-02H | VGGGV | ISGTSGLYS | QQSSNFPITF | TIRSMYIH | GIGPYWGSTYY | ARFNNWFWNVMDY |
| 22-03A | VGGGV | ISGTSGLYS | QQSSNFPITF | TINGYSIH | GIGPYWGSTYY | ARFNNWFWNVMDY |
| 22-03B | VGGGV | ISGTSGLYS | QQSSNFPITF | TIRSMYIH | GIGPYWGSTYY | ARFNNWFWNVMDY |
| 22-03C | VGGGV | ISGTSGLYS | QQSSNFPITF | TIGSMWIH | GIGPYWGSTYY | ARFNNWFWNVMDY |
| 22-03E | VGGGV | ISGTSGLYS | QQSSNFPITF | TIRSMYIH | GIGPYWGSTYY | ARFNNWFWNVMDY |
| 22-03G | VGGGV | ISGTSGLYS | QQSSNFPITF | TINSMWIH | GIGPYWGSTYY | ARFNNWFWNVMDY |
| 22-03H | VGGGV | ISGTSGLYS | QQSSNFPITF | TIGSMWIH | GIGPYWGSTYY | ARFNNWFWNVMDY |
| H2-01A | VGGGV | ISGTSGLYS | QQSSNFPITF | TIGGYWIH | AIGPFLVRPGY | ARFNNWFWNVMDY |
| H2-01C | VGGGV | ISGTSGLYS | QQSSNFPITF | TIGGYWIH | AIGPFFGTTMY | ARFNNWFWNVMDY |
| H2-01D | VGGGV | ISGTSGLYS | QQSSNFPITF | TIGGYWIH | AIGPFFGTTMY | ARFNNWFWNVMDY |
| H2-01H | VGGGV | ISGTSGLYS | QQSSNFPITF | TIGGYWIH | GIGPWWGRTRY | ARFNNWFWNVMDY |
| H2-04D | VGGGV | ISGTSGLYS | QQSSNFPITF | TIGGYWIH | AIGPFFGTTMY | ARFNNWFWNVMDY |
| H2-05A | VGGGV | ISGTSGLYS | QQSSNFPITF | TIGGYWIH | AIAPWWGDTPY | ARFNNWFWNVMDY |
| H2-05B | VGGGV | ISGTSGLYS | QQSSNFPITF | TIGGYWIH | GIGPFFGPTRY | ARFNNWFWNVMDY |
| H2-05C | VGGGV | ISGTSGLYS | QQSSNFPITF | TIGGYWIH | GIGPFFGPTRY | ARFNNWFWNVMDY |
| H2-06C | VGGGV | ISGTSGLYS | QQSSNFPITF | TIGGYWIH | GIGPFFGPTRY | ARFNNWFWNVMDY |
| H2-07A | VGGGV | ISGTSGLYS | QQSSNFPITF | TIGGYWIH | AIGPFFGTTMY | ARFNNWFWNVMDY |
| H2-07B | VGGGV | ISGTSGLYS | QQSSNFPITF | TIGGYWIH | GIGPFFGPTRY | ARFNNWFWNVMDY |
| H2-07C | VGGGV | ISGTSGLYS | QQSSNFPITF | TIGGYWIH | AIGPFFGTTMY | ARFNNWFWNVMDY |
| H2-08A | VGGGV | ISGTSGLYS | QQSSNFPITF | TIGGYWIH | GIPPFYGTTRY | ARFNNWFWNVMDY |
| H2-08B | VGGGV | ISGTSGLYS | QQSSNFPITF | TIGGYWIH | GIGPFFGPTRY | ARFNNWFWNVMDY |
| H2-08D | VGGGV | ISGTSGLYS | QQSSNFPITF | TIGGYWIH | GIGPFFGPTRY | ARFNNWFWNVMDY |
| H2-09A | VGGGV | ISGTSGLYS | QQSSNFPITF | TIGGYWIH | AIGPFFGTTMY | ARFNNWFWNVMDY |
| H2-09B | VGGGV | ISGTSGLYS | QQSSNFPITF | TIGGYWIH | GIGPFFGPTRY | ARFNNWFWNVMDY |
| H2-09C | VGGGV | ISGTSGLYS | QQSSNFPITF | TIGGYWIH | AIGPFFGTTMY | ARFNNWFWNVMDY |
| H2-09D | VGGGV | ISGTSGLYS | QQSSNFPITF | TIGGYWIH | AIGPFFGTTMY | ARFNNWFWNVMDY |
| H2-09H | VGGGV | ISGTSGLYS | QQSSNFPITF | TIGGYWIH | TIGPYWGETIY | ARFNNWFWNVMDY |
| H2-10A | VGGGV | ISGTSGLYS | QQSSNFPITF | TIGGYWIH | AIGPFFGTTMY | ARFNNWFWNVMDY |
| H2-10B | VGGGV | ISGTSGLYS | QQSSNFPITF | TIGGYWIH | AIGPFFGTTMY | ARFNNWFWNVMDY |
| H2-10C | VGGGV | ISGTSGLYS | QQSSNFPITF | TIGGYWIH | GIQPLFGYTEY | ARFNNWFWNVMDY |
| H2-11A | VGGGV | ISGTSGLYS | QQSSNFPITF | TIGGYWIH | AIGPFFGTTMY | ARFNNWFWNVMDY |
| H2-11B | VGGGV | ISGTSGLYS | QQSSNFPITF | TIGGYWIH | GIGPFFGPTRY | ARFNNWFWNVMDY |
| H2-11C | VGGGV | ISGTSGLYS | QQSSNFPITF | TIGGYWIH | AIGPFFGTTMY | ARFNNWFWNVMDY |
| H2-11D | VGGGV | ISGTSGLYS | QQSSNFPITF | TIGGYWIH | GIGPFFGPTRY | ARFNNWFWNVMDY |
| H2-12A | VGGGV | ISGTSGLYS | QQSSNFPITF | TIGGYWIH | AIGPFFGTTMY | ARFNNWFWNVMDY |
| H2-12B | VGGGV | ISGTSGLYS | QQSSNFPITF | TIGGYWIH | GIGPFFGPTRY | ARFNNWFWNVMDY |
| H2-12C | VGGGV | ISGTSGLYS | QQSSNFPITF | TIGGYWIH | GIGPFFGPTTY | ARFNNWFWNVMDY |
| H2-12D | VGGGV | ISGTSGLYS | QQSSNFPITF | TIGGYWIH | AIGPFFGTTMY | ARFNNWFWNVMDY |
| 23-02F | VGGGV | ISGTSGLYS | QQSSNFPITF | TIGGYWIH | AIGPFFGTTMY | ARFNNWFWNVMDY |
| 23-03B | VGGGV | ISGTSGLYS | QQSSNFPITF | TIGGYWIH | GIGPWYGKTHY | ARFNNWFWNVMDY |
| 23-03F | VGGGV | ISGTSGLYS | QQSSNFPITF | TIGGYWIH | AIGPFFGTTMY | ARFNNWFWNVMDY |
| 23-05C | VGGGV | ISGTSGLYS | QQSSNFPITF | TIGGYWIH | GIPPFYGTTRY | ARFNNWFWNVMDY |
| 23-05D | VGGGV | ISGTSGLYS | QQSSNFPITF | TIGGYWIH | GIPPFYGTTRY | ARFNNWFWNVMDY |
| 23-06C | VGGGV | ISGTSGLYS | QQSSNFPITF | TIGGYWIH | AIGPFFGTTMY | ARFNNWFWNVMDY |
| 23-08D | VGGGV | ISGTSGLYS | QQSSNFPITF | TIGGYWIH | GIGPFFGPTRY | ARFNNWFWNVMDY |
| 23-11D | VGGGV | ISGTSGLYS | QQSSNFPITF | TIGGYWIH | GIGPFFGPTRY | ARFNNWFWNVMDY |
| 23-11F | VGGGV | ISGTSGLYS | QQSSNFPITF | TIGGYWIH | GIGPFFGPTRY | ARFNNWFWNVMDY |
| 23-12F | VGGGV | ISGTSGLYS | QQSSNFPITF | TIGGYWIH | AIAPWWGDTPY | ARFNNWFWNVMDY |
| 24-01A | VGGGV | ISGTSGLYS | QQSSNFPITF | TIGGYWIH | GIGPFFGPTRY | ARFNNWFWNVMDY |
| 24-01B | VGGGV | ISGTSGLYS | QQSSNFPITF | TIGGYWIH | AIGPFFGTTMY | ARFNNWFWNVMDY |
| 24-01C | VGGGV | ISGTSGLYS | QQSSNFPITF | TIGGYWIH | AIGPFFGTTMY | ARFNNWFWNVMDY |
| 24-01D | VGGGV | ISGTSGLYS | QQSSNFPITF | TIGGYWIH | GIGPFFGPTRY | ARFNNWFWNVMDY |
| 24-01E | VGGGV | ISGTSGLYS | QQSSNFPITF | TIGGYWIH | GIGPFFGPTRY | ARFNNWFWNVMDY |
| 24-01F | VGGGV | ISGTSGLYS | QQSSNFPITF | TIGGYWIH | AIAPWWGDTPY | ARFNNWFWNVMDY |
| 24-01G | VGGGV | ISGTSGLYS | QQSSNFPITF | TIGGYWIH | GIGPFFGPTRY | ARFNNWFWNVMDY |
| 24-01H | VGGGV | ISGTSGLYS | QQSSNFPITF | TIGGYWIH | AIGPFFGTTMY | ARFNNWFWNVMDY |
| 24-02A | VGGGV | ISGTSGLYS | QQSSNFPITF | TIGGYWIH | AIGPFFGTTMY | ARFNNWFWNVMDY |
| 24-02B | VGGGV | ISGTSGLYS | QQSSNFPITF | TIGGYWIH | AIGPFFGTTMY | ARFNNWFWNVMDY |
| 24-02C | VGGGV | ISGTSGLYS | QQSSNFPITF | TIGGYWIH | GIGPFFGPTRY | ARFNNWFWNVMDY |
| 24-02D | VGGGV | ISGTSGLYS | QQSSNFPITF | TIGGYWIH | AIGPFFGTTMY | ARFNNWFWNVMDY |
| 24-02E | VGGGV | ISGTSGLYS | QQSSNFPITF | TIGGYWIH | GISPFLGITYY | ARFNNWFWNVMDY |
| 24-02F | VGGGV | ISGTSGLYS | QQSSNFPITF | TIGGYWIH | GIGPFFGPTRY | ARFNNWFWNVMDY |
| 24-02G | VGGGV | ISGTSGLYS | QQSSNFPITF | TIGGYWIH | AIGPFFGTTMY | ARFNNWFWNVMDY |
| 24-02H | VGGGV | ISGTSGLYS | QQSSNFPITF | TIGGYWIH | GIGPFFGPTRY | ARFNNWFWNVMDY |
| 24-05A | VGGGV | ISGTSGLYS | QQSSNFPITF | TIGGYWIH | AIGPFFGTTMY | ARFNNWFWNVMDY |
| 24-05D | VGGGV | ISGTSGLYS | QQSSNFPITF | TIGGYWIH | GIGPFFGPTRY | ARFNNWFWNVMDY |
| 24-05E | VGGGV | ISGTSGLYS | QQSSNFPITF | TIGGYWIH | AIAPFFGATKY | ARFNNWFWNVMDY |
| 24-05F | VGGGV | ISGTSGLYS | QQSSNFPITF | TIGGYWIH | AIGPFFGTTMY | ARFNNWFWNVMDY |
| 24-05G | VGGGV | ISGTSGLYS | QQSSNFPITF | TIGGYWIH | AIGPFFGTTMY | ARFNNWFWNVMDY |
| 24-06A | VGGGV | ISGTSGLYS | QQSSNFPITF | TIGGYWIH | GIGPFFGPTRY | ARFNNWFWNVMDY |
| 24-06B | VGGGV | ISGTSGLYS | QQSSNFPITF | TIGGYWIH | GIGPFFGPTRY | ARFNNWFWNVMDY |
| 24-06C | VGGGV | ISGTSGLYS | QQSSNFPITF | TIGGYWIH | AIAPWWGDTPY | ARFNNWFWNVMDY |
| 24-06E | VGGGV | ISGTSGLYS | QQSSNFPITF | TIGGYWIH | AIGPFFGTTMY | ARFNNWFWNVMDY |
| 24-06F | VGGGV | ISGTSGLYS | QQSSNFPITF | TIGGYWIH | AIGPFFGTTMY | ARFNNWFWNVMDY |
| 24-06G | VGGGV | ISGTSGLYS | QQSSNFPITF | TIGGYWIH | AIGPFFGTTMY | ARFNNWFWNVMDY |
| 24-06H | VGGGV | ISGTSGLYS | QQSSNFPITF | TIGGYWIH | GIGPFFGPTRY | ARFNNWFWNVMDY |
| 24-07A | VGGGV | ISGTSGLYS | QQSSNFPITF | TIGGYWIH | GIGPFFGPTRY | ARFNNWFWNVMDY |
| 24-07B | VGGGV | ISGTSGLYS | QQSSNFPITF | TIGGYWIH | GIGPFFGPTRY | ARFNNWFWNVMDY |
| 24-07C | VGGGV | ISGTSGLYS | QQSSNFPITF | TIGGYWIH | AIGPFFGTTMY | ARFNNWFWNVMDY |
| 24-07D | VGGGV | ISGTSGLYS | QQSSNFPITF | TIGGYWIH | GIGPFFGPTRY | ARFTNWFWNVMDY |
| 24-07E | VGGGV | ISGTSGLYS | QQSSNFPITF | TIGGYWIH | GIGPFFGPTRY | ARFNNWFWNVMDY |
| 24-07G | VGGGV | ISGTSGLYS | QQSSNFPITF | TIGGYWIH | AIGPFFGTTMY | ARFNNWFWNVMDY |
| 24-07H | VGGGV | ISGTSGLYS | QQSSNFPITF | TIGGYWIH | AIGPFFGTTMY | ARFNNWFWNVMDY |
| 24-08A | VGGGV | ISGTSGLYS | QQSSNFPITF | TIGGYWIH | GIGPFFGPTRY | ARFNNWFWNVMDY |
| H3-07A | VGGGV | ISGTSGLYS | QQSSNFPITF | TIGGYWIH | GIGPYWGSTYY | ARFNHLFWQVMDY |
| H3-07B | VGGGV | ISGTSGLYS | QQSSNFPITF | TIGGYWIH | GIGPYWGSTYY | ARFNHLFWQVMDY |
| H3-07C | VGGGV | ISGTSGLYS | QQSSNFPITF | TIGGYWIH | GIGPYWGSTYY | ARFNHLFWQVMDY |
| H3-07D | VGGGV | ISGTSGLYS | QQSSNFPITF | TIGGYWIH | GIGPYWGSTYY | ARFNHLFWQVMDY |
| H3-07H | VGGGV | ISGTSGLYS | QQSSNFPITF | TIGGYWIH | GIGPYWGSTYY | ARFNQYFFHVMDY |
| H3-08A | VGGGV | ISGTSGLYS | QQSSNFPITF | TIGGYWIH | GIGPYWGSTYY | ARFNHLFWQVMDY |
| H3-08B | VGGGV | ISGTSGLYS | QQSSNFPITF | TIGGYWIH | GIGPYWGSTYY | ARFNHLFWQVMDY |
| H3-08C | VGGGV | ISGTSGLYS | QQSSNFPITF | TIGGYWIH | GIGPYWGSTYY | ARFNHLFWQVMDY |
| H3-08D | VGGGV | ISGTSGLYS | QQSSNFPITF | TIGGYWIH | GIGPYWGSTYY | ARFNHLFWQVMDY |
| H3-08E | VGGGV | ISGTSGLYS | QQSSNFPITF | TIGGYWIH | GIGPYWGSTYY | ARFNQYFFHVMDY |
| H3-08F | VGGGV | ISGTSGLYS | QQSSNFPITF | TIGGYWIH | GIGPYWGSTYY | ARFNQYFFHVMDY |
| H3-08H | VGGGV | ISGTSGLYS | QQSSNFPITF | TIGGYWIH | GIGPYWGSTYY | ARVSINIFFHVDY |
| H3-09A | VGGGV | ISGTSGLYS | QQSSNFPITF | TIGGYWIH | GIGPYWGSTYY | ARFNHLFWQVMDY |
| H3-09B | VGGGV | ISGTSGLYS | QQSSNFPITF | TIGGYWIH | GIGPYWGSTYY | ARFNHLFWQVMDY |
| H3-09C | VGGGV | ISGTSGLYS | QQSSNFPITF | TIGGYWIH | GIGPYWGSTYY | ARFNHLFWQVMDY |
| H3-10B | VGGGV | ISGTSGLYS | QQSSNFPITF | TIGGYWIH | GIGPYWGSTYY | ARFNHLFWQVMDY |
| H3-10C | VGGGV | ISGTSGLYS | QQSSNFPITF | TIGGYWIH | GIGPYWGSTYY | ARFNHLFWQVMDY |
| H3-10D | VGGGV | ISGTSGLYS | QQSSNFPITF | TIGGYWIH | GIGPYWGSTYY | ARFNHLFWQVMDY |
| H3-11A | VGGGV | ISGTSGLYS | QQSSNFPITF | TIGGYWIH | GIGPYWGSTYY | ARFNHLFWQVMDY |
| H3-11B | VGGGV | ISGTSGLYS | QQSSNFPITF | TIGGYWIH | GIGPYWGSTYY | ARFNHLFWQVMDY |
| H3-11C | VGGGV | ISGTSGLYS | QQSSNFPITF | TIGGYWIH | GIGPYWGSTYY | ARFNHLFWQVMDY |
| H3-11D | VGGGV | ISGTSGLYS | QQSSNFPITF | TIGGYWIH | GIGPYWGSTYY | ARFNHLFWQVMDY |
| H3-12A | VGGGV | ISGTSGLYS | QQSSNFPITF | TIGGYWIH | GIGPYWGSTYY | ARFNHLFWQVMDY |
| H3-12B | VGGGV | ISGTSGLYS | QQSSNFPITF | TIGGYWIH | GIGPYWGSTYY | ARFNHLFWQVMDY |
| H3-12C | VGGGV | ISGTSGLYS | QQSSNFPITF | TIGGYWIH | GIGPYWGSTYY | ARFNHLFWQVMDY |
| H3-12D | VGGGV | ISGTSGLYS | QQSSNFPITF | TIGGYWIH | GIGPYWGSTYY | ARFNHLFWQVMDY |
| H3-12E | VGGGV | ISGTSGLYS | QQSSNFPITF | TIGGYWIH | GIGPYWGSTYY | ARFSQYFLHVMDY |
| H3-12F | VGGGV | ISGTSGLYS | QQSSNFPITF | TIGGYWIH | GIGPYWGSTYY | ARFNQYFFHVMDY |
| 25-01E | VGGGV | ISGTSGLYS | QQSSNFPITF | TIGGYWIH | GIGPYWGSTYY | ARFNHLFWQVMDY |
| 25-01G | VGGGV | ISGTSGLYS | QQSSNFPITF | TIGGYWIH | GIGPYWGSTYY | ARFNHLFWQVMDY |
| 25-08F | VGGGV | ISGTSGLYS | QQSSNFPITF | TIGGYWIH | GIGPYWGSTYY | ARFNHLFWQVMDY |
| 25-09G | VGGGV | ISGTSGLYS | QQSSNFPITF | TIGGYWIH | GIGPYWGSTYY | ARFNHLFWQVMDY |
| 25-10E | VGGGV | ISGTSGLYS | QQSSNFPITF | TIGGYWIH | GIGPYWGSTYY | ARFNHLFWQVMDY |
| 26-01A | VGGGV | ISGTSGLYS | QQSSNFPITF | TIGGYWIH | GIGPYWGSTYY | ARFNHLFWQVMDY |
| 26-01C | VGGGV | ISGTSGLYS | QQSSNFPITF | TIGGYWIH | GIGPYWGSTYY | ARFNHLFWQVMDY |
| 26-01E | VGGGV | ISGTSGLYS | QQSSNFPITF | TIGGYWIH | GIGPYWGSTYY | ARFNHLFWQVMDY |
| 26-01F | VGGGV | ISGTSGLYS | QQSSNFPITF | TIGGYWIH | GIGPYWGSTYY | ARFNHLFWQVMDY |
| 26-01G | VGGGV | ISGTSGLYS | QQSSNFPITF | TIGGYWIH | GIGPYWGSTYY | ARFNHLFWQVMDY |
| 26-01H | VGGGV | ISGTSGLYS | QQSSNFPITF | TIGGYWIH | GIGPYWGSTYY | ARFNHLFWQVMDY |
| 26-02A | VGGGV | ISGTSGLYS | QQSSNFPITF | TIGGYWIH | GIGPYWGSTYY | ARFNHLFWQVMDY |
| 26-02B | VGGGV | ISGTSGLYS | QQSSNFPITF | TIGGYWIH | GIGPYWGSTYY | ARFNHLFWQVMDY |
| 26-02C | VGGGV | ISGTSGLYS | QQSSNFPITF | TIGGYWIH | GIGPYWGSTYY | ARFNHLFWQVMDY |
| 26-02D | VGGGV | ISGTSGLYS | QQSSNFPITF | TIGGYWIH | GIGPYWGSTYY | ARFNHLFWQVMDY |
| 26-02E | VGGGV | ISGTSGLYS | QQSSNFPITF | TIGGYWIH | GIGPYWGSTYY | ARFNHLFWQVMDY |
| 26-02F | VGGGV | ISGTSGLYS | QQSSNFPITF | TIGGYWIH | GIGPYWGSTYY | ARFNHLFWQVMDY |
| 26-02G | VGGGV | ISGTSGLYS | QQSSNFPITF | TIGGYWIH | GIGPYWGSTYY | ARFNHLFWQVMDY |
| 26-02H | VGGGV | ISGTSGLYS | QQSSNFPITF | TIGGYWIH | GIGPYWGSTYY | ARFNHLFWQVMDY |
| 26-03A | VGGGV | ISGTSGLYS | QQSSNFPITF | TIGGYWIH | GIGPYWGSTYY | ARFNHLFWQVMDY |
| 26-03B | VGGGV | ISGTSGLYS | QQSSNFPITF | TIGGYWIH | GIGPYWGSTYY | ARFNHLFWQVMDY |
| 26-03C | VGGGV | ISGTSGLYS | QQSSNFPITF | TIGGYWIH | GIGPYWGSTYY | ARFNHLFWQVKDY |
| 26-03D | VGGGV | ISGTSGLYS | QQSSNFPITF | TIGGYWIH | GIGPYWGSTYY | ARFNHLFWQVMDY |
| 26-03E | VGGGV | ISGTSGLYS | QQSSNFPITF | TIGGYWIH | GIGPYWGSTYY | ARFNHLFWQVMDY |
| 26-03F | VGGGV | ISGTSGLYS | QQSSNFPITF | TIGGYWIH | GIGPYWGSTYY | ARFNHLFWQVMDY |
| 26-04C | VGGGV | ISGTSGLYS | QQSSNFPITF | TIGGYWIH | GIGPYWGSTYY | ARFNHLFWQVMDY |
| 26-04D | VGGGV | ISGTSGLYS | QQSSNFPITF | TIGGYWIH | GIGPYWGSTYY | ARCNDLDWRVMDY |
| 26-04E | VGGGV | ISGTSGLYS | QQSSNFPITF | TIGGYWIH | GIGPYWGSTYY | ARFNHLFWQVMDY |
| 26-04F | VGGGV | ISGTSGLYS | QQSSNFPITF | TIGGYWIH | GIGPYWGSTYY | ARFNHLFWQVMDY |
| 26-04G | VGGGV | ISGTSGLYS | QQSSNFPITF | TIGGYWIH | GIGPYWGSTYY | ARFNHLFWQVMDY |
| 26-05A | VGGGV | ISGTSGLYS | QQSSNFPITF | TIGGYWIH | GIGPYWGSTYY | ARFNHLFWHVMDY |
| 26-05B | VGGGV | ISGTSGLYS | QQSSNFPITF | TIGGYWIH | GIGPYWGSTYY | ARFNHLFWQVMDY |
| 26-05C | VGGGV | ISGTSGLYS | QQSSNFPITF | TIGGYWIH | GIGPYWGSTYY | ARFNHLFWQVMDY |
| 26-05D | VGGGV | ISGTSGLYS | QQSSNFPITF | TIGGYWIH | GIGPYWGSTYY | ARFNHLFWQVMDY |
| 26-05E | VGGGV | ISGTSGLYS | QQSSNFPITF | TIGGYWIH | GIGPYWGSTYY | ARFNHLFWQVMDY |
| 26-05F | VGGGV | ISGTSGLYS | QQSSNFPITF | TIGGYWIH | GIGPYWGSTYY | ARFNHLFWQVMDY |
| 26-06A | VGGGV | ISGTSGLYS | QQSSNFPITF | TIGGYWIH | GIGPYWGSTYY | ARFNHLFWQVMDY |
| 26-06B | VGGGV | ISGTSGLYS | QQSSNFPITF | TIGGYWIH | GIGPYWGSTYY | ARFNHLFWQVMDY |
| 26-06C | VGGGV | ISGTSGLYS | QQSSNFPITF | TIGGYWIH | GIGPYWGSTYY | ARFNHLFWQVMDY |
| 26-06E | VGGGV | ISGTSGLYS | QQSSNFPITF | TIGGYWIH | GIGPYWGSTYY | ARFNHLFWQVMDY |

**Supplemental References**

1 Lovell, S. C., Word, J. M., Richardson, J. S. & Richardson, D. C. The penultimate rotamer library. *Proteins: Structure, Function, and Bioinformatics* **40**, 389-408, doi:10.1002/1097-0134(20000815)40:3<389::aid-prot50>3.0.co;2-2 (2000).

2 Dunbrack, R. L. Rotamer Libraries in the 21st Century. *Current Opinion in Structural Biology* **12**, 431-440, doi:Doi: 10.1016/s0959-440x(02)00344-5 (2002).

3 Dunbrack, R. L., Jr. & Karplus, M. Conformational analysis of the backbone-dependent rotamer preferences of protein sidechains. *Nat Struct Biol* **1**, 334-340 (1994).

4 Kabsch, W. & Sander, C. Dictionary of protein secondary structure: pattern recognition of hydrogen-bonded and geometrical features. *Biopolymers* **22**, 2577-2637 (1983).

5 Kleywegt, G. J. Quality control and validation. *Methods Mol Biol* **364**, 255-272, doi:10.1385/1-59745-266-1:255 (2007).

6 Bezdek, J. *Pattern Recognition with Fuzzy Objective Function Algorithms (Advanced Applications in Pattern Recognition)*. (Springer, 1981).

7 Bensaid, A. M. H., L.O.; Bezdek, J.C.; Clarke, L.P.; Silbiger, M.L.; Arrington, J.A.; Murtagh, R.F. Validity-guided (re)clustering with applications to image segmentation. *IEEE Transactions on Fuzzy Systems* **4**, 22, doi:10.1109/91.493905 (1996).

8 Laskowski, R. A., Thornton, J. M., Humblet, C. & Singh, J. X-SITE: Use of Empirically Derived Atomic Packing Preferences to Identify Favourable Interaction Regions in the Binding Sites of Proteins. *Journal of Molecular Biology* **259**, 175-201, doi:DOI: 10.1006/jmbi.1996.0311 (1996).

9 Levy, E. D., Pereira-Leal, J. B., Chothia, C. & Teichmann, S. A. 3D Complex: A Structural Classification of Protein Complexes. *PLoS Comput Biol* **2**, e155 (2006).

10 Connolly, M. Analytical molecular surface calculation. *Journal of Applied Crystallography* **16**, 548-558, doi:doi:10.1107/S0021889883010985 (1983).

11 Hsu, H.-J. *et al.* Assessing Computational Amino Acid ²-Turn Propensities with a Phage-Displayed Combinatorial Library and Directed Evolution. *Structure (London, England : 1993)* **14**, 1499-1510 (2006).

12 Gorodkin, J., Heyer, L. J., Brunak, S. & Stormo, G. D. Displaying the information contents of structural RNA alignments: the structure logos. *Comput Appl Biosci* **13**, 583-586 (1997).

13 Fuh, G. *et al.* Structure-Function Studies of Two Synthetic Anti-vascular Endothelial Growth Factor Fabs and Comparison with the Avastin™ Fab. *Journal of Biological Chemistry* **281**, 6625-6631, doi:10.1074/jbc.M507783200 (2006).

14 Yu, C. M. *et al.* Rationalization and design of the complementarity determining region sequences in an antibody-antigen recognition interface. *PLoS One* **7**, e33340, doi:10.1371/journal.pone.0033340 [doi]

PONE-D-11-14795 [pii] (2012).

15 Schymkowitz, J. *et al.* The FoldX web server: an online force field. *Nucleic Acids Res* **33**, W382-388, doi:10.1093/nar/gki387 (2005).
